# Cross Kingdom Metabolic Engineering Paradigm Elevating Sustainable Protein Production

**DOI:** 10.1002/advs.202517703

**Published:** 2026-06-23

**Authors:** Yuanyuan Du, Changyu Pi, Kai Hong, Le Gao, Xin Wu

**Affiliations:** ^1^ State Key Laboratory of Engineering Biology for Low‐Carbon Manufacturing Tianjin Institute of Industrial Biotechnology Chinese Academy of Sciences Tianjin China

**Keywords:** asparagine synthetase, carbon metabolism, methanol, nitrogen assimilation, *Pichia pastoris*, single cell protein

## Abstract

Confronting the dual crisis of escalating global protein demand and unsustainable agriculture necessitates transformative solutions. Here, we pioneer evolutionary insights from maize nitrogen optimization via asparagine synthetase (ASNS) to rewire metabolism in *Pichia pastoris*. Empirically, the tri‐copy ASNS strain achieved superior protein titers: 62.48% crude protein, 47.86% total amino acids, and 8.05% branched‐chain amino acids, nutritionally surpassing conventional protein sources. Genome‐scale modeling and transcriptomic studies provided convergent evidence that ASNS overexpression drove global metabolic rewiring through predicted synergistic coupling between aspartate metabolism and the tricarboxylic acid cycle. Mechanistically, ASNS overexpression unlocked a previously uncharacterized nitrogen sensor‐regulator circuit by inducing *PAS_chr1‐1_0158*, validated in amplifying intracellular nitrogen flux and driving a self‐reinforcing cycle of ammonia assimilation. This work validated evolutionary conservation of nitrogen optimization strategies across kingdoms, established the first scalable blueprint for carbon–nitrogen co‐optimized microbial cell factories, decouples sustainable SCP production from agricultural constraints, and offers a scalable solution to the global protein crisis.

## Graphical abstract

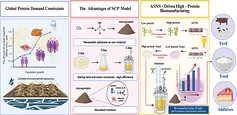

## 1 Introduction

As the global population surpasses 9.7 billion, the demand for global terrestrial animal source food is projected to grow by 20% by 2050 [1, 2]. Humanity faces a paradoxical crisis at the nexus of SDG2 (Zero Hunger) and SDG13 (Climate Action). This “protein trilemma” intensifies as climate change causes a significant reduction in crop yields while demand requires 20% more protein by 2050 [3, 4]. Traditional agriculture, a sector that is highly reliant on arable land, consuming up to 70% of freshwater, has witnessed only a 10% increase in productivity over the past six decades [5]. Therefore, single‐cell protein (SCP) derived from non‐grain feedstocks and microorganisms holds great potential to tackle global challenges associated with food security, resource scarcity, and environmental sustainability [6].

The development of carbon dioxide (CO_2_) hydrogenation technology offers a promising route to sustainable methanol supply, positioning it as an attractive feedstock for bio‐based manufacturing [7, 8]. *Pichia pastoris* (*P. pastoris*), a natural methylotroph, has emerged as an ideal host for sustainable SCP production due to its unique ability to utilize methanol, coupled with the integration of genetic engineering and artificial intelligence technologies [9, 10]. However, realizing the full potential of methanol‐based SCP production in *P. pastoris* faces significant metabolic bottlenecks. The intricate compartmentalization and regulation of its methanol assimilation pathway result in suboptimal carbon flux distribution and significant metabolic inefficiencies. Crucially, this inherent carbon‐centric bottleneck severely restricts the availability of carbon skeletons and energy necessary to support high‐efficiency nitrogen assimilation and protein biosynthesis [11, 12].

It is well‐established that cellular life requires intricate coordination between carbon and nitrogen metabolism, with each profoundly influencing the other. Carbon flux not only responds to carbon source availability but is also critically modulated by nitrogen status, as demonstrated across diverse microbial systems [13]. For instance, *Escherichia coli* mutants defective in nitrogen signaling (ptsN) exhibited growth failure under ammonium limitation despite sugar availability [14], while *Bacillus subtilis* strains lacking carbon catabolite control (CcpA) suffer severe nitrogen assimilation defects that hinder growth‐an impairment only remediable by supplementation with specific amino acids like branched‐chain amino acids and methionine [15]. These examples underscore a fundamental constraint, particularly inefficient nitrogen utilization directly cripples an organism's capacity to harness carbon for growth and biosynthesis‐a bottleneck acutely relevant to microbial protein production. This interdependency presents a critical challenge for *P. pastoris* as a methanol‐based SCP host. Nitrogen serves as the atomic backbone of amino acid biosynthesis, and its flux dictates proteogenic capacity‐the very metric defining SCP value. While *P. pastoris* excels in methanol assimilation, its inherent metabolic architecture imposes limitations on nitrogen conversion efficiency. Consequently, maximising protein yield from methanol remains an unmet goal, constrained by suboptimal carbon–nitrogen synergy.

Here, we explore nature's evolutionary solutions for nitrogen optimization. Pioneering work by Huang et al. (2022) identified a master regulator of nitrogen use efficiency (NUE) in teosinte (*Zea mays subsp. parviglumis*), the wild ancestor of maize [16]. The locus THP9 encodes asparagine synthetase (ASNS), a metabolic linchpin that coordinates nitrogen cycling via asparagine biosynthesis. Transgenic restoration of THP9 in modern maize elevated grain proteins by 30% without a yield penalty, demonstrating ASNS's power to bypass trade‐offs between nitrogen allocation and fitness. Crucially, ASNS overexpression enhances NUE even under nutrient stress, compensating for soil nitrogen deficits [17]. The phylogenetic conservation of ASNS function across eukaryotes [18] presents a revolutionary opportunity. Repurposing this plant‐evolved nitrogen optimization machinery within a microbial cell factory.

**FIGURE 1:**
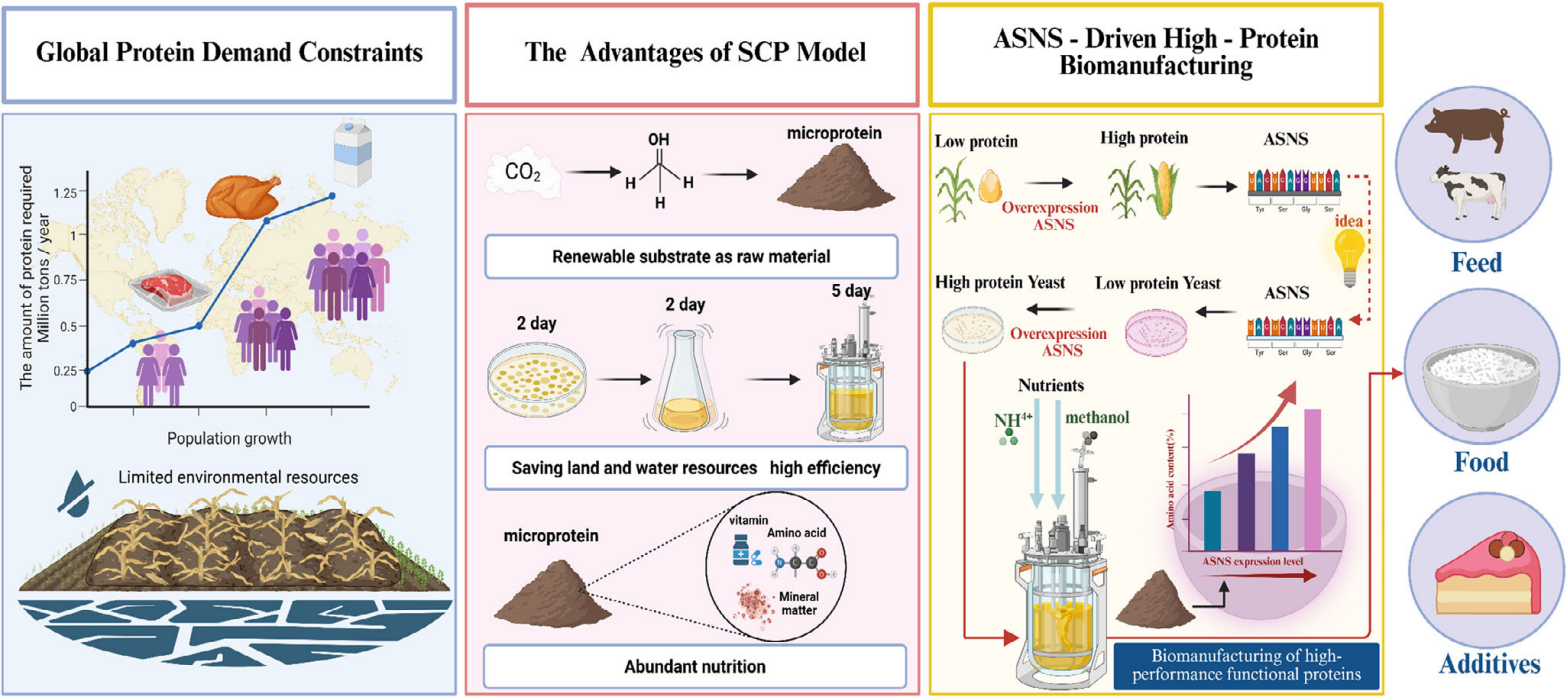
Innovative solutions and metabolic engineering strategies to cope with protein demand pressure.

This study implemented a cross‐kingdom metabolic engineering strategy, building upon nature's evolutionary wisdom‐where the THP9 locus enhances maize seed protein content through ASNS‐mediated nitrogen assimilation. Leveraging phylogenetic insights into the conserved function of ASNS across eukaryotes, we reprogrammed the methylotrophic yeast *P. pastoris* into a highly efficient microbial protein factory (Figure 1). This engineering strategy would create a synthetic biology platform capable of fundamentally rewiring intrinsic carbon–nitrogen coupling within the host. We pioneered evolutionary insights from maize nitrogen optimization via ASNS to rewire metabolism in the methylotrophic yeast *P. pastoris*. Beyond its immediate application, this work reveals deeply conserved mechanisms governing carbon–nitrogen metabolic synergy across biological kingdoms, providing an actionable blueprint for constructing next‐generation microbial cell factories dedicated to sustainable SCP production.

## 2 Results

### 2.1 Phylogenetic and Functional Conservation Analysis of ASNS Genes

**FIGURE 2:**
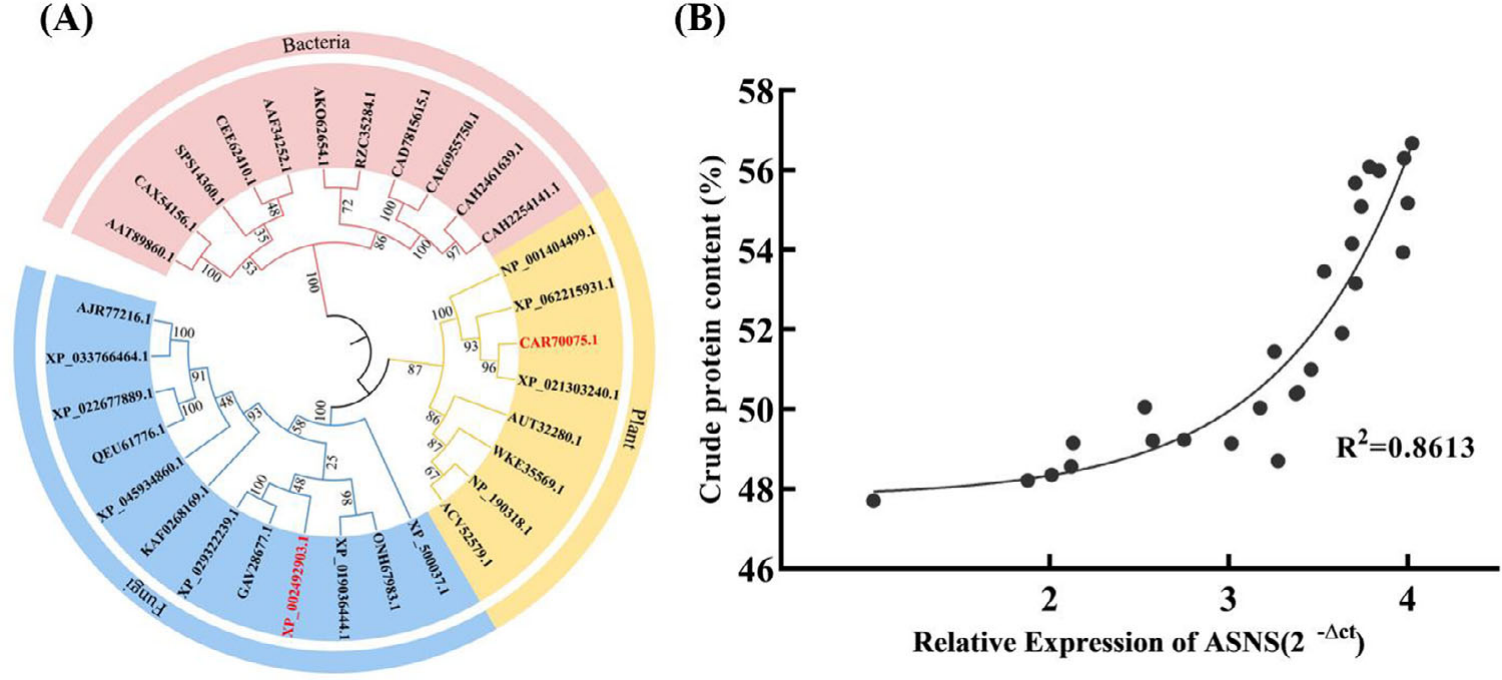
Presents a comprehensive analysis of the ASNS gene across different species, focusing on its phylogenetic and functional characteristics. (A) The phylogenetic tree of ASNS was drawn by the maximum likelihood method. (B)The correlation analysis between the expression of ASNS and the crude protein content of different yeast strains. Each data point represents an individual yeast strain. The curve represents the fitted curve (y = 0.034e^1.379x^ + 47.797).

Regarding the exact role of the ASNS gene (*PAS_chr1‐1_0158*, C4QW99), the bioinformatic analysis indicated that it contained six transmembrane regions, suggesting it was a putative transmembrane protein likely localized to the membrane, rather than a typical transcription factor, enzyme, or kinase. Its current annotation in the database describes it as a putative transmembrane protein involved in the export of ammonia, a starvation signal. Phylogenetic reconstruction of ASNS genes across three domains of life revealed distinct evolutionary trajectories between prokaryotic and eukaryotic lineages (Figure 2A). The observed clustering pattern‐with plants and fungi forming a cohesive clade from bacterial counterparts‐provides molecular validation for the monophyletic origin of eukaryotes [19]. This evolutionary relationship is further supported by fundamental functional divergences: prokaryotic ASNA‐family enzymes exhibit restricted substrate specificity (ammonium‐dependent L‐aspartate amidation), whereas their eukaryotic ASNB counterparts demonstrate metabolic versatility through dual utilization of glutamine and ammonium ions as amino donors [20]. Cross‐kingdom sequence alignment revealed remarkable structural preservation between plant and fungal ASNS orthologs; critical functional domains including catalytic sites and substrate‐binding regions, which showed >85% sequence identity (Figures S1 and S2).

The functional significance of this conservation is underscored by plant studies demonstrating ASNS's pivotal role in the nitrogen economy. Transgenic overexpression in *Zea mays*, *Arabidopsis* thaliana, and *Oryza sativa* consistently enhanced tissue protein content through improved nitrogen assimilation efficiency [16, 21, 22]. These findings establish ASNS as a master regulator of nitrogen flux partitioning between storage (asparagine) and anabolic pathways (protein synthesis)‐a function closely related to our study's hypotheses. Given the conserved catalytic apparatus and nitrogen management challenges shared by photoautotrophic plants and heterotrophic fungi, we posit that fungal ASNS orthologs may orchestrate analogous metabolic decisions‐channeling assimilated nitrogen into storage polypeptides while optimising biosynthetic precursor availability, which supports our engineering strategy.

### 2.2 Correlation Analysis Between ASNS Expression and Crude Protein Content in Different Yeast Strains

Systematic multi‐strain correlation analysis was performed using a comprehensive strain collection to define the mechanistic linkage between dynamic ASNS expression and crude protein biosynthesis in yeast. Quantitative correlation analysis demonstrated a robust positive association between ASNS transcript abundance and crude protein content (R^2^ = 0.8613, *p *< 0.001), establishing ASNS as a key determinant of proteogenic capacity in yeast systems (Figure 2B).

### 2.3 Optimized Nitrogen‐To‐Protein Conversion by Strategic Genomic Integration

**FIGURE 3:**
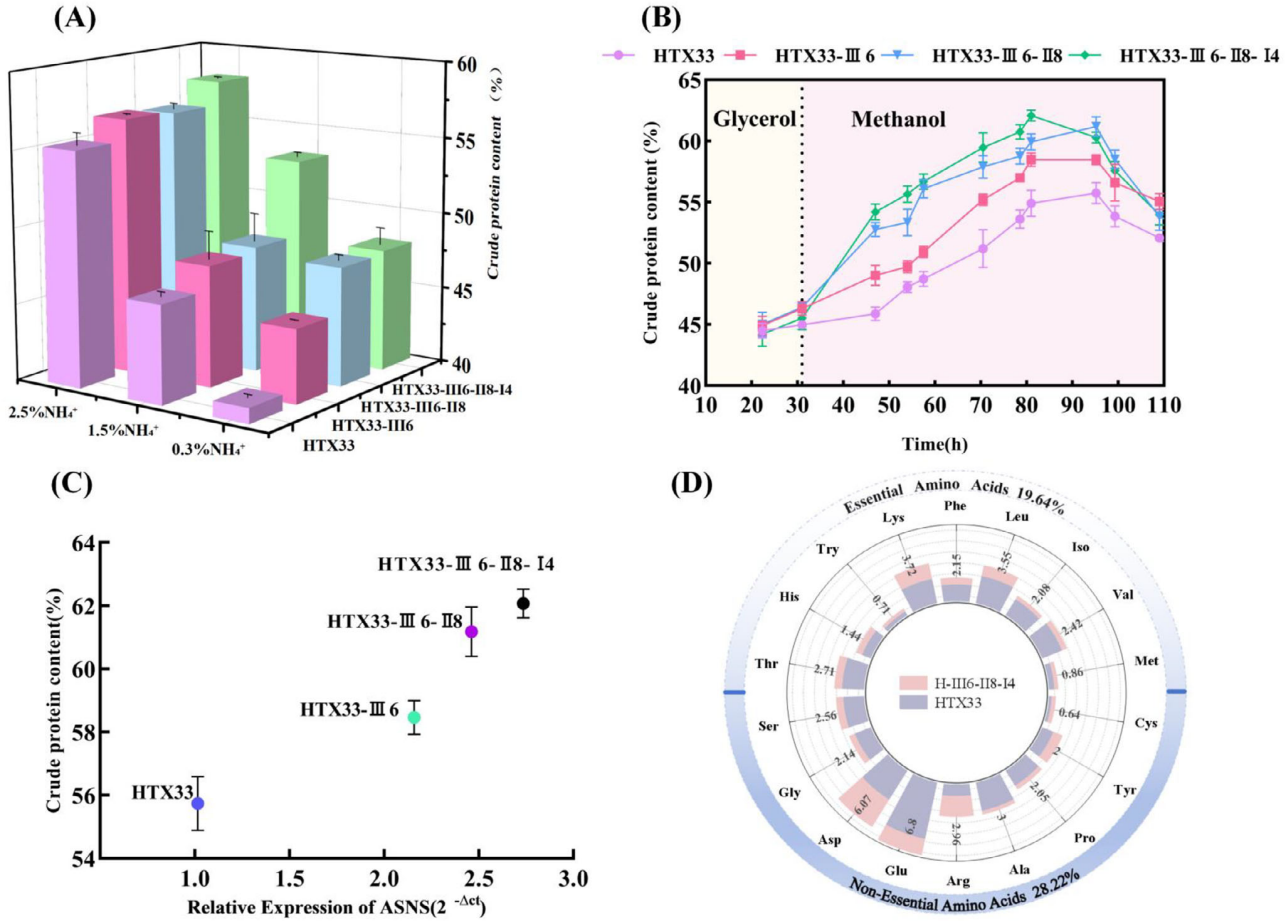
Details the experimental outcomes of ASNS engineering in *P. pastoris*, evaluating protein content under varying conditions. (A) Crude protein content of multi‐copy strains under different NH_4_^+^ ion concentrations. (B) The crude protein content of strains with different ASNS copy numbers was determined through fed‐batch fermentation in a 5L bioreactor. (C) The relationship between ASNS expression and cellular protein content in strains with varying ASNS copy numbers. (D)Amino acid content of HTX33 and HTX33‐III6‐II8‐ I4 strains.

To mitigate pleiotropic effects on cellular homeostasis, we implemented targeted ASNS overexpression through chromosomal integration at validated neutral loci (PNSI‐4, PNSIII‐6, and PNSII‐8) that maintain genomic stability while permitting high‐efficiency (100%) heterologous expression [23]. This orthogonal engineering approach generated isogenic *P. pastoris* variants with progressive ASNS copy number amplification (HTX33‐III6, HTX33‐III6‐II8, and HTX33‐III6‐II8‐I4). Under controlled methanol‐C/NH_4_^+^‐N conditions in shake‐flask cultures, quantitative proteomic analysis revealed a direct correlation (R^2^ = 0.92, *p *< 0.01) between ammonium concentration (0.3%–2.5% w/v) and nitrogen‐to‐protein conversion efficiency across all genotypes (Figure 3A). The tri‐copy strain HTX33‐III6‐II8‐I4 exhibited maximal crude protein accumulation (58.0 ± 0.1%) at 2.5% NH_4_^+^, demonstrating 6% enhancement over basal nitrogen assimilation capacity.

Scale‐up validation in 5L bioreactors under fed‐batch conditions unveiled remarkable bioprocessing advantages. The engineered strains displayed dose‐dependent protein titers increasing from 58.47 ± 0.3% (single‐copy) to 62.07 ± 0.5% (tri‐copy), representing 11.4% absolute improvement over wild‐type (55.74 ± 0.7%) (Figure 3B). Notably, the tri‐copy variant achieved peak protein content (62.07%) within 81 h, which was 15% faster than the parental strain maturation kinetics. This metabolic acceleration mirrors ASNS‐mediated nitrogen flux enhancements observed in *Zea mays* [16], confirming evolutionary conservation of ASNS regulatory paradigms across eukaryotic systems.

### 2.4 Correlation Analysis and Amino Acid Profile of ASNS Gene Expression in Engineered *P. pastoris*

To further validate the correlation between ASNS gene expression and cellular protein content, we performed quantitative real‐time PCR to measure ASNS transcription levels across different engineered strains, coupled with Kjeldahl determination of crude protein content. Statistical analysis demonstrated a strong positive correlation (*p* < 0.05) between ASNS transcript abundance and cellular protein accumulation (Figure 3C). Notably, the triple‐copy recombinant strain exhibited a 2.7‐fold increase in ASNS mRNA levels compared to wild‐type HTX33, corresponding to a substantial elevation in protein content. This phenotypic enhancement likely stems from ASNS‐mediated optimization of nitrogen metabolism, where improved ammonium assimilation efficiency synergizes with enhanced amino acid biosynthesis to drive proteome expansion.

The amino acid profile analysis revealed transformative improvements in SCP derived from engineered *P. pastoris*. Comprehensive amino acid profiling of the HTX33‐III6‐II8 strain demonstrated a 1.48‐fold increase in total amino acid content (47.86% of biomass) compared to parental controls (Figure 3D). The SCP contained nutritionally complete profiles of nine essential amino acids: isoleucine (2.08%), leucine (3.55%), lysine (3.72%), methionine (0.86%), phenylalanine (2.15%), threonine (2.71%), arginine (2.31%), valine (2.42%), and histidine (1.44%). Furthermore, branched‐chain amino acids (BCAAs) constituted 8.05% of total amino acids (16.8% of detected species), reaching concentrations with demonstrated therapeutic potential.

### 2.5 Transcriptome Profiling Elucidated the Regulatory Mechanisms Underlying ASNS Overexpression

**FIGURE 4:**
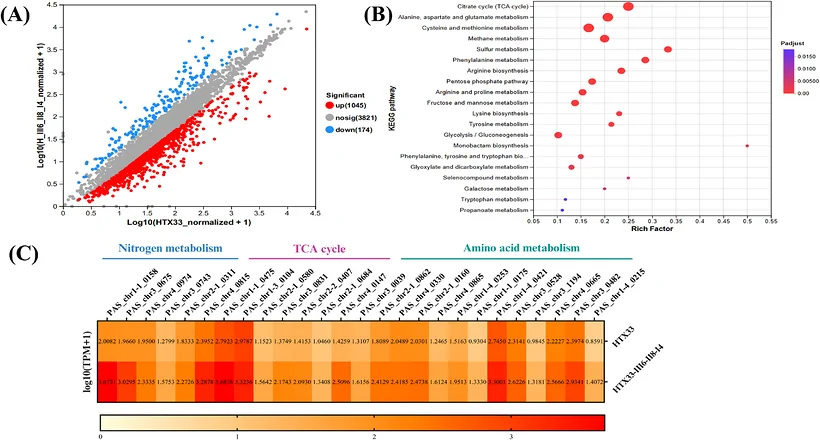
Provides transcriptomic insights into the metabolic rewiring induced by ASNS overexpression. (A) Differential expression plot. (B) KEGG enrichment plot. (C) Heatmap showing differential expression of genes associated with nitrogen metabolism, TCA cycle, and amino acid metabolism.

Comparative transcriptomic profiling between the wild‐type HTX33 strain and the engineered HTX33‐III6‐II8‐I4 variant to mechanistically dissect how ASNS overexpression augments SCP biosynthesis in *P. pastoris*, Our analysis revealed marked transcriptional reprogramming, with 1,219 differentially expressed genes (DEGs; |log_2_FC|≥1, q ≤ 0.05) including 1,045 upregulated and 174 downregulated targets (Figure 4A, Table S1). Notably, the gene *PAS_chr1‐1_0158* was significantly upregulated, which is annotated as a potential nitrogen starvation signal molecule but has never been functionally validated. We speculated that this upregulation may be due to the overexpression of ASNS, which leads to a mismatch between the actual metabolic intensity of nitrogen fixation and the overexpression level of ASNS, thereby activating a nitrogen starvation signal to enhance nitrogen assimilation. This transcriptional activation likely reflects metabolic compensation for intracellular nitrogen imbalance induced by ASNS overexpression, as evidenced by coordinated induction of nitrogen salvage pathways: (i) Enhanced expression of *PAS_chr3_0743* (NIT2/yafV, ω‐amidase) and *PAS_chr2‐1_0311* (GDH2, glutamate dehydrogenase) promoted glutamate/glutamine catabolism to replenish nitrogen pools; (ii) Upregulated *PAS_chr4_0974* (GOT1, aspartate aminotransferase) increased aspartate production for ammonia fixation (Figure 4C). Mechanistically, ASNS‐mediated nitrogen assimilation operates through dual substrate utilization, while the canonical glutamine synthetase (GS) pathway converts ammonia to glutamine‐derived asparagine [20]. Our data suggested direct ammonium assimilation via ASNS creates a metabolic bypass that enhances nitrogen flux. Collectively, these findings demonstrate that ASNS overexpression triggers a nitrogen starvation response that: (1) activates nitrogen recycling machinery through *PAS_chr1‐1_0158* signaling, (2) establishes parallel ammonia assimilation routes via GS and ASNS pathways, and (3) ultimately optimizes nitrogen utilization efficiency through accelerated metabolic cycling.

**FIGURE 5:**
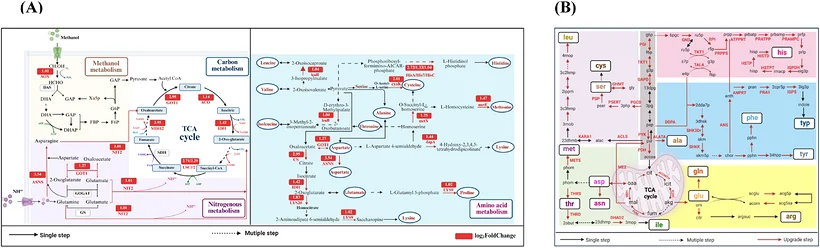
Metabolic rewiring of carbon and nitrogen metabolism triggered by ASNS overexpression. (A) Carbon and nitrogen synergistic metabolic pathway. Metabolic pathway in amino acid metabolism. Genes in red showed significant upregulation in expression. (B) employs the genome‐scale metabolic network model to predict the outcomes of amino acid metabolism following ASNS overexpression, forecasting enhanced flux redistribution and biosynthetic capacity across proteogenic amino acids.

Furthermore, transcriptome analysis revealed a significant upregulation of key enzymes in the TCA cycle, including gene‐*PAS_chr4_0815* (encoding MDH2, malate dehydrogenase), gene‐*PAS_chr1‐1_0475* (encoding CS, citrate synthase), gene‐*PAS_chr1‐3_0104* (encoding ACO, aconitate hydratase), gene‐*PAS_chr2‐1_0580* (encoding IDH1/IDH2/icd, isocitrate dehydrogenase), gene‐*PAS_chr3_0831* (encoding LSC1, succinyl‐CoA synthetase alpha subunit), and gene‐*PAS_chr2‐2_0407* (encoding LSC2, succinyl‐CoA synthetase beta subunit) (Figures 4C and 5A). This indicated that ASNS overexpression may enhance nitrogen cycling, leading to the production of more TCA cycle intermediates and subsequently activating the TCA cycle, thereby providing sufficient energy for cell anabolism. Collectively, ASNS overexpression orchestrated synergistic carbon–nitrogen metabolic coupling by simultaneously enhancing ammonia assimilation, accelerating nitrogen cycling, and activating the TCA cycle. These findings established a novel mechanistic paradigm wherein ASNS functions as a master regulator of metabolic resource allocation, fundamentally deepening understanding of cellular nitrogen homeostasis while providing transformative insights for engineering carbon–nitrogen co‐utilization systems.

KEGG enrichment analysis revealed that a total of 87 pathways were enriched (Table S2). Among them, amino acid metabolic pathways were significantly impacted (Figure 4B), with the expression levels of multiple genes involved in amino acid synthesis and modification varying. First, synthesis of branched‐chain amino acids was enhanced, in agreement with the above results of amino acid content. The upregulation of gene‐*PAS_chr2‐1_0684* (encoding hisC, histidinol‐phosphate aminotransferase), gene‐*PAS_chr4_0147* (encoding ARO9, aromatic amino acid aminotransferase II), and gene‐*PAS_chr3_0039* (encoding IMDH, 3‐isopropylmalate dehydrogenase), respectively, promoted the synthesis of histidine, aromatic amino acids, and leucine. As essential components of protein synthesis, the increased content of these branched‐chain amino acids provided a guarantee for protein diversity. Second, the synthesis of sulfur‐containing amino acids was also promoted. The upregulation of gene‐*PAS_chr2‐1_0862* (encoding CS, cysteine synthase), gene‐*PAS_chr4_0330* (encoding MET17, *O*‐acetylhomoserine), Gene‐*PAS_chr2‐1_0160* (encoding metE, 5‐methyltetrahydropteroyltriglutamate–homocysteine methyltransferase), gene‐*PAS_chr4_0865* (encoding metX, *O*‐succinyltransferase), and gene‐*PAS_chr1‐4_0253* (encoding SAT, sulfate adenylyltransferase) jointly promoted the synthesis of cysteine and methionine. These sulfur‐containing amino acids are crucial for maintaining protein structure and function. Additionally, the upregulation of gene‐*PAS_chr1‐1_0175* (encoding dapA, 4‐hydroxy‐tetrahydrodipicolinate synthase), gene‐*PAS_chr1‐4_0421* (encoding HCS, homocitrate synthase), and gene‐*PAS_chr3_0528* (encoding LYS9, saccharopine dehydrogenase) promoted the synthesis of lysine. The upregulation of gene‐*PAS_chr3_1194* (encoding KYNU, kynureninase) promoted tryptophan metabolism. The upregulation of gene‐*PAS_chr4_0665* (encoding G5SDH, glutamate‐5‐semialdehyde dehydrogenase) promoted the synthesis of proline (Figures 4C and 5A).

This comprehensive transcriptional activation identifies ASNS as a master coordinator of amino acid biosynthesis, supplying ample precursors for proteome expansion. We propose that ASNS optimizes carbon–nitrogen metabolic flux to boost energy (ATP/NADPH) and carbon skeletons, forming a self‐reinforcing cycle that maximizes amino acid production efficiency. These insights redefine ASNS's role in eukaryotic metabolic regulation and provide actionable strategies for engineering high‐efficiency microbial bioproduction systems. The causal relationship between ASNS overexpression and TCA cycle activation, as inferred from transcriptomic correlation and genome‐scale modeling predictions, remains to be empirically validated through targeted metabolomics. The observed transcriptional upregulation of TCA enzymes (MDH2, CS, ACO, IDH1/2, LSC1/2) establishes a robust correlation. However, direct measurement of metabolic flux through the aspartate‐oxaloacetate node and subsequent TCA intermediates is necessary to confirm the predicted metabolic rewiring.

To directly test whether ASNS overexpression altered intracellular nitrogen status, we quantified two core nitrogen assimilation intermediates such as glutamate (Glu) and glutamine (Gln) in *P. pastoris* HTX33 and ASNS‐engineered *P. pastoris* strains during the exponential protein synthesis phase. Consistent with ASNS's requirement for Gln as an amino donor, intracellular Gln content increased in a copy‐number‐dependent manner (Figure S3): the tri‐copy strain (HTX33‐III6‐II8‐I4) reached 4120.27 mg/L under 2.5% ammonium, 1.69‐fold higher than that of HTX33 (2466.61 mg/L). In contrast, Glu levels remained stable across all strains and ammonium conditions (172.76–260.21 mg/L, Figure S3), reflecting its role as a homeostatic central node for carbon‐nitrogen coupling. The Gln/Glu ratio as a canonical proxy for nitrogen assimilation flux exhibited a progressive increase that correlated tightly with both ASNS copy number and extracellular ammonium availability. At 2.5% ammonium, the tri‐copy ASNS‐engineered *P. pastoris* reached a ratio of 20.02, nearly 1.7‐fold higher than the wild‐type *P. pastoris* of 11.79, directly reflecting accelerated nitrogen turnover and flux redistribution driven by ASNS overexpression. ASNS‐driven asparagine synthesis accelerates Gln consumption, which was compensated by upregulated Gln synthesis (consistent with transcriptomic activation of GDH2, GOT1, and NIT2), creating a dynamic mismatch between nitrogen demand and supply capacity. This metabolic imbalance likely acts as the cue for *PAS_chr1‐1_0158* activation, validating our original hypothesis of nitrogen status perturbation.

### 2.6 Genome‐Scale Metabolic Network Modeling Predicts ASNS Overexpression Orchestrates Global Metabolic Remodeling via Aspartate‐TCA Cycle Synergism

Genome‐scale metabolic network modeling of flux redistribution under ASNS overexpression demonstrates a sophisticated system‐level reorganization of cellular metabolism. The intervention induced remarkable metabolic restructuring characterized by enhanced aspartate channeling into the TCA cycle, particularly amplifying the biochemical coupling between aspartate and oxaloacetate nodes. This flux reprogramming revealed dynamic regulation of TCA cycle throughput that fundamentally reshapes cellular energy transduction and carbon skeleton allocation. Beyond direct modulation of aspartate‐related pathways, computational simulations predicted comprehensive metabolic rewiring through strengthened crosstalk between central carbon metabolism and amino acid biosynthesis networks. At the amino acid metabolome level, computational flux analysis revealed ASNS‐driven amplification of biosynthetic capacity across 15 proteogenic amino acids, spanning both direct precursor coupling and indirect network‐level modulation. This systemic biosynthetic enhancement originated from ASNS‐mediated elevation of aspartate metabolic flux, generating surplus precursor pools that fuel diversified amino acid production cascades. Notably, canonical aspartate‐family pathways (threonine/methionine biosynthesis) show pronounced flux increases (Figure 5B). Collectively, our multi‐omics interrogation established ASNS overexpression as a master regulator of cellular metabolic topology. By forging reinforced aspartate‐TCA cycle interconnectivity, this intervention orchestrates a quantum leap in metabolic efficiency: the TCA hub dynamically reconfigures to simultaneously optimise energy transduction efficiency and amino acid bioproduction fidelity. This elegant coupling of carbon skeleton routing with nitrogen assimilation precision unveils previously unrecognised homeostatic control architectures governing microbial proteome expansion. It is important to note that these predicted flux redistributions are derived from in silico constraint‐based optimization and await direct experimental validation. While transcriptomic upregulation of TCA cycle genes (Figure 4C) provides strong correlative support for the proposed aspartate‐TCA cycle coupling, direct measurement of metabolic flux through the aspartate‐oxaloacetate node and subsequent TCA intermediates is necessary to confirm the predicted metabolic rewiring. In follow‐up studies, we will combine targeted metabolomics of key TCA intermediates and ^13^C‐labeled metabolic flux analysis to quantify carbon flux distribution and verify the causal linkage between aspartate metabolism and TCA cycle activation. These unresolved mechanistic layers represent priority targets for deepening our understanding of carbon‐nitrogen synergy in methylotrophic yeasts.

### 2.7 *PAS_chr1‐1_0158* Regulates Nitrogen Utilization and Protein Accumulation in Yeast in Response to Ammonium Availability

**FIGURE 6:**
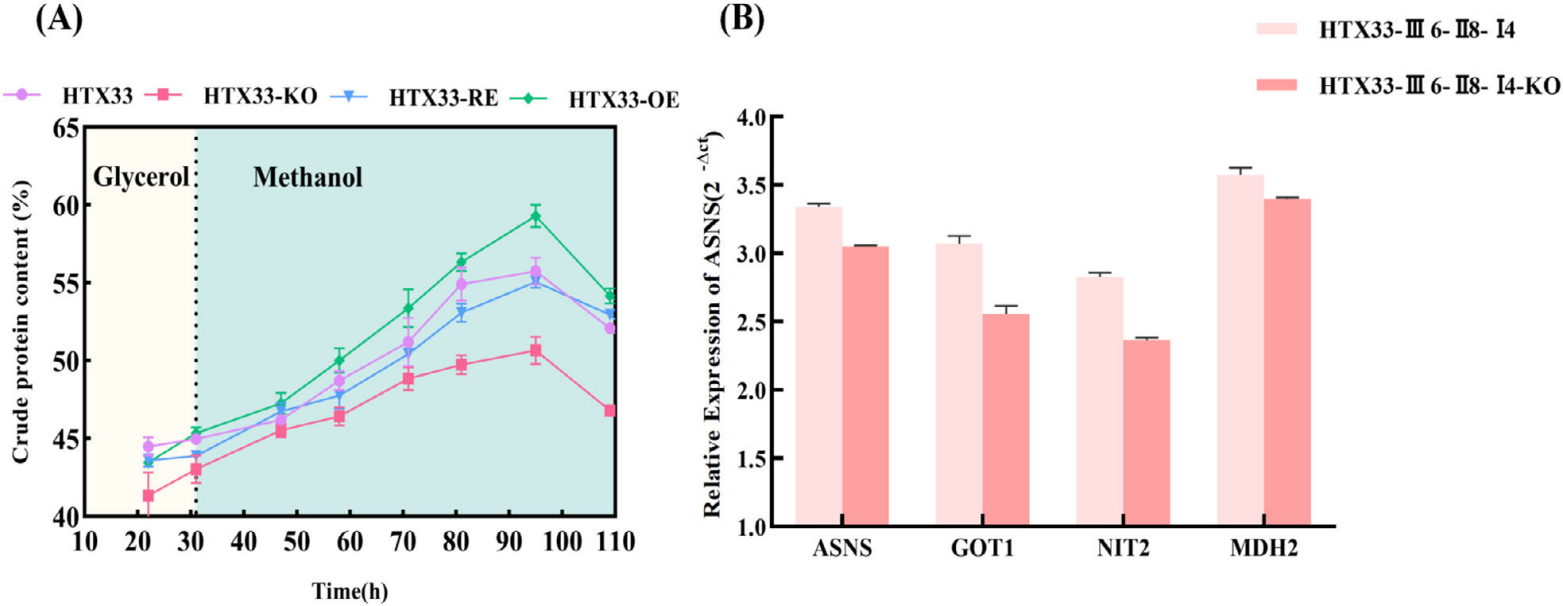
Functional validation of *PAS_chr1‐1‐0158* as a nitrogen‐responsive regulator. (A) Crude protein content over time in HTX33 wild‐type, *PAS_chr1‐1‐0158* knockout (KO), complementation (RE), and overexpression (OE) strains during fed‐batch fermentation (5 L bioreactor). Glycerol (0–30 h) and methanol induction (30–110 h) phases are indicated. Mean ± SD, n = 3. (B) Relative transcription levels of core nitrogen metabolism and TCA cycle genes in the *PAS_chr1‐1_0158* knockout strain (HTX33‐III6‐II8‐I4‐KO) and its parental ASNS tri‐copy overexpression strain. The reduced ASNS expression in the knockout strain indicates a close regulatory correlation between the two genes, and two potential regulatory models are proposed in the main text. ACT1 was used as the reference gene. Data are presented as mean ± SD (n = 3).

Previously annotated as a putative nitrogen starvation signaling molecule yet functionally unvalidated, *PAS_chr1‐1_0158* was experimentally confirmed in this study to orchestrate nitrogen‐responsive protein biosynthesis. Using engineered *P.pastoris* strains (HTX33, HTX33‐KO, HTX33‐RE, and HTX33‐OE) in 5 L bioreactor systems, we elucidated its critical role in NUE. An orthogonal genetic perturbation strategy revealed a concentration‐dependent protein accumulation pattern across all genotypes: increasing ammonium availability enhanced cellular protein content in every strain, with the strongest response observed in the overexpression (OE) variant. This dose‐response relationship establishes ammonium availability as the rate‐limiting determinant for yeast proteinogenesis. Notably, the OE strain exhibited superior nitrogen assimilation capacity, achieving a maximum protein content of 59.27 ± 0.6% at 95 h, representing a significant 6.3% increase (*p *< 0.01) compared to the control strain (HTX33). Conversely, the KO mutant (Knockout) displayed impaired nitrogen conversion efficiency (max protein content: 50.66 ± 0.7%), while the RE strain (Rescue) demonstrated partial functional restoration (max protein content: 55.05 ± 0.3%) (Figure 6A). These results functionally establish *PAS_chr1‐1_0158* as both a bona fide nitrogen starvation sensor and a metabolic amplifier that orchestrates protein accumulation by artificially inducing a nitrogen‐depleted molecular signature‐thereby resolving a longstanding mechanistic hypothesis through experimental validation.

Transcriptomic analysis corroborated these phenotypic observations, positioning *PAS_chr1‐1_0158* as the master regulator of the nitrogen starvation signaling axis. This transmembrane sensor orchestrates a tripartite metabolic response: (1) enhanced ammonium fixation via glutamine synthetase upregulation (GOT1; +2.41‐fold), (2) accelerated nitrogen recycling through ω‐amidase activation (NIT2; +2.02‐fold), and (3) reinforced TCA cycle flux by malate dehydrogenase overexpression (MDH2; +7.73‐fold) (Figure 5A). Collectively, this coordinated program establishes an autotrophic nitrogen assimilation loop that significantly elevates cellular NUE beyond wild‐type levels. Crucially, its overexpression triggered metabolic reprogramming that decouples protein synthesis from canonical nitrogen catabolite repression, representing a paradigm shift in microbial nitrogen economy engineering.

To further delineate the precise regulatory hierarchy between ASNS and *PAS_chr1‐1_0158*, we performed epistasis analysis by constructing a *PAS_chr1‐1_0158* knockout strain in the ASNS tri‐copy overexpression background (HTX33‐III6‐II8‐I4‐KO). Quantitative real‐time PCR (qRT‐PCR) was employed to quantify the expression levels of core nitrogen metabolic and TCA cycle genes (NIT2, GOT1, ASNS, MDH2) in this knockout strain, compared with its parental ASNS tri‐copy overexpression strain.

The results demonstrated that knockout of *PAS_chr1‐1_0158* largely blocked the downstream transcriptomic response of key nitrogen metabolism genes, significantly reducing the transcript levels of NIT2, GOT1, and MDH2, while also causing a moderate decrease in ASNS expression itself (Figure 6B). These data confirmed that *PAS_chr1‐1_0158* does not act merely as a downstream effector of ASNS; rather, it functions in parallel with ASNS to sense and transduce common nitrogen status signals, thereby jointly driving the upregulation of nitrogen assimilation and TCA cycle genes. Notably, the observed downregulation of ASNS in the *PAS_chr1‐1_0158* knockout strain further showed a weak positive feedback loop between these two key regulators. However, the current epistasis data cannot definitively distinguish between two plausible regulatory architectures: (i) a “parallel response” model wherein *PAS_chr1‐1_0158* and ASNS function as co‐sensors that independently transduce nitrogen status signals to converge on common downstream targets, or (ii) an “upstream activation” model wherein *PAS_chr1‐1_0158* acts upstream of ASNS to modulate its expression level, with the observed ASNS downregulation in the KO strain reflecting hierarchical dependency rather than feedback regulation. Discriminating between these scenarios would require additional experiments, such as epistasis analysis in a *PAS_chr1‐1_0158* overexpression background combined with ASNS knockout, or promoter‐reporter assays to directly assess whether *PAS_chr1‐1_0158* modulates ASNS transcriptional activity. Collectively, these findings validated *PAS_chr1‐1_0158* as a critical node in the nitrogen signaling circuit activated by ASNS overexpression, underscoring its indispensability for the full activation of downstream nitrogen–carbon synergistic metabolism.

## 3 Discussion

Microorganism‐derived SCP represents a transformative solution to the global protein crisis, offering sustainable alternatives that circumvent the environmental burdens of conventional livestock systems. Among emerging platforms, the methylotrophic yeast *P. pastoris* stands out as a particularly promising candidate for methanol‐based biosynthesis, combining rapid growth kinetics (doubling time < 2 h), minimal spatial footprint (100‐fold higher volumetric productivity than soybean cultivation), and climate‐resilient cultivation in controlled bioreactors [24, 25, 26]. This approach achieves enhanced sustainability through integration with emerging C1 circular economy systems‐methanol can be synthesised via photocatalytic or electrochemical CO_2_ reduction technologies, enabling near‐zero carbon footprint SCP production when coupled with renewable energy inputs [27]. Recent advancements in green methanol synthesis, particularly through renewable electricity‐driven carbon capture and conversion systems [11], have dramatically improved cost‐effectiveness, positioning methanol as a viable substrate for industrial‐scale biomanufacturing. However, the inherent metabolic dichotomy between methanol‐derived carbon flux and nitrogen assimilation efficiency creates fundamental constraints on proteogenic capacity in native *P. pastoris*. Overcoming these limitations through targeted metabolic engineering represents a crucial step toward economically viable SCP production systems, with broader implications for sustainable food security and carbon‐neutral biomanufacturing.

Traditional SCP optimization strategies have been constrained by a carbon‐centric paradigm, focusing predominantly on enhancing methanol assimilation pathways or alleviating formaldehyde toxicity [12, 28]. Our study fundamentally shifted this paradigm by pioneering a cross‐kingdom metabolic engineering strategy. This innovative approach strategically focuses on intensifying nitrogen metabolism to establish synergistic coupling with carbon flux. Molecular evolutionary analyses have confirmed the profound functional conservation of the ASNS gene across eukaryotes. Further correlation analyses across multiple yeast strains have revealed a significant positive correlation between ASNS expression levels and intracellular protein accumulation, thereby establishing ASNS as a core regulatory hub for yeast protein biosynthetic capacity. Despite phenotypic heterogeneity observed among strains with equivalent ASNS expression levels, the underlying mechanisms can be traced back to three fundamental biological processes. First, phenotypic heterogeneity among strains [29] influenced the state of the transcriptional regulatory network controlling ASNS expression, thereby leading to distinct baseline levels of protein synthesis. Second, strain‐specific variations in carbon‐nitrogen metabolic coupling efficiency [30] alter precursor flux partitioning between biomass synthesis and alternative metabolic sinks. Certain strains exhibited more efficient metabolic pathways during carbon‐to‐nitrogen source conversion, directing greater precursor flux toward protein synthesis, while others preferentially diverted precursors into alternative metabolic pathways, thereby generating disparities in protein production. Finally, distinct environmental signal transduction structures confer varying sensitivities, which are manifested as specific regulatory responses of microorganisms to environmental fluctuations with distinct temporal characteristics [31]. When exposed to the same nitrogen environment, different strains differentially regulate ASNS expression through their unique signal transduction pathways, thereby achieving strain‐specific adaptive adjustments in protein synthesis. While the aforementioned multi‐tiered regulatory mechanisms mediate phenotypic variation, the global strong correlation between ASNS expression and protein yield still highlights its irreplaceability in reprogramming nitrogen resource allocation and driving the protein synthesis network. This finding provides a theoretical anchor for cross‐species metabolic engineering.

By overexpressing the maize‐homologous ASNS gene, evolutionarily optimised from plants to the methylotrophic yeast *P. pastoris*, we have created a novel metabolic virtuous cycle that dramatically enhances methanol conversion efficiency and SCP yield. This strategy directly leverages conserved regulatory mechanisms across biological kingdoms, particularly the principle that enhancing nitrogen assimilation efficiency in plants [16, 21, 22] intrinsically optimises precursor supply for central carbon metabolism. Thus, our work not only demonstrates the first successful implementation of plant‐derived nitrogen optimization machinery in a microbial SCP platform but also establishes a new frontier in sustainable protein production: bridging the gap between photosynthetic nitrogen strategies and industrial microbial cultivation through synthetic biology.

Transcriptomic analysis revealed that ASNS overexpression triggered a nitrogen‐centric regulatory cascade, prominently activating the previously uncharacterised gene *PAS_chr1‐1_0158*, annotated as a putative nitrogen starvation‐responsive transmembrane sensor but never functionally validated. This genetic perturbation accelerated nitrogen cycling efficiency by 6.3% (*p *< 0.01) while optimizing nitrogen source utilization through dynamic regulation of glutamine/glutamate metabolic nodes. Our findings align with established biochemical principles of microbial metabolism, where protein biosynthesis operates under dual carbon‐nitrogen metabolic constraints, well‐documented in prokaryotes [32]. The observed coupling revealed an evolutionarily conserved paradigm: augmenting either carbon or nitrogen flux creates a feedforward loop that reciprocally enhances utilization efficiency of the complementary nutrient source [33]. Consistent with this, ASNS overexpression also transcriptionally enhanced the TCA cycle, corroborated by increased methanol consumption, indicating that improved nitrogen conversion efficiency boosted carbon source utilization and contributed to SCP accumulation in *P. pastoris*. These results demonstrate ASNS's crucial role in orchestrating carbon‐nitrogen synergy by modulating cellular nitrogen metabolism. Critically, we performed direct functional validation studies on *PAS_chr1‐1_0158*, which confirmed its essential role in yeast's response to environmental nitrogen levels and the regulation of intracellular crude protein accumulation. For the first time, we experimentally demonstrated that overexpression of *PAS_chr1‐1_0158* significantly enhanced protein content in yeast biomass, particularly under sufficient nitrogen supply. Conversely, targeted knockout of *PAS_chr1‐1_0158* impaired nitrogen utilization efficiency and suppressed protein synthesis. The phenotypic recovery observed upon genetic rescue in the knockout strain definitively substantiates the specific and essential functional role of *PAS_chr1‐1_0158* in this biological process. This discovery‐identifying *PAS_chr1‐1_0158*’s dual role as both a nitrogen sensor and a proteo‐geneis amplifier‐provides a revolutionary engineering target for next‐generation SCP systems, enabling precise tuning of microbial protein synthesis under dynamic industrial conditions. Targeted metabolomics further confirmed that this regulatory cascade is initiated by ASNS‐induced nitrogen status imbalance. The elevated Gln/Glu ratio provided a plausible molecular signature for *PAS_chr1‐1_0158* sensing. While the exact metabolite ligand of *PAS_chr1‐1_0158* requires future validation, this data establish that ASNS overexpression disrupted the steady‐state nitrogen equilibrium, triggering a compensatory nitrogen assimilation program that ultimately enhanced protein production.

The nutritional value of SCP derived from *P. pastoris* hinges on its crude protein content and amino acid composition [26]. A pivotal challenge for advancing sustainable protein production lies in boosting the efficiency of converting inorganic nitrogen sources into organically bound nitrogen within the microbial biomass. Addressing the under‐utilised protein synthesis potential in yeast under standard conditions [34], this study employed an evolution‐inspired strategy: heterologous overexpression of maize‐homologous ASNS, confirming the structural and functional conservation of this enzyme across eukaryotes. Genome‐scale metabolic modeling predicted that ASNS overexpression would induce broad metabolic flux redistributions, a prediction empirically confirmed through experimental characterization. While the metabolic remodeling demonstrated exceptional coordination featuring intricate cross‐regulatory interactions between previously distinct modules, we acknowledge that the aspartate‐TCA cycle axis remains partially inferred from computational predictions. Direct experimental validation through targeted quantification of TCA cycle intermediates and isotope‐tracing‐based flux analysis constitutes an important direction for future investigation. The current modeling framework provides a robust hypothesis‐generating foundation; however, empirical flux measurements would be required to definitively establish the causal link between ASNS‐mediated nitrogen assimilation and carbon skeleton routing into the TCA cycle. These unresolved mechanistic layers do not diminish the established phenotypic outcomes of ASNS engineering but highlight priority targets for deepening our understanding of carbon‐nitrogen metabolic synergy. The resulting metabolic remodeling demonstrated exceptional coordination, featuring intricate cross‐regulatory interactions between previously distinct modules. Critically, this intervention achieved a significant 11.4% enhancement in SCP biosynthetic efficiency (62.07% vs 55.74% crude protein in wild‐type), surpassing conventional protein sources like meat (21.2%) and soybean (35%), and outperforming conventional yeast SCP (53%–56%) [26, 35, 36, 37]. The engineered strain delivered a nutritionally superior profile with 47.86% total amino acids, including 41.04% essential amino acids and a high 8.05% branched‐chain amino acids, while being a rich source of typically limiting amino acids (lysine and methionine) [26, 36, 38].BCAAs serve as important building blocks in the body, promoting glucose uptake and increasing ATP production [39]. Furthermore, BCAAs play a role in regulating body lipid metabolism, protein synthesis, and the immune response [40, 41]. These findings not only elucidate the molecular basis of carbon‐nitrogen coupling in methylotrophic yeasts but also establish a synthetic biology paradigm for overcoming inherent metabolic bottlenecks using conserved eukaryotic regulatory elements. Compared with plant‐based protein production systems constrained by photosynthetic inefficiency and high resource consumption, this strategy demonstrates spatiotemporal‐unrestricted sustainable protein production potential. In the future, optimization strategies, such as the meticulous fine‐tuning of specific amino acid ratios, can further enhance the nutritional quality. This, in turn, will act as a powerful driving force for the development of next‐generation microbial cell factories dedicated to the high‐value and genuinely sustainable biomanufacturing of SCP.

Transcriptomic profiling and targeted quantification of glutamine and glutamate have substantiated the ASNS‐dependent coordination of carbon and nitrogen metabolism in *P. pastoris*. However, the functional linkage between the aspartate metabolic branch and the TCA cycle is presently inferred solely from genome‐scale modeling and awaits empirical verification. The elevated Gln/Glu ratio in the triple‐copy ASNS strain serves as a molecular marker for perturbed nitrogen balance. To validate the predicted rerouting of carbon flux, subsequent studies will employ ^13^C‐metabolic flux analysis combined with targeted LC‐MS/MS metabolomics to quantify metabolites within the aspartate‐oxaloacetate‐malate module and core TCA cycle intermediates. Notably, this study has several inherent limitations. While the physiological function of *PAS_chr1‐1_0158* as a nitrogen‐sensing regulator has been experimentally characterized, its biochemical properties are deduced exclusively from bioinformatic predictions and homologous sequence annotation. In vitro biochemical assays are therefore necessary to verify its putative ammonia transport or signal recognition activity. Additionally, the existing genetic and transcriptomic evidence cannot fully resolve the upstream–downstream regulatory relationship between *PAS_chr1‐1_0158* and ASNS. Critically, these outstanding questions do not undermine our central conclusion that these two factors cooperatively enhance nitrogen utilization and carbon‐nitrogen metabolic integration.

In‐depth analysis of *P. pastoris* strains has unveiled crucial regulatory nodes in nitrogen metabolism, offering novel and profound insights into the microbial carbon‐nitrogen coupling mechanism. This research pioneers a transformative paradigm in sustainable biomanufacturing by establishing cross‐kingdom metabolic engineering as a universal strategy for nutrient optimization. By successfully transplanting plant‐evolved nitrogen assimilation mechanisms into microbial systems, we have fundamentally altered the engineering logic of SCP production from isolated pathway modifications to holistic network evolution. This strategy, by decisively strengthening carbon‐nitrogen synergy to drive unprecedented conversion efficiency, unlocks not merely new avenues but a scalable and robust platform for high‐performance SCP production from *P. pastoris*. Crucially, it provides the essential scientific and technological bedrock required to propel this microbial platform toward industrial‐scale implementation. Ultimately, this work transcends the advancement of a single microbial system; it delineates a powerful synthetic biology blueprint with profound implications for revolutionizing global protein production. It offers a tangible and sustainable pathway toward meeting the planet's escalating protein demands while concurrently mitigating the severe environmental burdens associated with conventional agriculture, thereby contributing significantly to a more secure and resilient food future.

## 4 Materials and Methods

### 4.1 Phylogenetic Tree

According to the existing research reports, the ASNS amino acid sequences of 31 different species were obtained from the NCBI database. The Clustal Omega algorithm is used for comparison. The MEGA 7.0 Maximum Likelihood Estimation (MLE) was used to construct the phylogenetic tree, and the step value was set to 1000.

### 4.2 Establishment of Genome‐Scale Metabolic Network Model

Flux scanning based on enforced objective flux is a constraint‐based computational method implemented within genome‐scale metabolic network models to systematically identify gene amplification targets for enhanced metabolite production. The procedure initiates with constraint‐based flux analysis using linear programming to calculate initial flux distributions (vjinitial) under biomass maximization objectives while satisfying stoichiometric balances, thermodynamic constraints, and cellular maintenance requirements (e.g., vATPM ≥vatp_maint). Subsequently, the theoretical maximum product flux (vproductmax) is determined by optimizing the model specifically for maximal target metabolite formation. The core scanning procedure then imposes incrementally enforced product flux constraints (vproductenforced = vproductinitial+nk(vproductmax − vproductinitial) for k = 1, 2, …, n−1) during repeated flux balance analyses where biomass maximization remains the primary objective. Throughout these constrained optimizations, metabolic fluxes are systematically monitored to identify reactions exhibiting both (1) monotonically increasing absolute flux magnitudes with increasing product flux enforcement (∣vj∣max>∣vjinitial∣), and (2) invariant reaction directionality across all enforcement steps (vjmax ×vjmin ≥0). This dual‐criterion approach enables robust identification of amplification targets by detecting flux trends rather than relying on single‐point flux comparisons, effectively overcoming limitations of intuitive target selection in complex metabolic networks [42].

### 4.3 Strains, Media, and Cultivation

All strains and plasmids used in this study are listed in Table 1. The plasmid pPICZ‐Cas9‐gGUT1 [23] was a generous gift from our laboratory. Other *P. pastoris* strains and plasmids were obtained from Dalian Institute of Chemical Physics (DICP), Chinese Academy of Sciences, and were either preserved in our laboratory or constructed in this study. *E. coli* DH5α, used as a host for plasmid construction, was cultured in LB medium containing 5 g/L yeast extract, 10 g/L tryptone, 10 g/L NaCl, and 50 µg/mL zeocin at 37°C with 220 rpm shaking. Unless otherwise specified, *P. pastoris* strains were cultured in YPD medium supplemented with 100 mg/L zeocin for selection of transformants. Cells were cultured in Delft basic salt medium (containing 7.5 g / L (NH_4_)_2_SO_4_,14.4 g / L KH_2_PO_4_,0.5 g / L MgSO_4_ 7H_2_O, 1 m L/L vitamin solution and 2 m L/L PTM1 trace salt solution) with methanol as the sole carbon source [43].

**TABLE 1:** Strains and plasmids used in this study.

Strains and Plasmids	Relevant characteristics	Sources
Strains		
HTX33	Derived from X‐33 through ALE	Lab stock
HTX33‐III6	Derived from HTX‐33, PNSIII‐6: GAP‐ASNS‐A0X	This study
HTX33‐III6‐II8	Derived from HTX33‐III6, PNSIII6‐II8: GAP‐ASNS‐A0X	This study
HTX33‐III6‐II8‐I4	Derived from HTX33‐III6‐II8, PNSIII6‐II8‐I4: GAP‐ASNS‐A0X	This study
KO	Derived from HTX‐33, PNS0158: TEF1‐ASNS‐CYC1	This study
RE	Derived fromKO, PNS0158: GAP‐0158‐ A0X	This study
OE	Derived from HTX33, PNSI2: GAP‐0158‐A0X	This study
E. coli		
DH5a	F−, φ80lacZ ΔM15, Δ(lacZYA‐argF)U169, deoR, recA1, endA1, hsdR17(rk−, mk+), phoA, supE44, λ−, thi‐1, gyrA96, relA1	Lab stock
Plasmids		
pPICZ‐Cas9‐gGUT1	ori, Amp, Zeocin, TDAS1‐Cas9‐PHTX1‐GUT1‐gRNA2‐TAOX	[23]
pPICZ‐Cas9‐gPNSIII‐6	ori, Amp, Zeocin, TDAS1‐Cas9‐PHTX1‐PNSIII‐6‐gRNA‐TAOX	This study
pPICZ‐Cas9‐gPNSII‐8	ori, Amp, Zeocin, TDAS1‐Cas9‐PHTX1‐PNSII‐8‐gRNA‐TAOX	This study
pPICZ‐Cas9‐gPNSI‐4	ori, Amp, Zeocin, TDAS1‐Cas9‐PHTX1‐PNSI‐4‐gRNA‐TAOX	This study
pPICZ‐Cas9‐gPNSI‐2	ori, Amp, Zeocin, TDAS1‐Cas9‐PHTX1‐PNSI‐2‐gRNA‐TAOX	This study
pPICZ‐Cas9‐gPNS0158	ori, Amp, Zeocin, TDAS1‐Cas9‐PHTX1‐PNS0158‐gRNA‐TAOX	This study
pPICZ‐Cas9‐gPNS0158	ori, Kan, G418, TDAS1‐Cas9‐PHTX1‐PNS0158‐gRNA‐TAOX	This study

### 4.4 Plasmids and Strains Construction

To construct the ASNS‐donor DNA, the upstream region, promoter region, ASNS region, terminator region, and downstream region were amplified from the *P. pastoris* genome using primers PNSIII‐6‐up‐F/PNSIII‐6‐up‐R, PNSIII‐6‐down‐F/PNSIII‐6‐down‐R, PNSII‐8‐up‐F/PNSII‐8‐up‐R, PNSII‐8‐down‐F/PNSII‐8‐down‐R, PNSI‐4‐up‐F/PNSI‐4‐up‐R, PNSI‐4‐down‐F/PNSI‐4‐down‐R, ASNS‐F/ASNS‐R, GAP‐F/GAP‐R, and AOX1 terminator‐F/AOX1 terminator‐R, respectively; these five fragments were then fused by fusion PCR to generate the ASNS‐donor DNA cassette, and the ASNS expression cassettes for PNSIII‐6, PNSII‐8, and PNSI‐4 loci were constructed separately. Similarly, for the construction of the 0158‐donor DNA and zeocin‐donor DNA, the upstream region, promoter region, 0158 region, zeocin region, terminator region, and downstream region were amplified from the *P. pastoris* genome using primers PNSI‐2‐up‐F/PNSI‐2‐up‐R, PNSI‐2‐down‐F/PNSI‐2‐down‐R, PNS0158‐up‐F/PNS0158‐up‐R, PNS0158‐down‐F/PNS0158‐down‐R, 0158‐F/0158‐R, zeocin‐F/zeocin‐R, GAP‐F/GAP‐R and AOX1 terminator‐F/AOX1 terminator‐R, respectively, and fused by fusion PCR to generate the 0158‐donor DNA cassette and the zeocin‐donor DNA cassette; the 0158 expression cassettes and zeocin expression cassettes for PN0158 loci, as well as the 0158 expression cassettes for PNI‐2 loci, were constructed separately. Finally, the gRNA expression plasmid and the corresponding donor DNA were co‐transformed into *P. pastoris* competent cells, which were grown on YPD plates containing 100 µg/mL zeocin for 2 days, and the transformants were verified by PCR and further confirmed by DNA sequencing. All primers used for strain and plasmid construction are listed in Table S1.

### 4.5 Validation of Genes by Real‐Time Quantitative Polymerase Chain Reaction (RT‐qPCR)

The transcription level of ASNS was determined by RT‐qPCR. Total RNA was extracted from exponentially growing *P. pastoris* HTX33, HTX33‐III6, HTX33‐III6‐II8, and HTX33‐III6‐II8‐I4 and different yeast strains (Table S3) using TransZol Up Plus RNA Kit (TransGen, Beijing, China). The quality and concentration of the extracted RNA were then measured using a Nanodrop 2000c (Thermo). cDNA was synthesized from 200 ng RNA using Quant Reverse Transcriptase and random primers (TransGen, Beijing, China). RT‐qPCR was performed on a LightCycler 480 II (Roche, Basel, Switzerland) using Real Master Mix (SYBR Green) and specific primers (Table S4), following the following cycling conditions: 50°C for 5 min, 94°C for 30 s, 94°C for 5 s, and 60°C for 30 s, for 45 cycles. The ACT1 gene was used as a normalization reference gene, and all reactions were performed in triplicate. The transcriptional level of the target gene (ASNS) was calculated using the 2^−ΔCt^ method. The results are expressed as the relative expression levels to the endogenous control gene (ACT1), denoted as 2^−ΔCt^ [44].

### 4.6 Fed‐Batch Fermentation

After overnight culture in YPD medium at 30°C, cells were inoculated (8%, v/v) into a 5 L bioreactor containing 3 L of basal salts medium (BSM) supplemented with 4.35 mL/L PTM1 trace salts. The temperature and pH were set at 30°C and 6.0, respectively. The initial agitation speed was set at 100 rpm and increased to a maximum of 300 rpm based on the air flow rate. The initial air flow rate was set at 0.55 vvm and increased to a maximum of 1.5 vvm.

The fermentation process was divided into three phases. In phase I, the focus was on *P. pastoris* cell growth, and the dissolved oxygen (DO) level was maintained above 30% of air saturation by adjusting the agitation speed (200–800 rpm). In the second phase, a 50% (w/w) glycerol solution (containing 12 mL/L PTM1 solution) was continuously fed into the reactor to maintain the DO level above 30%. In the third phase, methanol was pumped into the vessel, and the residual methanol was monitored and controlled throughout the fermentation process [45].

### 4.7 Total Protein Determination and Amino Acid Analysis

The cell pellet from 5 mL of culture was washed three times with sterile water and resuspended in 1 mL of sterile water. The centrifuge tubes containing the cell pellet were dried to a constant weight at 105°C. The nitrogen content of the homogenized residue was analyzed using the Kjeldahl method [46]. The nitrogen content was converted to protein content using a conversion factor of 6.25.

Amino acid composition was analysed by pre‐column derivatization with phenyl isothiocyanate (PITC) followed by reversed‐phase HPLC. Samples were processed using the established hydrolysis protocol: 40 mg of *P. pastoris* biomass was mixed with 15 mL 6 M HCl (1:1 v/v hydrochloric acid/water) in hydrolysis tubes, vacuum‐sealed, and hydrolyzed at 140°C. for 9 h. Hydrolysates were diluted to 50 mL with deionized water. For derivatization, 1 mL was placed in a 60°C metal bath for drying and reacted with PITC reagent at room temperature for 1 h. Analysis of the amino acid content by phenyl isothiocyanate column prederivative method using the Diamonsil AAA amino acid analysis column (250 × 4.6 mm, 5 µm, Agilent, USA) [47].

### 4.8 Transcriptome Analysis

Cultivate the control *P. pastoris* HTX‐33 and the overexpression *P. pastoris* ASNS strain in a fermentation tank at 30°C. Upon reaching the exponential growth phase, total RNA was extracted from both strains using TRIzol (Invitrogen) according to the manufacturer's instructions. Only RNA samples with an integrity score exceeding 6.5, as determined by Agilent 2100 Nano (Agilent Technologies), were used for library construction and sequencing. Complementary DNA libraries were prepared and sequenced using the Illumina HiSeq 2000 platform at Meiji Biotechnology (Shanghai) Co., Ltd. Transcript abundance was quantified as transcripts per million reads (TPM) using RESM software (https://deweylab.github.io/RSEM/). The differential expression between the two samples was analyzed by the DEGseq (https://bioinfo.au.tsinghua.edu.cn/ software/degseq), and genes with |log_2_FC|≥1 and *p *< 0.05 were identified as significantly differentially expressed genes (DEGs). The Kyoto Encyclopedia of Genes and Genomes (KEGG) enrichment analysis was performed using the KOBAS (https://kobas.cbi.pku.edu.cn/home. do).

### 4.9 Quantification of Intracellular Glutamate and Glutamine in *P. pastoris*

Concentrations of glutamate and glutamine were determined via high‐performance liquid chromatography. Chromatographic separation was performed on NH_2_ Column (Agilent, USA). The mobile phase consisted of 50 mM KH_2_PO_4_ solution and acetonitrile in a ratio of 70:30 (v/v). The flow rate was maintained at 1.0 mL/min, and the column temperature was set to 30°C. Detection was carried out using a fluorescence detector with wavelengths set at 215 nm. Three independent biological replicates were performed for each strain.

## Author Contributions

Carried out the experimental work, Yuanyuan Du, Le Gao; Prepared the original draft, Yuanyuan Du, Le Gao; Analyzed the results, Yuanyuan Du, Changyu Pi, Kai Hong, Le Gao; Review and editing, Le Gao, Xin Wu; Funding acquisition, Xin Wu. All authors have read and agreed to the published version of the manuscript.

## Conflicts of Interest

The authors declare no conflicts of interest.

## Supporting information




**Supporting File 1**: advs76229‐sup‐0001‐SuppMat.pdf.

**Supporting File 2**: advs76229‐sup‐0002‐Fig. S3.docx.

**Supporting File 3**: advs76229‐sup‐0003‐Table S1.pdf.

**Supporting File 4**: advs76229‐sup‐0004‐Table S2.pdf.

**Supporting File 5**: advs76229‐sup‐0005‐Table S3.pdf.

**Supporting File 6**: advs76229‐sup‐0006‐Table S4.pdf.

## Data Availability

The data that support the findings of this study are available from the corresponding author upon reasonable request.
