# Supplementary material for: Cross Kingdom Metabolic Engineering Paradigm Elevating Sustainable Protein Production
Source: Adv Sci (Weinh). 2026 Jun 23;13(52):e17703. doi: 10.1002/advs.202517703 (PMC13336901; doi:10.1002/advs.202517703)

Supplementary Figure 1 ASNS protein sequence alignment results of different. species. Each row represents a homologous protein sequence of a species, and each column represents an amino acid position. The conservation of amino acid sites is marked by color: red indicates complete conservation (amino acids at this site are consistent in all sequences), dark blue indicates high conservation (amino acids at this site belong to the same class in most sequences, for example, are polar amino acids), and light blue indicates relative conservation (amino acids at this site have similar properties in some sequences).

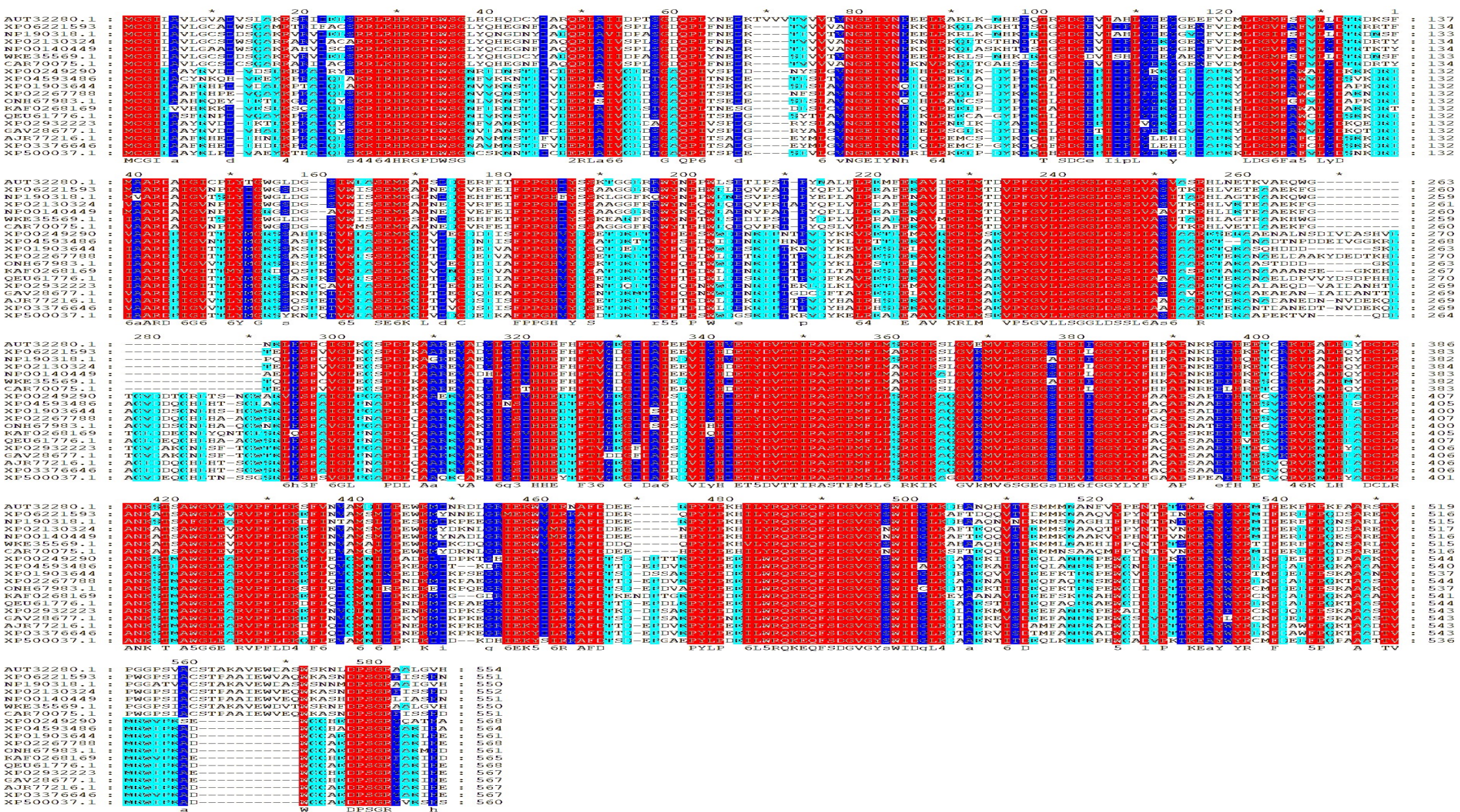

Supplementary Figure 2 Domain architecture of ASNS genes.

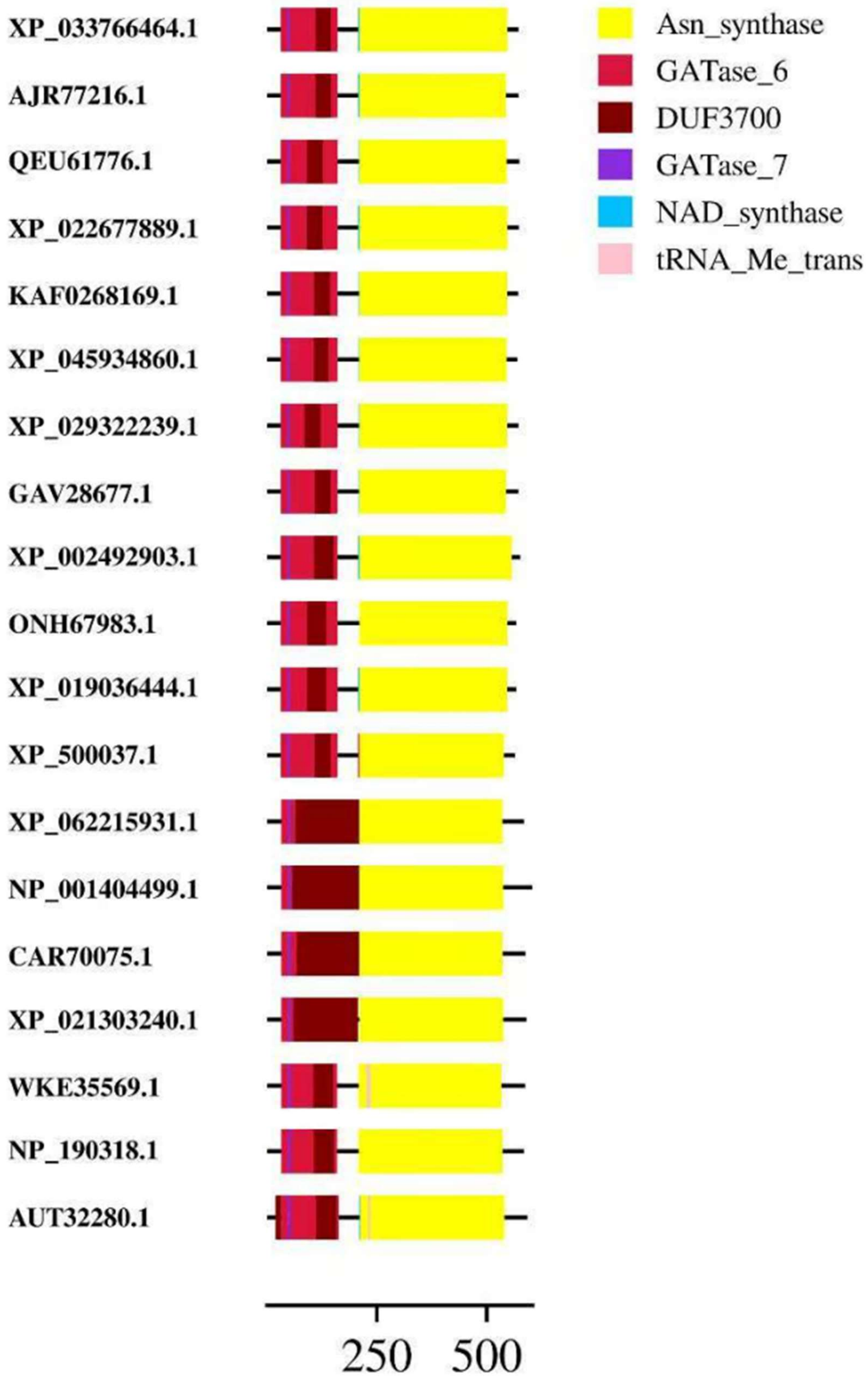

Supplement: Supplementary file 2 [file ADVS-13-e17703-s002.pdf]
