# Supplementary material for: Cross Kingdom Metabolic Engineering Paradigm Elevating Sustainable Protein Production
Source: Adv Sci (Weinh). 2026 Jun 23;13(52):e17703. doi: 10.1002/advs.202517703 (PMC13336901; doi:10.1002/advs.202517703)
Supplement: Supplementary file 3 [file ADVS-13-e17703-s004.pdf]

Table S2. Total enrichment results of biological pathways KEGG

Pathway ID	Description	First Category	Second Category	Gene_ids	Gene_names
map04146	Peroxisome	Cellular Processes	Transport and catabolism	gene-PAS_chr3_0822|gene-PAS_chr1-4_0133|gene-PAS_chr2-1_0504|gene-PAS_chr2-1_0785|gene-PAS_chr1-4_0663|gene-PAS_chr3_0043|gene-PAS_chr1-4_0074|gene-PAS_chr3_0069|gene-PAS_chr2-2_0207|gene-PAS_chr2-1_0580|gene-PAS_chr3_1045|gene-PAS_chr2-2_0267|gene-PAS_chr4_0759|gene-PAS_chr1-1_0352|gene-PAS_chr1-4_0538|gene-PAS_chr4_0794|gene-PAS_FragB_0025|gene-PAS_FragB_0022|gene-PAS_chr4_0352|gene-PAS_chr4_0416|gene-PAS_chr3_0761|gene-PAS_chr3_1073|gene-PAS_chr2-2_0186|gene-PAS_chr2-2_0273|gene-PAS_chr1-4_0433|gene-PAS_chr2-1_0715|gene-PAS_chr2-2_0272	PAS_chr3_0822;PAS_chr1-4_0133;PAS_chr2-1_0504;PAS_chr2-1_0785;PAS_chr1-4_0663;PAS_chr3_0043;PAS_chr1-4_0074;PAS_chr3_0069;PAS_chr2-2_0207;PAS_chr2-1_0580;PAS_chr3_1045;PAS_chr2-2_0267;PAS_chr4_0759;PAS_chr1-1_0352;PAS_chr1-4_0538;PAS_chr4_0794;PAS_FragB_0025;PAS_FragB_0022;PAS_chr4_0352;PAS_chr4_0416;PAS_chr3_0761;PAS_chr3_1073;PAS_chr2-2_0186;PAS_chr2-2_0273;PAS_chr1-4_0433;PAS_chr2-1_0715;PAS_chr2-2_0272
map00010	Glycolysis / Gluconeogenesis	Metabolism	Carbohydrate metabolism	gene-PAS_chr3_0826|gene-PAS_chr1-4_0264|gene-PAS_chr2-1_0402|gene-PAS_chr2-2_0294|gene-PAS_chr3_0188|gene-PAS_chr3_0868|gene-PAS_chr1-4_0561|gene-PAS_chr2-1_0767|gene-PAS_chr1-4_0447|gene-PAS_chr2-1_0769|gene-PAS_chr3_0951|gene-PAS_chr1-4_0047|gene-PAS_chr2-1_0453|gene-PAS_chr1-4_0593|gene-PAS_FragB_0061|gene-PAS_chr1-4_0292|gene-PAS_chr2-1_0437|gene-PAS_chr4_0043|gene-PAS_chr3_0693|gene-PAS_chr4_0624|gene-PAS_chr3_0456|gene-PAS_chr2-1_0853|gene-PAS_chr1-1_0072|gene-PAS_chr3_0082|gene-PAS_chr1-1_0050	PAS_chr3_0826;PAS_chr1-4_0264;PAS_chr2-1_0402;PAS_chr2-2_0294;PAS_chr3_0188;PAS_chr3_0868;PAS_chr1-4_0561;PAS_chr2-1_0767;PAS_chr1-4_0447;PAS_chr2-1_0769;PAS_chr3_0951;PAS_chr1-4_0047;PAS_chr2-1_0453;PAS_chr1-4_0593;PAS_FragB_0061;PAS_chr1-4_0292;PAS_chr2-1_0437;PAS_chr4_0043;PAS_chr3_0693;PAS_chr4_0624;PAS_chr3_0456;PAS_chr2-1_0853;PAS_chr1-1_0072;PAS_chr3_0082;PAS_chr1-1_0050
map00680	Methane metabolism	Metabolism	Energy metabolism	gene-PAS_chr3_0826|gene-PAS_chr3_0834|gene-PAS_chr4_0416|gene-PAS_chr4_0821|gene-PAS_chr3_0832|gene-PAS_chr4_0285|gene-PAS_chr4_0587|gene-PAS_chr4_0152|gene-PAS_chr1-4_0047|gene-PAS_chr2-1_0402|gene-PAS_chr3_0868|gene-PAS_chr1-1_0072|gene-PAS_chr3_0082|gene-PAS_chr3_0693|gene-PAS_chr2-1_0767	PAS_chr3_0826;PAS_chr3_0834;PAS_chr4_0416;PAS_chr4_0821;PAS_chr3_0832;PAS_chr4_0285;PAS_chr4_0587;PAS_chr4_0152;PAS_chr1-4_0047;PAS_chr2-1_0402;PAS_chr3_0868;PAS_chr1-1_0072;PAS_chr3_0082;PAS_chr3_0693;PAS_chr2-1_0767
map00051	Fructose and mannose metabolism	Metabolism	Carbohydrate metabolism	gene-PAS_chr1-1_0356|gene-PAS_chr2-1_0870|gene-PAS_chr4_0341|gene-PAS_chr1-4_0075|gene-PAS_chr4_0266|gene-PAS_chr2-1_0110|gene-PAS_chr1-1_0072|gene-PAS_chr4_0624|gene-PAS_chr4_0577|gene-PAS_chr2-1_0402|gene-PAS_chr3_0868|gene-PAS_chr1-4_0561|gene-PAS_chr1-4_0047|gene-PAS_chr1-4_0447|gene-PAS_chr4_0339|gene-PAS_chr3_0951	PAS_chr1-1_0356;PAS_chr2-1_0870;PAS_chr4_0341;PAS_chr1-4_0075;PAS_chr4_0266;PAS_chr2-1_0110;PAS_chr1-1_0072;PAS_chr4_0624;PAS_chr4_0577;PAS_chr2-1_0402;PAS_chr3_0868;PAS_chr1-4_0561;PAS_chr1-4_0047;PAS_chr1-4_0447;PAS_chr4_0339;PAS_chr3_0951
map00071	Fatty acid degradation	Metabolism	Lipid metabolism	gene-PAS_chr4_0352|gene-PAS_chr1-4_0538|gene-PAS_chr1-4_0304|gene-PAS_chr2-1_0453|gene-PAS_chr2-1_0853|gene-PAS_chr2-1_0785|gene-PAS_chr1-4_0663|gene-PAS_chr2-2_0267|gene-PAS_chr4_0043	PAS_chr4_0352;PAS_chr1-4_0538;PAS_chr1-4_0304;PAS_chr2-1_0453;PAS_chr2-1_0853;PAS_chr2-1_0785;PAS_chr1-4_0663;PAS_chr2-2_0267;PAS_chr4_0043
map00920	Sulfur metabolism	Metabolism	Energy metabolism	gene-PAS_chr3_1084|gene-PAS_chr3_0667|gene-PAS_chr4_0369|gene-PAS_chr4_0330|gene-PAS_chr2-1_0547|gene-PAS_chr2-1_0862|gene-PAS_chr1-4_0253|gene-PAS_chr4_0865	PAS_chr3_1084;PAS_chr3_0667;PAS_chr4_0369;PAS_chr4_0330;PAS_chr2-1_0547;PAS_chr2-1_0862;PAS_chr1-4_0253;PAS_chr4_0865
map00061	Fatty acid biosynthesis	Metabolism	Lipid metabolism	gene-PAS_chr4_0352|gene-PAS_chr1-4_0295|gene-PAS_chr1-1_0008|gene-PAS_chr1-4_0249|gene-PAS_chr2-1_0785|gene-PAS_chr2-1_0856|gene-PAS_chr4_0511	PAS_chr4_0352;PAS_chr1-4_0295;PAS_chr1-1_0008;PAS_chr1-4_0249;PAS_chr2-1_0785;PAS_chr2-1_0856;PAS_chr4_0511
map00052	Galactose metabolism	Metabolism	Carbohydrate metabolism	gene-PAS_chr1-4_0264|gene-PAS_chr4_0624|gene-PAS_chr1-4_0047|gene-PAS_chr1-3_0122|gene-PAS_chr2-1_0402|gene-PAS_chr1-4_0561|gene-PAS_chr1-4_0447	PAS_chr1-4_0264;PAS_chr4_0624;PAS_chr1-4_0047;PAS_chr1-3_0122;PAS_chr2-1_0402;PAS_chr1-4_0561;PAS_chr1-4_0447
map00780	Biotin metabolism	Metabolism	Metabolism of cofactors and vitamins	gene-PAS_chr1-3_0052|gene-PAS_chr3_0569|gene-PAS_chr2-1_0856|gene-PAS_chr4_0511	PAS_chr1-3_0052;PAS_chr3_0569;PAS_chr2-1_0856;PAS_chr4_0511
map00500	Starch and sucrose metabolism	Metabolism	Carbohydrate metabolism	gene-PAS_chr4_0579|gene-PAS_chr2-1_0263|gene-PAS_chr4_0692|gene-PAS_chr1-4_0264|gene-PAS_chr4_0624|gene-PAS_chr1-3_0122|gene-PAS_chr3_0456|gene-PAS_chr1-4_0561|gene-PAS_chr1-3_0225|gene-PAS_chr4_0847|gene-PAS_chr1-4_0447|gene-PAS_chr4_0013	PAS_chr4_0579;PAS_chr2-1_0263;PAS_chr4_0692;PAS_chr1-4_0264;PAS_chr4_0624;PAS_chr1-3_0122;PAS_chr3_0456;PAS_chr1-4_0561;PAS_chr1-3_0225;PAS_chr4_0847;PAS_chr1-4_0447;PAS_chr4_0013
map00410	beta-Alanine metabolism	Metabolism	Metabolism of other amino acids	gene-PAS_chr2-1_0453|gene-PAS_chr2-1_0343|gene-PAS_chr4_0823|gene-PAS_chr1-4_0538|gene-PAS_chr1-4_0198|gene-PAS_chr3_0937|gene-PAS_chr3_0922|gene-PAS_chr4_0043|gene-PAS_chr2-1_0776|gene-PAS_chr2-1_0853	PAS_chr2-1_0453;PAS_chr2-1_0343;PAS_chr4_0823;PAS_chr1-4_0538;PAS_chr1-4_0198;PAS_chr3_0937;PAS_chr3_0922;PAS_chr4_0043;PAS_chr2-1_0776;PAS_chr2-1_0853
map00340	Histidine metabolism	Metabolism	Amino acid metabolism	gene-PAS_chr4_0343|gene-PAS_chr4_0703|gene-PAS_chr1-4_0160|gene-PAS_chr2-1_0453|gene-PAS_chr2-1_0853|gene-PAS_chr1-4_0234|gene-PAS_chr4_0043|gene-PAS_chr2-1_0684	PAS_chr4_0343;PAS_chr4_0703;PAS_chr1-4_0160;PAS_chr2-1_0453;PAS_chr2-1_0853;PAS_chr1-4_0234;PAS_chr4_0043;PAS_chr2-1_0684
map00100	Steroid biosynthesis	Metabolism	Lipid metabolism	gene-PAS_chr2-1_0391|gene-PAS_chr3_0507|gene-PAS_chr1-1_0070|gene-PAS_chr2-1_0466|gene-PAS_chr3_0124|gene-PAS_chr3_0053|gene-PAS_chr3_0957|gene-PAS_chr1-4_0367|gene-PAS_chr3_0252	PAS_chr2-1_0391;PAS_chr3_0507;PAS_chr1-1_0070;PAS_chr2-1_0466;PAS_chr3_0124;PAS_chr3_0053;PAS_chr3_0957;PAS_chr1-4_0367;PAS_chr3_0252
map04136	Autophagy - other	Cellular Processes	Transport and catabolism	gene-PAS_chr3_0042|gene-PAS_chr4_0459|gene-PAS_chr1-4_0310|gene-PAS_chr1-4_0555|gene-PAS_chr3_0931|gene-PAS_chr3_0688|gene-PAS_chr4_0478|gene-PAS_chr1-3_0168|gene-PAS_chr2-1_0641|gene-PAS_chr3_1016|gene-PAS_chr4_0510	PAS_chr3_0042;PAS_chr4_0459;PAS_chr1-4_0310;PAS_chr1-4_0555;PAS_chr3_0931;PAS_chr3_0688;PAS_chr4_0478;PAS_chr1-3_0168;PAS_chr2-1_0641;PAS_chr3_1016;PAS_chr4_0510
map03440	Homologous recombination	Genetic Information Processing	Replication and repair	gene-PAS_chr3_0851|gene-PAS_chr2-1_0153|gene-PAS_chr2-1_0135|gene-PAS_chr4_0034|gene-PAS_FragB_0039|gene-PAS_chr1-4_0513|gene-PAS_chr1-3_0143|gene-PAS_FragB_0056|gene-PAS_chr2-1_0041|gene-PAS_chr2-1_0163	PAS_chr3_0851;PAS_chr2-1_0153;PAS_chr2-1_0135;PAS_chr4_0034;PAS_FragB_0039;PAS_chr1-4_0513;PAS_chr1-3_0143;PAS_FragB_0056;PAS_chr2-1_0041;PAS_chr2-1_0163
map00310	Lysine degradation	Metabolism	Amino acid metabolism	gene-PAS_chr2-2_0494|gene-PAS_chr1-4_0304|gene-PAS_chr1-3_0024|gene-PAS_chr2-1_0453|gene-PAS_chr2-1_0853|gene-PAS_chr4_0043|gene-PAS_chr1-1_0129|gene-PAS_chr2-2_0152|gene-PAS_chr3_0528	PAS_chr2-2_0494;PAS_chr1-4_0304;PAS_chr1-3_0024;PAS_chr2-1_0453;PAS_chr2-1_0853;PAS_chr4_0043;PAS_chr1-1_0129;PAS_chr2-2_0152;PAS_chr3_0528
map04138	Autophagy - yeast	Cellular Processes	Transport and catabolism	gene-PAS_chr4_0062|gene-PAS_chr3_0688|gene-PAS_chr1-4_0310|gene-PAS_chr1-4_0606|gene-PAS_chr3_0042|gene-PAS_chr1-3_0306|gene-PAS_chr4_0614|gene-PAS_chr3_0895|gene-PAS_chr3_0931|gene-PAS_chr1-4_0500|gene-PAS_chr4_0480|gene-PAS_chr2-2_0343|gene-PAS_chr2-1_0641|gene-PAS_chr4_0510|gene-PAS_chr2-1_0672|gene-PAS_chr1-1_0150|gene-PAS_chr1-3_0213|gene-PAS_chr1-1_0097|gene-PAS_chr4_0459|gene-PAS_chr1-4_0548|gene-PAS_chr1-4_0555|gene-PAS_chr4_0079|gene-PAS_chr3_1175|gene-PAS_chr4_0478|gene-PAS_chr1-3_0168|gene-PAS_chr3_0148|gene-PAS_chr3_1016	PAS_chr4_0062;PAS_chr3_0688;PAS_chr1-4_0310;PAS_chr1-4_0606;PAS_chr3_0042;PAS_chr1-3_0306;PAS_chr4_0614;PAS_chr3_0895;PAS_chr3_0931;PAS_chr1-4_0500;PAS_chr4_0480;PAS_chr2-2_0343;PAS_chr2-1_0641;PAS_chr4_0510;PAS_chr2-1_0672;PAS_chr1-1_0150;PAS_chr1-3_0213;PAS_chr1-1_0097;PAS_chr4_0459;PAS_chr1-4_0548;PAS_chr1-4_0555;PAS_chr4_0079;PAS_chr3_1175;PAS_chr4_0478;PAS_chr1-3_0168;PAS_chr3_0148;PAS_chr3_1016
map04111	Cell cycle - yeast	Cellular Processes	Cell growth and death	gene-PAS_chr1-1_0340|gene-PAS_chr2-1_0508|gene-PAS_chr2-1_0276|gene-PAS_chr1-4_0054|gene-PAS_chr2-1_0082|gene-PAS_chr2-2_0297|gene-PAS_chr3_1061|gene-PAS_chr3_0758|gene-PAS_chr3_1218|gene-PAS_chr2-2_0495|gene-PAS_chr2-2_0040|gene-PAS_chr2-1_0762|gene-PAS_chr2-2_0307|gene-PAS_chr4_0525|gene-PAS_chr3_1001|gene-PAS_chr3_0594|gene-PAS_chr1-4_0363|gene-PAS_chr2-1_0092|gene-PAS_chr2-1_0507|gene-PAS_chr3_0071|gene-PAS_c121_0015|gene-PAS_FragB_0028|gene-PAS_chr3_1186|gene-PAS_chr4_0585|gene-PAS_chr1-3_0065|gene-PAS_chr3_0265|gene-PAS_chr4_0526|gene-PAS_chr1-3_0148|gene-PAS_chr1-1_0336|gene-PAS_chr2-1_0149|gene-PAS_chr4_0604|gene-PAS_chr4_0495|gene-PAS_chr3_0941|gene-PAS_chr1-4_0020	PAS_chr1-1_0340;PAS_chr2-1_0508;PAS_chr2-1_0276;PAS_chr1-4_0054;PAS_chr2-1_0082;PAS_chr2-2_0297;PAS_chr3_1061;PAS_chr3_0758;PAS_chr3_1218;PAS_chr2-2_0495;PAS_chr2-2_0040;PAS_chr2-1_0762;PAS_chr2-2_0307;PAS_chr4_0525;PAS_chr3_1001;PAS_chr3_0594;PAS_chr1-4_0363;PAS_chr2-1_0092;PAS_chr2-1_0507;PAS_chr3_0071;PAS_c121_0015;PAS_FragB_0028;PAS_chr3_1186;PAS_chr4_0585;PAS_chr1-3_0065;PAS_chr3_0265;PAS_chr4_0526;PAS_chr1-3_0148;PAS_chr1-1_0336;PAS_chr2-1_0149;PAS_chr4_0604;PAS_chr4_0495;PAS_chr3_0941;PAS_chr1-4_0020
map00561	Glycerolipid metabolism	Metabolism	Lipid metabolism	gene-PAS_chr3_0271|gene-PAS_chr2-1_0279|gene-PAS_chr3_0512|gene-PAS_chr2-1_0453|gene-PAS_chr4_0783|gene-PAS_chr3_0073|gene-PAS_chr2-1_0466|gene-PAS_chr1-3_0081|gene-PAS_chr3_0218|gene-PAS_chr4_0043|gene-PAS_chr1-1_0360|gene-PAS_chr2-1_0853	PAS_chr3_0271;PAS_chr2-1_0279;PAS_chr3_0512;PAS_chr2-1_0453;PAS_chr4_0783;PAS_chr3_0073;PAS_chr2-1_0466;PAS_chr1-3_0081;PAS_chr3_0218;PAS_chr4_0043;PAS_chr1-1_0360;PAS_chr2-1_0853
map00592	alpha-Linolenic acid metabolism	Metabolism	Lipid metabolism	gene-PAS_chr1-4_0538|gene-PAS_chr2-1_0466|gene-PAS_chr2-2_0267	PAS_chr1-4_0538;PAS_chr2-1_0466;PAS_chr2-2_0267
map00620	Pyruvate metabolism	Metabolism	Carbohydrate metabolism	gene-PAS_chr4_0191|gene-PAS_chr4_0815|gene-PAS_chr2-2_0294|gene-PAS_chr1-4_0304|gene-PAS_chr1-4_0459|gene-PAS_FragB_0061|gene-PAS_chr2-1_0453|gene-PAS_chr1-4_0593|gene-PAS_chr1-4_0249|gene-PAS_chr2-1_0853|gene-PAS_chr2-1_0767|gene-PAS_chr1-4_0421|gene-PAS_chr2-1_0769|gene-PAS_chr4_0043|gene-PAS_chr3_0253|gene-PAS_chr1-1_0050	PAS_chr4_0191;PAS_chr4_0815;PAS_chr2-2_0294;PAS_chr1-4_0304;PAS_chr1-4_0459;PAS_FragB_0061;PAS_chr2-1_0453;PAS_chr1-4_0593;PAS_chr1-4_0249;PAS_chr2-1_0853;PAS_chr2-1_0767;PAS_chr1-4_0421;PAS_chr2-1_0769;PAS_chr4_0043;PAS_chr3_0253;PAS_chr1-1_0050
map00630	Glyoxylate and dicarboxylate metabolism	Metabolism	Carbohydrate metabolism	gene-PAS_chr1-4_0459|gene-PAS_chr4_0416|gene-PAS_chr4_0587|gene-PAS_chr1-4_0304|gene-PAS_chr1-4_0338|gene-PAS_chr1-1_0475|gene-PAS_chr1-3_0104|gene-PAS_chr2-1_0767|gene-PAS_chr4_0815|gene-PAS_chr4_0191	PAS_chr1-4_0459;PAS_chr4_0416;PAS_chr4_0587;PAS_chr1-4_0304;PAS_chr1-4_0338;PAS_chr1-1_0475;PAS_chr1-3_0104;PAS_chr2-1_0767;PAS_chr4_0815;PAS_chr4_0191
map00360	Phenylalanine metabolism	Metabolism	Amino acid metabolism	gene-PAS_chr3_0283|gene-PAS_chr4_0147|gene-PAS_chr4_0314|gene-PAS_chr4_0974|gene-PAS_chr3_0095|gene-PAS_chr2-1_0684|gene-PAS_chr3_0864	PAS_chr3_0283;PAS_chr4_0147;PAS_chr4_0314;PAS_chr4_0974;PAS_chr3_0095;PAS_chr2-1_0684;PAS_chr3_0864
map00020	Citrate cycle (TCA cycle)	Metabolism	Carbohydrate metabolism	gene-PAS_chr3_0831|gene-PAS_FragB_0061|gene-PAS_chr1-1_0475|gene-PAS_chr1-1_0050|gene-PAS_chr1-4_0593|gene-PAS_chr1-3_0104|gene-PAS_chr2-1_0580|gene-PAS_chr2-2_0294|gene-PAS_chr4_0815|gene-PAS_chr2-2_0407	PAS_chr3_0831;PAS_FragB_0061;PAS_chr1-1_0475;PAS_chr1-1_0050;PAS_chr1-4_0593;PAS_chr1-3_0104;PAS_chr2-1_0580;PAS_chr2-2_0294;PAS_chr4_0815;PAS_chr2-2_0407
map00250	Alanine, aspartate and glutamate metabolism	Metabolism	Amino acid metabolism	gene-PAS_chr2-1_0343|gene-PAS_chr4_0416|gene-PAS_chr2-2_0142|gene-PAS_chr1-3_0024|gene-PAS_chr3_0743|gene-PAS_chr2-2_0481|gene-PAS_chr3_0482|gene-PAS_chr3_0675|gene-PAS_chr4_0974|gene-PAS_chr1-4_0227|gene-PAS_chr2-1_0311	PAS_chr2-1_0343;PAS_chr4_0416;PAS_chr2-2_0142;PAS_chr1-3_0024;PAS_chr3_0743;PAS_chr2-2_0481;PAS_chr3_0482;PAS_chr3_0675;PAS_chr4_0974;PAS_chr1-4_0227;PAS_chr2-1_0311
map00330	Arginine and proline metabolism	Metabolism	Amino acid metabolism	gene-PAS_chr4_0823|gene-PAS_chr1-3_0269|gene-PAS_chr3_0283|gene-PAS_chr2-1_0453|gene-PAS_chr4_0665|gene-PAS_chr2-1_0853|gene-PAS_chr3_0922|gene-PAS_chr4_0974|gene-PAS_chr4_0043|gene-PAS_chr1-4_0227	PAS_chr4_0823;PAS_chr1-3_0269;PAS_chr3_0283;PAS_chr2-1_0453;PAS_chr4_0665;PAS_chr2-1_0853;PAS_chr3_0922;PAS_chr4_0974;PAS_chr4_0043;PAS_chr1-4_0227
map00564	Glycerophospholipid metabolism	Metabolism	Lipid metabolism	gene-PAS_chr3_0570|gene-PAS_chr3_0271|gene-PAS_chr3_0512|gene-PAS_chr4_0153|gene-PAS_chr2-2_0286|gene-PAS_chr2-2_0063|gene-PAS_chr3_0073|gene-PAS_chr2-1_0466|gene-PAS_chr1-3_0081|gene-PAS_chr2-1_0786|gene-PAS_chr3_0845|gene-PAS_chr1-3_0238|gene-PAS_chr3_1180	PAS_chr3_0570;PAS_chr3_0271;PAS_chr3_0512;PAS_chr4_0153;PAS_chr2-2_0286;PAS_chr2-2_0063;PAS_chr3_0073;PAS_chr2-1_0466;PAS_chr1-3_0081;PAS_chr2-1_0786;PAS_chr3_0845;PAS_chr1-3_0238;PAS_chr3_1180
map00030	Pentose phosphate pathway	Metabolism	Carbohydrate metabolism	gene-PAS_chr1-4_0264|gene-PAS_chr3_0456|gene-PAS_chr2-2_0337|gene-PAS_chr3_0868|gene-PAS_chr1-4_0047|gene-PAS_chr2-1_0402|gene-PAS_chr3_0604|gene-PAS_chr4_0213|gene-PAS_chr1-1_0072	PAS_chr1-4_0264;PAS_chr3_0456;PAS_chr2-2_0337;PAS_chr3_0868;PAS_chr1-4_0047;PAS_chr2-1_0402;PAS_chr3_0604;PAS_chr4_0213;PAS_chr1-1_0072
map00520	Amino sugar and nucleotide sugar metabolism	Metabolism	Carbohydrate metabolism	gene-PAS_chr4_0149|gene-PAS_chr1-4_0561|gene-PAS_chr1-1_0393|gene-PAS_chr4_0624|gene-PAS_chr1-3_0122|gene-PAS_chr3_0456|gene-PAS_chr1-1_0067|gene-PAS_chr1-4_0447|gene-PAS_chr1-4_0264	PAS_chr4_0149;PAS_chr1-4_0561;PAS_chr1-1_0393;PAS_chr4_0624;PAS_chr1-3_0122;PAS_chr3_0456;PAS_chr1-1_0067;PAS_chr1-4_0447;PAS_chr1-4_0264
map00280	Valine, leucine and isoleucine degradation	Metabolism	Amino acid metabolism	gene-PAS_chr1-4_0304|gene-PAS_chr2-1_0453|gene-PAS_chr3_0937|gene-PAS_chr2-2_0267|gene-PAS_chr4_0043|gene-PAS_chr2-1_0853	PAS_chr1-4_0304;PAS_chr2-1_0453;PAS_chr3_0937;PAS_chr2-2_0267;PAS_chr4_0043;PAS_chr2-1_0853
map00860	Porphyrin metabolism	Metabolism	Metabolism of cofactors and vitamins	gene-PAS_chr4_0449|gene-PAS_chr4_0418|gene-PAS_chr1-3_0078|gene-PAS_chr3_0264|gene-PAS_chr4_0055|gene-PAS_chr3_0943|gene-PAS_chr1-4_0222	PAS_chr4_0449;PAS_chr4_0418;PAS_chr1-3_0078;PAS_chr3_0264;PAS_chr4_0055;PAS_chr3_0943;PAS_chr1-4_0222
map00380	Tryptophan metabolism	Metabolism	Amino acid metabolism	gene-PAS_chr3_0283|gene-PAS_chr1-4_0304|gene-PAS_chr2-2_0311|gene-PAS_chr2-1_0453|gene-PAS_chr2-1_0853|gene-PAS_chr3_1194|gene-PAS_chr4_0043	PAS_chr3_0283;PAS_chr1-4_0304;PAS_chr2-2_0311;PAS_chr2-1_0453;PAS_chr2-1_0853;PAS_chr3_1194;PAS_chr4_0043
map00430	Taurine and hypotaurine metabolism	Metabolism	Metabolism of other amino acids	gene-PAS_chr2-1_0343|gene-PAS_chr2-2_0322|gene-PAS_chr2-1_0311	PAS_chr2-1_0343;PAS_chr2-2_0322;PAS_chr2-1_0311
map00730	Thiamine metabolism	Metabolism	Metabolism of cofactors and vitamins	gene-PAS_chr3_0037|gene-PAS_chr3_0052|gene-PAS_chr2-2_0084|gene-PAS_chr3_0648|gene-PAS_chr3_0842	PAS_chr3_0037;PAS_chr3_0052;PAS_chr2-2_0084;PAS_chr3_0648;PAS_chr3_0842
map00640	Propanoate metabolism	Metabolism	Carbohydrate metabolism	gene-PAS_chr3_0831|gene-PAS_chr1-4_0538|gene-PAS_chr1-4_0249|gene-PAS_chr3_0937|gene-PAS_chr2-1_0767|gene-PAS_chr2-1_0776|gene-PAS_chr2-2_0407	PAS_chr3_0831;PAS_chr1-4_0538;PAS_chr1-4_0249;PAS_chr3_0937;PAS_chr2-1_0767;PAS_chr2-1_0776;PAS_chr2-2_0407
map04113	Meiosis - yeast	Cellular Processes	Cell growth and death	gene-PAS_chr1-4_0179|gene-PAS_chr1-1_0434|gene-PAS_chr2-1_0276|gene-PAS_chr1-4_0054|gene-PAS_chr2-1_0082|gene-PAS_chr2-2_0297|gene-PAS_chr3_1218|gene-PAS_chr3_1061|gene-PAS_chr4_0326|gene-PAS_chr4_0480|gene-PAS_chr1-1_0300|gene-PAS_chr1-4_0530|gene-PAS_chr4_0604|gene-PAS_chr2-1_0078|gene-PAS_FragB_0028|gene-PAS_chr4_0585|gene-PAS_chr3_0148|gene-PAS_chr4_0526|gene-PAS_chr1-3_0148|gene-PAS_chr2-1_0149|gene-PAS_chr1-4_0473|gene-PAS_chr4_0495|gene-PAS_chr3_0941|gene-PAS_chr1-4_0570|gene-PAS_chr1-4_0020	PAS_chr1-4_0179;PAS_chr1-1_0434;PAS_chr2-1_0276;PAS_chr1-4_0054;PAS_chr2-1_0082;PAS_chr2-2_0297;PAS_chr3_1218;PAS_chr3_1061;PAS_chr4_0326;PAS_chr4_0480;PAS_chr1-1_0300;PAS_chr1-4_0530;PAS_chr4_0604;PAS_chr2-1_0078;PAS_FragB_0028;PAS_chr4_0585;PAS_chr3_0148;PAS_chr4_0526;PAS_chr1-3_0148;PAS_chr2-1_0149;PAS_chr1-4_0473;PAS_chr4_0495;PAS_chr3_0941;PAS_chr1-4_0570;PAS_chr1-4_0020
map00770	Pantothenate and CoA biosynthesis	Metabolism	Metabolism of cofactors and vitamins	gene-PAS_chr4_0823|gene-PAS_chr2-2_0204|gene-PAS_chr2-1_0453|gene-PAS_chr4_0793|gene-PAS_chr2-1_0853|gene-PAS_chr3_0922|gene-PAS_chr4_0043|gene-PAS_chr1-4_0198	PAS_chr4_0823;PAS_chr2-2_0204;PAS_chr2-1_0453;PAS_chr4_0793;PAS_chr2-1_0853;PAS_chr3_0922;PAS_chr4_0043;PAS_chr1-4_0198
map00565	Ether lipid metabolism	Metabolism	Lipid metabolism	gene-PAS_chr2-1_0466|gene-PAS_chr3_0271|gene-PAS_chr2-2_0063	PAS_chr2-1_0466;PAS_chr3_0271;PAS_chr2-2_0063
map02010	ABC transporters	Environmental Information Processing	Membrane transport	gene-PAS_chr4_0186|gene-PAS_chr3_0822|gene-PAS_chr4_0188|gene-PAS_chr1-4_0562|gene-PAS_chr2-2_0272|gene-PAS_chr2-2_0299	PAS_chr4_0186;PAS_chr3_0822;PAS_chr4_0188;PAS_chr1-4_0562;PAS_chr2-2_0272;PAS_chr2-2_0299
map00760	Nicotinate and nicotinamide metabolism	Metabolism	Metabolism of cofactors and vitamins	gene-PAS_chr2-2_0049|gene-PAS_chr1-3_0130|gene-PAS_chr4_0233|gene-PAS_chr1-3_0024|gene-PAS_chr3_0915|gene-PAS_chr2-1_0221	PAS_chr2-2_0049;PAS_chr1-3_0130;PAS_chr4_0233;PAS_chr1-3_0024;PAS_chr3_0915;PAS_chr2-1_0221
map00999	Biosynthesis of various plant secondary metabolites	Metabolism	Biosynthesis of other secondary metabolites	gene-PAS_chr4_0013|gene-PAS_chr3_0876	PAS_chr4_0013;PAS_chr3_0876
map04814	Motor proteins	Cellular Processes	Cell motility	gene-PAS_chr4_0148|gene-PAS_FragB_0012|gene-PAS_chr1-1_0263|gene-PAS_chr4_0160|gene-PAS_c131_0006|gene-PAS_chr2-1_0186|gene-PAS_chr4_0533|gene-PAS_chr1-4_0443	PAS_chr4_0148;PAS_FragB_0012;PAS_chr1-1_0263;PAS_chr4_0160;PAS_c131_0006;PAS_chr2-1_0186;PAS_chr4_0533;PAS_chr1-4_0443
map04392	Hippo signaling pathway - multiple species	Environmental Information Processing	Signal transduction	gene-PAS_chr3_1061|gene-PAS_chr3_0727|gene-PAS_c131_0004|gene-PAS_chr3_0092	PAS_chr3_1061;PAS_chr3_0727;PAS_c131_0004;PAS_chr3_0092
map00053	Ascorbate and aldarate metabolism	Metabolism	Carbohydrate metabolism	gene-PAS_chr2-1_0775|gene-PAS_chr4_0043|gene-PAS_chr2-1_0853|gene-PAS_chr2-1_0453	PAS_chr2-1_0775;PAS_chr4_0043;PAS_chr2-1_0853;PAS_chr2-1_0453
map00460	Cyanoamino acid metabolism	Metabolism	Metabolism of other amino acids	gene-PAS_chr2-2_0481|gene-PAS_chr4_0013|gene-PAS_chr4_0587	PAS_chr2-2_0481;PAS_chr4_0013;PAS_chr4_0587
map00254	Aflatoxin biosynthesis	Metabolism	Biosynthesis of other secondary metabolites	gene-PAS_chr1-4_0249	PAS_chr1-4_0249
map00591	Linoleic acid metabolism	Metabolism	Lipid metabolism	gene-PAS_chr2-1_0466	PAS_chr2-1_0466

map00650	Butanoate metabolism	Metabolism	Carbohydrate metabolism	gene-PAS_chr1-4_0304|gene-PAS_chr2-1_0343|gene-PAS_chr2-1_0313|gene-PAS_chr1-3_0024	PAS_chr1-4_0304;PAS_chr2-1_0343;PAS_chr2-1_0313;PAS_chr1-3_0024
map00261	Monobactam biosynthesis	Metabolism	Biosynthesis of other secondary metabolites	gene-PAS_chr1-1_0175|gene-PAS_chr1-4_0253	PAS_chr1-1_0175;PAS_chr1-4_0253
map03450	Non-homologous end-joining	Genetic Information Processing	Replication and repair	gene-PAS_chr1-4_0513|gene-PAS_chr3_0851|gene-PAS_chr4_0751	PAS_chr1-4_0513;PAS_chr3_0851;PAS_chr4_0751
map04122	Sulfur relay system	Genetic Information Processing	Folding, sorting and degradation	gene-PAS_chr3_0687|gene-PAS_chr3_0037|gene-PAS_chr1-4_0547|gene-PAS_chr2-1_0563	PAS_chr3_0687;PAS_chr3_0037;PAS_chr1-4_0547;PAS_chr2-1_0563
map00270	Cysteine and methionine metabolism	Metabolism	Amino acid metabolism	gene-PAS_chr4_0815|gene-PAS_chr2-2_0137|gene-PAS_chr2-2_0322|gene-PAS_chr4_0664|gene-PAS_chr4_0330|gene-PAS_chr4_0974|gene-PAS_chr2-1_0862|gene-PAS_chr2-1_0160|gene-PAS_chr4_0865|gene-PAS_chr3_0876	PAS_chr4_0815;PAS_chr2-2_0137;PAS_chr2-2_0322;PAS_chr4_0664;PAS_chr4_0330;PAS_chr4_0974;PAS_chr2-1_0862;PAS_chr2-1_0160;PAS_chr4_0865;PAS_chr3_0876
map00300	Lysine biosynthesis	Metabolism	Amino acid metabolism	gene-PAS_chr1-4_0153|gene-PAS_chr1-4_0421|gene-PAS_chr1-1_0175|gene-PAS_chr3_0528	PAS_chr1-4_0153;PAS_chr1-4_0421;PAS_chr1-1_0175;PAS_chr3_0528
map00600	Sphingolipid metabolism	Metabolism	Lipid metabolism	gene-PAS_chr1-4_0149|gene-PAS_chr4_0866|gene-PAS_chr3_0399|gene-PAS_chr3_0425	PAS_chr1-4_0149;PAS_chr4_0866;PAS_chr3_0399;PAS_chr3_0425
map00670	One carbon pool by folate	Metabolism	Metabolism of cofactors and vitamins	gene-PAS_chr2-1_0121|gene-PAS_chr1-1_0212|gene-PAS_chr2-2_0145|gene-PAS_chr4_0587	PAS_chr2-1_0121;PAS_chr1-1_0212;PAS_chr2-2_0145;PAS_chr4_0587
map00220	Arginine biosynthesis	Metabolism	Amino acid metabolism	gene-PAS_chr4_0974|gene-PAS_chr1-1_0127|gene-PAS_chr3_0482|gene-PAS_chr2-1_0311|gene-PAS_chr1-4_0215	PAS_chr4_0974;PAS_chr1-1_0127;PAS_chr3_0482;PAS_chr2-1_0311;PAS_chr1-4_0215
map04011	MAPK signaling pathway - yeast	Environmental Information Processing	Signal transduction	gene-PAS_chr4_0636|gene-PAS_chr3_0220|gene-PAS_chr3_0303|gene-PAS_chr3_0299|gene-PAS_chr1-3_0149|gene-PAS_c131_0004|gene-PAS_chr2-1_0762|gene-PAS_chr2-1_0586|gene-PAS_chr1-1_0201|gene-PAS_chr4_0123|gene-PAS_chr3_0840|gene-PAS_chr3_0092|gene-PAS_chr3_1001|gene-PAS_chr2-1_0263|gene-PAS_chr2-1_0203|gene-PAS_chr1-4_0279|gene-PAS_chr2-2_0095|gene-PAS_chr4_0986|gene-PAS_chr3_1157|gene-PAS_chr1-4_0438|gene-PAS_chr3_0895|gene-PAS_chr1-3_0225	PAS_chr4_0636;PAS_chr3_0220;PAS_chr3_0303;PAS_chr3_0299;PAS_chr1-3_0149;PAS_c131_0004;PAS_chr2-1_0762;PAS_chr2-1_0586;PAS_chr1-1_0201;PAS_chr4_0123;PAS_chr3_0840;PAS_chr3_0092;PAS_chr3_1001;PAS_chr2-1_0263;PAS_chr2-1_0203;PAS_chr1-4_0279;PAS_chr2-2_0095;PAS_chr4_0986;PAS_chr3_1157;PAS_chr1-4_0438;PAS_chr3_0895;PAS_chr1-3_0225
map00562	Inositol phosphate metabolism	Metabolism	Carbohydrate metabolism	gene-PAS_chr2-2_0113|gene-PAS_chr4_0700|gene-PAS_chr1-4_0380|gene-PAS_chr2-1_0178|gene-PAS_chr4_0478|gene-PAS_chr3_0951	PAS_chr2-2_0113;PAS_chr4_0700;PAS_chr1-4_0380;PAS_chr2-1_0178;PAS_chr4_0478;PAS_chr3_0951
map00350	Tyrosine metabolism	Metabolism	Amino acid metabolism	gene-PAS_chr4_0974|gene-PAS_chr2-1_0684|gene-PAS_chr1-3_0024|gene-PAS_chr4_0147	PAS_chr4_0974;PAS_chr2-1_0684;PAS_chr1-3_0024;PAS_chr4_0147
map00909	Sesquiterpenoid and triterpenoid biosynthesis	Metabolism	Metabolism of terpenoids and polyketides	gene-PAS_chr3_0124	PAS_chr3_0124
map00332	Carbapenem biosynthesis	Metabolism	Biosynthesis of other secondary metabolites	gene-PAS_chr4_0665	PAS_chr4_0665
map00590	Arachidonic acid metabolism	Metabolism	Lipid metabolism	gene-PAS_chr2-1_0466	PAS_chr2-1_0466
map03040	Spliceosome	Genetic Information Processing	Transcription	gene-PAS_chr3_1252|gene-PAS_chr3_0420|gene-PAS_chr3_0496|gene-PAS_chr1-1_0317|gene-PAS_chr3_0992|gene-PAS_chr3_0665|gene-PAS_chr1-3_0111|gene-PAS_chr4_0083|gene-PAS_chr2-1_0418|gene-PAS_chr1-1_0196|gene-PAS_chr3_0325|gene-PAS_chr2-1_0662|gene-PAS_chr1-1_0383|gene-PAS_chr1-4_0118|gene-PAS_chr2-2_0187|gene-PAS_chr2-2_0340|gene-PAS_chr3_0230|gene-PAS_chr4_0538|gene-PAS_chr4_0448	PAS_chr3_1252;PAS_chr3_0420;PAS_chr3_0496;PAS_chr1-1_0317;PAS_chr3_0992;PAS_chr3_0665;PAS_chr1-3_0111;PAS_chr4_0083;PAS_chr2-1_0418;PAS_chr1-1_0196;PAS_chr3_0325;PAS_chr2-1_0662;PAS_chr1-1_0383;PAS_chr1-4_0118;PAS_chr2-2_0187;PAS_chr2-2_0340;PAS_chr3_0230;PAS_chr4_0538;PAS_chr4_0448
map00660	C5-Branched dibasic acid metabolism	Metabolism	Carbohydrate metabolism	gene-PAS_chr3_0039	PAS_chr3_0039
map00785	Lipoic acid metabolism	Metabolism	Metabolism of cofactors and vitamins	gene-PAS_chr1-4_0593|gene-PAS_chr2-2_0294|gene-PAS_chr1-1_0050	PAS_chr1-4_0593;PAS_chr2-2_0294;PAS_chr1-1_0050
map00480	Glutathione metabolism	Metabolism	Metabolism of other amino acids	gene-PAS_chr4_0197|gene-PAS_chr2-1_0580|gene-PAS_chr1-1_0433|gene-PAS_chr1-1_0282|gene-PAS_chr2-1_0033	PAS_chr4_0197;PAS_chr2-1_0580;PAS_chr1-1_0433;PAS_chr1-1_0282;PAS_chr2-1_0033
map00450	Selenocompound metabolism	Metabolism	Metabolism of other amino acids	gene-PAS_chr1-4_0253|gene-PAS_chr2-1_0160	PAS_chr1-4_0253;PAS_chr2-1_0160
map03082	ATP-dependent chromatin remodeling	Genetic Information Processing	Chromosome	gene-PAS_FragB_0005|gene-PAS_chr1-4_0571|gene-PAS_chr1-4_0084|gene-PAS_chr4_0143|gene-PAS_chr2-1_0429|gene-PAS_chr1-1_0020|gene-PAS_chr3_1191	PAS_FragB_0005;PAS_chr1-4_0571;PAS_chr1-4_0084;PAS_chr4_0143;PAS_chr2-1_0429;PAS_chr1-1_0020;PAS_chr3_1191
map03018	RNA degradation	Genetic Information Processing	Folding, sorting and degradation	gene-PAS_chr3_0273|gene-PAS_chr4_0448|gene-PAS_chr4_0633|gene-PAS_chr1-4_0283|gene-PAS_chr1-4_0047|gene-PAS_chr4_0194|gene-PAS_chr1-1_0143|gene-PAS_chr2-1_0402|gene-PAS_chr1-3_0157|gene-PAS_chr3_0082|gene-PAS_chr2-1_0494	PAS_chr3_0273;PAS_chr4_0448;PAS_chr4_0633;PAS_chr1-4_0283;PAS_chr1-4_0047;PAS_chr4_0194;PAS_chr1-1_0143;PAS_chr2-1_0402;PAS_chr1-3_0157;PAS_chr3_0082;PAS_chr2-1_0494
map03015	mRNA surveillance pathway	Genetic Information Processing	Translation	gene-PAS_chr4_0528|gene-PAS_chr2-1_0528|gene-PAS_chr2-1_0816|gene-PAS_chr1-4_0283|gene-PAS_FragB_0063|gene-PAS_chr2-1_0662|gene-PAS_chr4_0495|gene-PAS_chr1-3_0183|gene-PAS_chr2-1_0233	PAS_chr4_0528;PAS_chr2-1_0528;PAS_chr2-1_0816;PAS_chr1-4_0283;PAS_FragB_0063;PAS_chr2-1_0662;PAS_chr4_0495;PAS_chr1-3_0183;PAS_chr2-1_0233
map04213	Longevity regulating pathway - multiple species	Organismal Systems	Aging	gene-PAS_chr4_0459|gene-PAS_chr2-1_0078|gene-PAS_chr1-3_0102|gene-PAS_chr3_0230|gene-PAS_chr3_0148|gene-PAS_chr2-1_0324	PAS_chr4_0459;PAS_chr2-1_0078;PAS_chr1-3_0102;PAS_chr3_0230;PAS_chr3_0148;PAS_chr2-1_0324
map00040	Pentose and glucuronate interconversions	Metabolism	Carbohydrate metabolism	gene-PAS_chr4_0572|gene-PAS_chr1-3_0122	PAS_chr4_0572;PAS_chr1-3_0122
map03030	DNA replication	Genetic Information Processing	Replication and repair	gene-PAS_chr2-1_0149|gene-PAS_chr2-1_0500|gene-PAS_chr4_0279|gene-PAS_chr4_0034|gene-PAS_chr4_0323|gene-PAS_chr3_0233|gene-PAS_chr2-1_0163	PAS_chr2-1_0149;PAS_chr2-1_0500;PAS_chr4_0279;PAS_chr4_0034;PAS_chr4_0323;PAS_chr3_0233;PAS_chr2-1_0163
map00750	Vitamin B6 metabolism	Metabolism	Metabolism of cofactors and vitamins	gene-PAS_chr1-4_0393|gene-PAS_chr1-1_0448	PAS_chr1-4_0393;PAS_chr1-1_0448
map03013	Nucleocytoplasmic transport	Genetic Information Processing	Translation	gene-PAS_chr3_0420|gene-PAS_chr2-1_0528|gene-PAS_chr4_0163|gene-PAS_chr3_0993|gene-PAS_chr2-2_0385|gene-PAS_chr2-1_0562|gene-PAS_chr3_0524|gene-PAS_chr2-1_0662|gene-PAS_chr1-3_0071|gene-PAS_chr2-2_0270|gene-PAS_chr3_0715|gene-PAS_chr4_0528|gene-PAS_chr3_1039	PAS_chr3_0420;PAS_chr2-1_0528;PAS_chr4_0163;PAS_chr3_0993;PAS_chr2-2_0385;PAS_chr2-1_0562;PAS_chr3_0524;PAS_chr2-1_0662;PAS_chr1-3_0071;PAS_chr2-2_0270;PAS_chr3_0715;PAS_chr4_0528;PAS_chr3_1039
map00740	Riboflavin metabolism	Metabolism	Metabolism of cofactors and vitamins	gene-PAS_chr3_0156|gene-PAS_chr3_0167	PAS_chr3_0156;PAS_chr3_0167
map00910	Nitrogen metabolism	Metabolism	Energy metabolism	gene-PAS_chr2-1_0311	PAS_chr2-1_0311
map03410	Base excision repair	Genetic Information Processing	Replication and repair	gene-PAS_chr3_0233|gene-PAS_chr4_0492|gene-PAS_chr1-3_0257|gene-PAS_chr2-1_0163	PAS_chr3_0233;PAS_chr4_0492;PAS_chr1-3_0257;PAS_chr2-1_0163
map00563	Glycosylphosphatidylinositol (GPI)-anchor biosynthesis	Metabolism	Glycan biosynthesis and metabolism	gene-PAS_chr1-4_0333|gene-PAS_chr1-4_0470|gene-PAS_chr3_0454|gene-PAS_chr3_0369	PAS_chr1-4_0333;PAS_chr1-4_0470;PAS_chr3_0454;PAS_chr3_0369
map01040	Biosynthesis of unsaturated fatty acids	Metabolism	Lipid metabolism	gene-PAS_chr1-4_0538|gene-PAS_chr2-2_0267	PAS_chr1-4_0538;PAS_chr2-2_0267
map03022	Basal transcription factors	Genetic Information Processing	Transcription	gene-PAS_chr4_0204	PAS_chr4_0204
map00240	Pyrimidine metabolism	Metabolism	Nucleotide metabolism	gene-PAS_chr2-2_0142|gene-PAS_chr4_0197|gene-PAS_chr1-1_0282|gene-PAS_chr2-2_0021|gene-PAS_chr1-4_0460|gene-PAS_chr2-1_0770	PAS_chr2-2_0142;PAS_chr4_0197;PAS_chr1-1_0282;PAS_chr2-2_0021;PAS_chr1-4_0460;PAS_chr2-1_0770
map00790	Folate biosynthesis	Metabolism	Metabolism of cofactors and vitamins	gene-PAS_chr3_0687|gene-PAS_chr2-2_0084|gene-PAS_chr2-2_0302	PAS_chr3_0687;PAS_chr2-2_0084;PAS_chr2-2_0302
map03430	Mismatch repair	Genetic Information Processing	Replication and repair	gene-PAS_chr3_0233|gene-PAS_chr2-1_0163|gene-PAS_chr4_0034|gene-PAS_chr3_0739	PAS_chr3_0233;PAS_chr2-1_0163;PAS_chr4_0034;PAS_chr3_0739
map04070	Phosphatidylinositol signaling system	Environmental Information Processing	Signal transduction	gene-PAS_chr4_0478|gene-PAS_chr4_0700|gene-PAS_chr1-4_0380|gene-PAS_chr2-1_0178	PAS_chr4_0478;PAS_chr4_0700;PAS_chr1-4_0380;PAS_chr2-1_0178
map04139	Mitophagy - yeast	Cellular Processes	Transport and catabolism	gene-PAS_chr2-1_0480|gene-PAS_chr1-4_0555|gene-PAS_chr3_0895|gene-PAS_chr3_0220|gene-PAS_chr4_0480|gene-PAS_chr3_0245|gene-PAS_chr2-1_0641	PAS_chr2-1_0480;PAS_chr1-4_0555;PAS_chr3_0895;PAS_chr3_0220;PAS_chr4_0480;PAS_chr3_0245;PAS_chr2-1_0641
map03083	Polycomb repressive complex	Genetic Information Processing	Chromosome	gene-PAS_chr1-1_0295|gene-PAS_chr3_1191	PAS_chr1-1_0295;PAS_chr3_1191
map04145	Phagosome	Cellular Processes	Transport and catabolism	gene-PAS_chr2-1_0186|gene-PAS_chr4_0478|gene-PAS_chr4_0533|gene-PAS_chr2-1_0322|gene-PAS_chr1-4_0443|gene-PAS_chr1-4_0626	PAS_chr2-1_0186;PAS_chr4_0478;PAS_chr4_0533;PAS_chr2-1_0322;PAS_chr1-4_0443;PAS_chr1-4_0626
map00260	Glycine, serine and threonine metabolism	Metabolism	Amino acid metabolism	gene-PAS_chr3_0826|gene-PAS_chr4_0416|gene-PAS_chr2-2_0137|gene-PAS_chr4_0587|gene-PAS_chr4_0285|gene-PAS_chr3_0693	PAS_chr3_0826;PAS_chr4_0416;PAS_chr2-2_0137;PAS_chr4_0587;PAS_chr4_0285;PAS_chr3_0693
map04120	Ubiquitin mediated proteolysis	Genetic Information Processing	Folding, sorting and degradation	gene-PAS_chr2-1_0119|gene-PAS_chr3_0594|gene-PAS_chr3_1218|gene-PAS_chr2-2_0495|gene-PAS_chr1-4_0670|gene-PAS_chr1-3_0148|gene-PAS_chr3_0924|gene-PAS_chr1-4_0020	PAS_chr2-1_0119;PAS_chr3_0594;PAS_chr3_1218;PAS_chr2-2_0495;PAS_chr1-4_0670;PAS_chr1-3_0148;PAS_chr3_0924;PAS_chr1-4_0020
map00400	Phenylalanine, tyrosine and tryptophan biosynthesis	Metabolism	Amino acid metabolism	gene-PAS_chr4_0974|gene-PAS_chr2-1_0684|gene-PAS_chr4_0147	PAS_chr4_0974;PAS_chr2-1_0684;PAS_chr4_0147
map00130	Ubiquinone and other terpenoid-quinone biosynthesis	Metabolism	Metabolism of cofactors and vitamins	gene-PAS_chr1-1_0449	PAS_chr1-1_0449
map03420	Nucleotide excision repair	Genetic Information Processing	Replication and repair	gene-PAS_chr2-1_0163|gene-PAS_chr4_0034|gene-PAS_chr4_0204|gene-PAS_chr4_0555|gene-PAS_chr3_0233|gene-PAS_chr2-2_0184|gene-PAS_chr1-1_0025|gene-PAS_chr1-1_0166	PAS_chr2-1_0163;PAS_chr4_0034;PAS_chr4_0204;PAS_chr4_0555;PAS_chr3_0233;PAS_chr2-2_0184;PAS_chr1-1_0025;PAS_chr1-1_0166
map00230	Purine metabolism	Metabolism	Nucleotide metabolism	gene-PAS_chr2-1_0121|gene-PAS_chr1-4_0264|gene-PAS_chr3_0770|gene-PAS_chr3_0667|gene-PAS_chr4_0197|gene-PAS_chr1-1_0282|gene-PAS_chr1-4_0566|gene-PAS_chr1-4_0253	PAS_chr2-1_0121;PAS_chr1-4_0264;PAS_chr3_0770;PAS_chr3_0667;PAS_chr4_0197;PAS_chr1-1_0282;PAS_chr1-4_0566;PAS_chr1-4_0253
map00290	Valine, leucine and isoleucine biosynthesis	Metabolism	Amino acid metabolism	gene-PAS_chr3_0039	PAS_chr3_0039
map00900	Terpenoid backbone biosynthesis	Metabolism	Metabolism of terpenoids and polyketides	gene-PAS_chr1-4_0304|gene-PAS_chr2-2_0230	PAS_chr1-4_0304;PAS_chr2-2_0230
map03060	Protein export	Genetic Information Processing	Folding, sorting and degradation	gene-PAS_chr2-1_0140|gene-PAS_chr2-1_0636	PAS_chr2-1_0140;PAS_chr2-1_0636
map04144	Endocytosis	Cellular Processes	Transport and catabolism	gene-PAS_chr1-1_0150|gene-PAS_chr3_0990|gene-PAS_chr1-3_0261|gene-PAS_chr1-3_0309|gene-PAS_chr4_0993|gene-PAS_chr3_1047|gene-PAS_c131_0006|gene-PAS_chr4_0214|gene-PAS_chr3_0230|gene-PAS_chr1-4_0626	PAS_chr1-1_0150;PAS_chr3_0990;PAS_chr1-3_0261;PAS_chr1-3_0309;PAS_chr4_0993;PAS_chr3_1047;PAS_c131_0006;PAS_chr4_0214;PAS_chr3_0230;PAS_chr1-4_0626
map04141	Protein processing in endoplasmic reticulum	Genetic Information Processing	Folding, sorting and degradation	gene-PAS_chr1-1_0011|gene-PAS_chr1-3_0125|gene-PAS_chr4_0991|gene-PAS_chr1-3_0114|gene-PAS_chr3_0929|gene-PAS_chr2-1_0322|gene-PAS_chr1-1_0237|gene-PAS_chr3_0230|gene-PAS_chr2-1_0140|gene-PAS_chr3_0924	PAS_chr1-1_0011;PAS_chr1-3_0125;PAS_chr4_0991;PAS_chr1-3_0114;PAS_chr3_0929;PAS_chr2-1_0322;PAS_chr1-1_0237;PAS_chr3_0230;PAS_chr2-1_0140;PAS_chr3_0924
map00513	Various types of N-glycan biosynthesis	Metabolism	Glycan biosynthesis and metabolism	gene-PAS_chr4_0544|gene-PAS_chr2-1_0759	PAS_chr4_0544;PAS_chr2-1_0759
map04130	SNARE interactions in vesicular transport	Genetic Information Processing	Folding, sorting and degradation	gene-PAS_chr1-4_0500	PAS_chr1-4_0500
map00510	N-Glycan biosynthesis	Metabolism	Glycan biosynthesis and metabolism	gene-PAS_chr4_0544|gene-PAS_chr2-1_0759	PAS_chr4_0544;PAS_chr2-1_0759
map00190	Oxidative phosphorylation	Metabolism	Energy metabolism	gene-PAS_chr3_0824|gene-PAS_chr4_0449|gene-PAS_chr4_0422|gene-PAS_chr2-1_0363|gene-PAS_chr2-1_0361|gene-PAS_chr3_0615|gene-PAS_chr2-1_0746|gene-PAS_chr1-3_0070|gene-PAS_chr2-2_0265	PAS_chr3_0824;PAS_chr4_0449;PAS_chr4_0422;PAS_chr2-1_0363;PAS_chr2-1_0361;PAS_chr3_0615;PAS_chr2-1_0746;PAS_chr1-3_0070;PAS_chr2-2_0265
map00970	Aminoacyl-tRNA biosynthesis	Genetic Information Processing	Translation	gene-PAS_chr2-2_0209|gene-PAS_chr1-1_0212	PAS_chr2-2_0209;PAS_chr1-1_0212
map03020	RNA polymerase	Genetic Information Processing	Transcription	gene-PAS_chr1-1_0166	PAS_chr1-1_0166
map03008	Ribosome biogenesis in eukaryotes	Genetic Information Processing	Translation	gene-PAS_chr4_0419|gene-PAS_chr2-2_0045|gene-PAS_chr1-1_0187|gene-PAS_chr3_0681|gene-PAS_chr4_0528|gene-PAS_chr1-1_0377	PAS_chr4_0419;PAS_chr2-2_0045;PAS_chr1-1_0187;PAS_chr3_0681;PAS_chr4_0528;PAS_chr1-1_0377
