# Supplementary material for: Cross Kingdom Metabolic Engineering Paradigm Elevating Sustainable Protein Production
Source: Adv Sci (Weinh). 2026 Jun 23;13(52):e17703. doi: 10.1002/advs.202517703 (PMC13336901; doi:10.1002/advs.202517703)
Supplement: Supplementary file 4 [file ADVS-13-e17703-s005.pdf]

Table S1.DEGs identified by RNA-seq

Gene Name	Gene Description	FC(H_III6_II8_I4/HTX33)	Log2FC(H_III6_II8_I4/HTX33)	Pvalue	Padjust	Significant	Regulate
PAS_chr4_0424	Hypothetical protein	12.389	3.631026736	4.52896890115E-26	1.3837293198E-25	yes	up
PAS_chr4_0296	Hypothetical protein	8.742	3.128041538	1.33702532124E-7	2.14068778749E-7	yes	up
PAS_chr1-1_0354	Allantoin permease	16.099	4.008930326	1.20457753122E-180	1.66423637487E-179	yes	up
PAS_chr4_0973	uncharacterized protein	6.236	2.640623413	3.18771832758E-28	1.02369319728E-27	yes	up
PAS_chr3_0864	Hypothetical protein	9.959	3.316025239	1.8330688329E-114	1.80754963934E-113	yes	up
PAS_chr4_0352	Long chain fatty acyl-CoA synthetase	28.301	4.822792184	0	0	yes	up
PAS_chr2-1_0260	Hypothetical protein	6.002	2.585437494	7.58186381668E-122	7.91062098218E-121	yes	up
PAS_chr3_0874	Peroxisomal integral membrane peroxin	7.044	2.816381778	5.50370662046E-68	3.45113972497E-67	yes	up
PAS_chr3_1045	Peroxisome biosynthesis protein PAS1	9.237	3.207494068	2.30839557433E-268	4.26798578798E-267	yes	up
PAS_chr2-1_0569	Hypothetical protein	5.281	2.400856965	2.35090293815E-6	3.53443673422E-6	yes	up
PAS_chr1-4_0133	AAA-peroxin that heterodimerizes with AAA-peroxin Pex1p	6.039	2.594309657	1.72658040509E-158	2.18714681541E-157	yes	up
PAS_chr1-3_0176	Hypothetical protein	4.829	2.271777015	5.25416025219E-5	7.40767364963E-5	yes	up
PAS_chr1-1_0492	uncharacterized protein	5.614	2.489084148	4.33731292948E-34	1.57149472063E-33	yes	up
PAS_chr2-1_0318	Hypothetical protein	4.073	2.026239257	2.53140684644E-24	7.3416638009E-24	yes	up
PAS_chr3_0084	Hypothetical protein	5.288	2.402757749	8.18435013172E-116	8.13420885621E-115	yes	up
PAS_chr3_0822	Subunit of a heterodimeric peroxisomal ATP-binding cassette transporter complex (Pxa1p-Pxa2p)	11.218	3.487694134	0	0	yes	up
PAS_chr3_0680	Hypothetical protein	3.924	1.972383106	1.24402592617E-29	4.10512229837E-29	yes	up
PAS_chr1-1_0212	Methionyl-tRNA formyltransferase, catalyzes the formylation of initiator Met-tRNA in mitochondria	3.854	1.946291101	1.46135565109E-23	4.17091803026E-23	yes	up
PAS_chr4_0761	Major component of the proteasome	3.82	1.933534819	8.86710227276E-134	1.01577807129E-132	yes	up
PAS_chr1-3_0261	Ubiquitin isopeptidase	4.676	2.225267051	6.23179296515E-72	4.08600871209E-71	yes	up
PAS_chr2-2_0272	Subunit of a heterodimeric peroxisomal ATP-binding cassette transporter complex (Pxa1p-Pxa2p)	8.108	3.019368801	1.31498976075E-190	1.85240434364E-189	yes	up
PAS_chr4_0425	Hypothetical protein	5.037	2.332659258	1.70755563116E-32	6.00510298539E-32	yes	up
PAS_chr2-1_0348	Hypothetical protein	3.24	1.695831388	1.48213670129E-10	2.71239645953E-10	yes	up
PAS_chr2-1_0530	Ornithine transporter of the mitochondrial inner membrane	3.53	1.81978838	2.73783746302E-21	7.2580836065E-21	yes	up
PAS_chr1-1_0355	glycosyl hydrolase family 88, putative	7.531	2.912808908	1.59196018633E-83	1.19492056374E-82	yes	up
PAS_chr2-1_0534	Hypothetical protein	2.969	1.570187198	2.90456619599E-81	2.13241801454E-80	yes	up
PAS_chr4_0342	Acid trehalase required for utilization of extracellular trehalose	4.28	2.097664059	5.68853529008E-111	5.41811438898E-110	yes	up
PAS_chr1-1_0318	Hypothetical protein	3.307	1.725573655	1.10423772905E-78	7.87689580056E-78	yes	up
PAS_chr4_0160	Kinesin-related motor protein required for mitotic spindle assembly and chromosome segregation	2.997	1.583721022	3.92707822915E-49	1.85091625252E-48	yes	up
PAS_chr4_0532	Permease of basic amino acids in the vacuolar membrane	4.658	2.219600491	6.84276211905E-76	4.73997943481E-75	yes	up
PAS_chr4_0586	Hypothetical protein	10.615	3.407981399	9.27168517313E-202	1.38771740285E-200	yes	up
PAS_chr3_0992	Protein involved in pre-mRNA splicing	3.258	1.703946554	1.11379003911E-78	7.93378202083E-78	yes	up
PAS_chr1-1_0356	Hypothetical protein	11.394	3.510164415	6.3996405304E-126	6.90639318184E-125	yes	up
PAS_chr3_0436	uncharacterized protein	6.697	2.74344471	1.07524203578E-210	1.65870926317E-209	yes	up
PAS_chr4_0123	Receptor for alpha-factor pheromone	3.171	1.665021124	1.45533829374E-19	3.65761932994E-19	yes	up
PAS_chr2-2_0037	AP-1 accessory protein	2.996	1.582872627	1.54910731876E-130	1.72737487939E-129	yes	up
PAS_chr2-1_0458	Peroxin 20	6.478	2.695613902	6.89462832205E-62	3.95811482096E-61	yes	up
PAS_chr4_0751	Subunit of the telomeric Ku complex (Yku70p-Yku80p), involved in telomere length maintenance	3.326	1.733995301	8.5982357212E-50	4.09474691685E-49	yes	up
PAS_chr4_0611	Hypothetical protein	5.687	2.507772168	7.63132057363E-12	1.48406462354E-11	yes	up
PAS_chr1-3_0055	Class II abasic (AP) endonuclease involved in repair of DNA damage	2.848	1.50972044	6.14972133847E-26	1.87209132029E-25	yes	up
PAS_chr1-3_0078	S-adenosyl-L-methionine uroporphyrinogen III transmethylase	2.667	1.415182561	5.05624248685E-24	1.45634842304E-23	yes	up
PAS_chr2-2_0184	Single-stranded DNA endonuclease (with Rad10p)	2.894	1.533231251	1.28991873363E-54	6.70144763578E-54	yes	up
PAS_chr3_1149	uncharacterized protein	3.696	1.886011096	4.78178902246E-53	2.40235934005E-52	yes	up
PAS_chr3_0172	uncharacterized protein	2.536	1.342712922	2.58057380798E-19	6.42778884613E-19	yes	up
PAS_chr3_0497	Hypothetical protein	3.119	1.641206516	1.94029393968E-32	6.80930790136E-32	yes	up
PAS_chr3_0532	Intermediate filament protein	2.879	1.525788218	6.66645965168E-58	3.6204779253E-57	yes	up
PAS_chr4_0215	SH3 domain protein implicated in the regulation of actin polymerization	3.428	1.777544291	1.0454060251E-48	4.89510884564E-48	yes	up
PAS_chr1-4_0402	Hypothetical protein	6.315	2.658683079	5.12172341526E-25	1.51334589044E-24	yes	up
PAS_chr2-1_0249	Hypothetical protein, peroxisomal 2,4-dienoyl-CoA reductase	14.893	3.896546909	2.50384163023E-164	3.24531431918E-163	yes	up
PAS_chr3_0519	Permease of basic amino acids in the vacuolar membrane	3.526	1.817842736	2.54762114978E-47	1.16261222888E-46	yes	up
PAS_chr3_0246	Palmitoyltransferase with autoacylation activity	2.589	1.372489046	1.85833445437E-16	4.24605359882E-16	yes	up
PAS_chr2-2_0246	Subunit of the HIR complex, a nucleosome assembly complex involved in histone gene transcription	3.58	1.839901756	1.30028904335E-72	8.63824781905E-72	yes	up
PAS_chr4_0863	Hypothetical protein	2.32	1.213901203	7.70294757697E-22	2.08943491718E-21	yes	up
PAS_chr1-3_0148	Subunit of the Anaphase-Promoting Complex/Cyclosome (APC/C)	3.182	1.669877665	1.24965443408E-52	6.22228925642E-52	yes	up
PAS_chr1-4_0451	protein kinase	2.269	1.182204662	1.10124691173E-14	2.38098483194E-14	yes	up
PAS_FragB_0026	Hypothetical protein	5.409	2.435398275	1.06948391597E-22	2.97644416902E-22	yes	up
PAS_chr1-3_0212	Lanosterol 14-alpha-demethylase	2.636	1.398455474	2.27416489529E-23	6.45052186035E-23	yes	up
PAS_chr3_0283	Putative amidase	3.33	1.735724115	7.01159714912E-46	3.11771194191E-45	yes	up
PAS_chr3_0756	Mitochondrial membrane transporter	4.196	2.069147849	5.95815877781E-34	2.15410355813E-33	yes	up

PAS_chr4_0529	Hypothetical protein	5.527	2.466537482	5.16058316842E-37	1.97809243552E-36	yes	up
PAS_chr4_0993	uncharacterized protein	2.417	1.273380901	1.46514749519E-5	2.11730653831E-5	yes	up
PAS_chr1-3_0168	Peripheral membrane protein required for vesicle formation	3.449	1.786282271	1.58645886865E-183	2.21005585884E-182	yes	up
PAS_chr3_0326	Hypothetical protein	10.304	3.365080735	3.44096920627E-93	2.78657554562E-92	yes	up
PAS_chr3_0484	uncharacterized protein	3.735	1.901101285	4.67474105125E-87	3.63920630755E-86	yes	up
PAS_chr2-1_0317	Hypothetical protein	3.698	1.886912554	9.41369086663E-91	7.47890226987E-90	yes	up
PAS_chr1-3_0071	Subunit of the nuclear pore complex (NPC)	2.587	1.371333119	5.58984275842E-79	3.99876518238E-78	yes	up
PAS_chr3_0101	Hypothetical protein	2.159	1.110266836	7.92443085561E-7	1.22546010987E-6	yes	up
PAS_chr2-2_0270	Essential structural subunit of the nuclear pore complex (NPC)	2.992	1.581046887	4.69140288199E-124	4.96696107232E-123	yes	up
PAS_chr4_0499	uncharacterized protein	3.236	1.694279397	1.68707105218E-93	1.3684323099E-92	yes	up
PAS_chr3_0348	Hypothetical protein	13.091	3.710535783	1.42003851844E-306	2.89124441669E-305	yes	up
PAS_chr1-4_0564	Iron-sulfur protein IND1	2.439	1.286040473	1.43415404587E-9	2.53331952816E-9	yes	up
PAS_chr3_0476	uncharacterized protein	2.961	1.566126962	2.77606669922E-38	1.09154334874E-37	yes	up
PAS_chr3_1196	uncharacterized protein	5.618	2.490006587	1.30839886404E-42	5.54800833666E-42	yes	up
PAS_chr4_0204	uncharacterized protein	5.433	2.441702017	4.67584280852E-60	2.62149537169E-59	yes	up
PAS_chr1-4_0227	uncharacterized protein	2.433	1.282551487	5.00614542462E-14	1.05781114876E-13	yes	up
PAS_chr3_0598	uncharacterized protein	2.659	1.411018936	1.06590019865E-12	2.14674092872E-12	yes	up
PAS_chr3_0713	Subunit of the SAGA transcriptional regulatory complex, involved in proper assembly of the complex	3.747	1.905649117	1.63026350716E-147	1.94740978088E-146	yes	up
PAS_chr1-1_0156	Hypothetical protein	2.037	1.026414864	3.5262880591E-26	1.07869237525E-25	yes	up
PAS_chr3_0530	Hypothetical protein	2.256	1.173845228	4.04247753514E-15	8.85050915291E-15	yes	up
PAS_chr4_0722	Hypothetical protein	2.054	1.038286885	0.000256131007249	0.000346258826735	yes	up
PAS_chr4_0148	Gamma-tubulin	4.271	2.094618536	4.81357699187E-64	2.85129313217E-63	yes	up
PAS_chr1-4_0261	Hypothetical protein	3.52	1.815594496	8.42819438074E-23	2.35082582034E-22	yes	up
PAS_chr1-3_0131	Hypothetical protein	2.713	1.439885249	2.86406545353E-26	8.77719997916E-26	yes	up
PAS_chr1-4_0380	Phosphatidylinositol 4-kinase	2.313	1.210067443	5.22130352278E-41	2.1522897882E-40	yes	up
PAS_chr2-1_0025	Non-essential protein of unknown function, contains ATP/GTP-binding site motif A	3.042	1.605039743	5.76780787753E-123	6.05559620378E-122	yes	up
PAS_chr3_0327	Putative membrane glycoprotein with strong similarity to Vth2p and Pep1p/Vps10p, may be involved in	8.057	3.010293551	1.46195580016E-148	1.75469587565E-147	yes	up
PAS_chr3_0922	Polyamine oxidase, converts spermine to spermidine	2.232	1.158629858	6.54913472572E-22	1.78126546975E-21	yes	up
PAS_chr1-1_0386	DNA 3'-phosphatase that functions in repair of endogenous damage of double-stranded DNA	2.195	1.133918104	4.12488596314E-7	6.46232134225E-7	yes	up
PAS_chr2-2_0214	uncharacterized protein	4.299	2.104111521	2.00272543442E-49	9.46588929481E-49	yes	up
PAS_chr1-4_0165	Class E protein of the vacuolar protein-sorting (Vps) pathway	2.065	1.046064236	2.99243257931E-10	5.42108913593E-10	yes	up
PAS_chr3_1207	uncharacterized protein	2.394	1.259522753	3.49127325853E-22	9.58908422565E-22	yes	up
PAS_chr2-2_0466	uncharacterized protein	2.39	1.257003325	2.8464992377E-24	8.24599347143E-24	yes	up
PAS_chr3_0018	High affinity methionine permease, integral membrane protein with 13 putative membrane-spanning regi	2.304	1.203872951	1.75675075735E-22	4.86224521669E-22	yes	up
PAS_chr2-1_0190	Putative ABC transporter	2.791	1.480872614	1.74065977115E-85	1.33238629971E-84	yes	up
PAS_chr4_0045	Hypothetical protein	2.587	1.371417418	9.49936158573E-17	2.19138942269E-16	yes	up
PAS_chr1-1_0383	Component of U4/U6.U5 snRNP involved in mRNA splicing via spliceosome	2.248	1.168683522	9.1245125344E-6	1.33315437349E-5	yes	up
PAS_chr1-1_0052	Hypothetical protein	3.847	1.943616967	2.16441297584E-48	1.00785489403E-47	yes	up
PAS_chr2-2_0249	Cell-wall L-asparaginase II, involved in asparagine catabolism	4.284	2.098805698	1.04429374242E-46	4.7313092168E-46	yes	up
PAS_chr1-3_0167	Protein with a role in 5'-end processing of mitochondrial RNAs	2.352	1.233696196	8.91284419782E-30	2.95664204953E-29	yes	up
PAS_chr2-1_0851	uncharacterized protein	6.366	2.670336664	4.68823210698E-159	5.96889095341E-158	yes	up
PAS_chr4_0326	Transcriptional activator that enhances pseudohyphal growth	2.238	1.162257253	1.39674998479E-17	3.30864610151E-17	yes	up
PAS_chr1-1_0068	uncharacterized protein	2.794	1.482275758	5.06561656563E-12	9.95894671952E-12	yes	up
PAS_chr3_0851	Subunit of a complex with Rad50p and Xrs2p (MRX complex)	2.086	1.060787718	4.37082793009E-22	1.19591369208E-21	yes	up
PAS_chr3_0071	Hypothetical protein	3.705	1.889595077	9.94723714483E-91	7.89032422734E-90	yes	up
PAS_chr1-1_0325	Hypothetical protein	4.069	2.02475373	1.38541217419E-25	4.16950198922E-25	yes	up
PAS_chr3_0905	Putative transcription factor, has homolog in Kluyveromyces lactis	2.345	1.229454246	8.20310743883E-38	3.20042104809E-37	yes	up
PAS_chr2-1_0726	uncharacterized protein	2.455	1.295890704	1.58500164719E-32	5.57800789624E-32	yes	up
PAS_chr3_0270	Coenzyme Q (ubiquinone) binding protein	2.498	1.320686616	5.2346395469E-7	8.16785674259E-7	yes	up
PAS_chr2-1_0724	Hypothetical protein	2.541	1.345561285	1.61635469141E-8	2.7068424053E-8	yes	up
PAS_chr1-4_0460	Uridine kinase (Uridine monophosphokinase)	2.545	1.347486675	2.47825988499E-11	4.71375528049E-11	yes	up
PAS_chr4_0673	uncharacterized protein	2.141	1.098294195	5.06137615719E-8	8.29649957448E-8	yes	up
PAS_chr3_0374	Hypothetical protein	5.218	2.38356189	2.97441333274E-151	3.60441557839E-150	yes	up
PAS_chr2-1_0883	uncharacterized protein	2.269	1.182204662	4.66085489429E-8	7.64745163569E-8	yes	up
PAS_chr3_1038	Hypothetical protein	3.185	1.671183863	5.04408805874E-31	1.71977754898E-30	yes	up
PAS_chr3_0043	peroxisomal integral membrane protein	4.292	2.101770189	1.0941626127E-74	7.42583505975E-74	yes	up
PAS_chr3_0120	Integral membrane protein of the endoplasmic reticulum, required for normal content of cell wall bet	2.36	1.238809609	5.18078790719E-16	1.16782529741E-15	yes	up
PAS_chr4_0614	Hypothetical protein	3.266	1.707709739	1.98982903324E-66	1.21737837082E-65	yes	up
PAS_chr4_0636	uncharacterized protein	2.392	1.257941943	5.72564654535E-94	4.65174094937E-93	yes	up
PAS_chr2-1_0440	Hypothetical protein	2.08	1.056921547	1.91521747801E-28	6.19397343853E-28	yes	up
PAS_chr1-4_0322	uncharacterized protein	3.054	1.610733973	6.79555296638E-48	3.14395914149E-47	yes	up

PAS_chr4_0334	Transcription factor involved in glucose repression	4.54	2.182602024	4.94649342089E-81	3.62094838627E-80	yes	up
PAS_chr3_0378	Peptidyl-prolyl cis-trans isomerase (cyclophilin)	2.29	1.195559228	2.01820717611E-16	4.60715564624E-16	yes	up
PAS_chr2-1_0408	Putative transporter (DHA1 family) of multidrug resistance transporter	2.792	1.481223948	6.04160658026E-42	2.53193662434E-41	yes	up
PAS_chr2-2_0455	uncharacterized protein	2.541	1.345604248	3.2824083614E-45	1.44800277627E-44	yes	up
PAS_chr3_0369	Protein involved in the inositol acylation of glucosaminyl phosphatidylinositol (GlcN-PI)	3.259	1.704645804	4.12328624446E-52	2.03095068789E-51	yes	up
PAS_chr4_0741	Alpha subunit of Type II geranylgeranyltransferase	2.778	1.473957455	5.6383700087E-19	1.39544108139E-18	yes	up
PAS_chr1-1_0368	One of six subunits of the RNA polymerase III transcription initiation factor complex (TFIIIC)	2.202	1.138902531	7.70327733602E-42	3.22026448236E-41	yes	up
PAS_chr4_0310	uncharacterized protein	2.238	1.16198887	8.70692561667E-12	1.6899702403E-11	yes	up
PAS_chr4_0079	Hypothetical protein	2.415	1.271777015	7.26363326462E-8	1.17872899928E-7	yes	up
PAS_chr2-1_0345	Subunit of the RAVE complex (Rav1p, Rav2p, Skp1p), which promotes assembly of the V-ATPase holoenzym	2.035	1.025323582	3.30230710219E-46	1.48411996576E-45	yes	up
PAS_chr3_0669	Hypothetical protein	3	1.584969568	1.4834695936E-89	1.16750682101E-88	yes	up
PAS_chr4_0143	Swi2/Snf2-related ATPase that is the structural component of the SWR1 complex	2.789	1.479622592	2.23449792839E-127	2.43759003945E-126	yes	up
PAS_chr2-1_0870	uncharacterized protein	3.034	1.601279393	3.82843273967E-59	2.12507596554E-58	yes	up
PAS_chr4_0447	Hypothetical protein	3.627	1.858580863	6.4287170162E-11	1.20141277869E-10	yes	up
PAS_chr2-1_0261	Activator of Chs3p (chitin synthase III), recruits Chs3p to the bud neck via interaction with Bni4p	4.442	2.151269516	1.32882770694E-110	1.26326550817E-109	yes	up
PAS_chr2-1_0531	Hypothetical protein	2.811	1.491056264	1.5950415043E-156	2.00036501873E-155	yes	up
PAS_chr4_0752	Hypothetical protein	2.912	1.541790341	5.69234113333E-29	1.85286624981E-28	yes	up
PAS_chr2-1_0873	uncharacterized protein	3.016	1.592438239	2.35790533676E-14	5.03093166676E-14	yes	up
PAS_chr3_1218	uncharacterized protein	3.02	1.594609338	8.15248596443E-12	1.58419056859E-11	yes	up
PAS_chr3_1171	uncharacterized protein	2.419	1.274392964	7.54604485527E-22	2.04908529034E-21	yes	up
PAS_chr2-1_0040	Protein with similarity to ATP-binding cassette (ABC) transporter family members	2.335	1.223353531	3.75122649497E-24	1.08419069214E-23	yes	up
PAS_chr2-1_0441	Hypothetical protein	2.162	1.112415303	2.40677677129E-7	3.80858413556E-7	yes	up
PAS_chr2-1_0650	Hypothetical protein	2.706	1.436163833	9.69764997278E-6	1.41524903404E-5	yes	up
PAS_chr3_1039	Subunit of the Nup84p subcomplex of the nuclear pore complex (NPC)	2.348	1.231317968	1.12028013228E-38	4.45366702392E-38	yes	up
PAS_chr3_0273	Non-essential hydrolase involved in mRNA decapping	2.07	1.04989737	1.19682184547E-9	2.12303952765E-9	yes	up
PAS_chr1-1_0077	uncharacterized protein	3.029	1.598962964	6.74649673082E-97	5.59870165995E-96	yes	up
PAS_chr1-3_0185	Delta 2-isopentenyl pyrophosphate:tRNA isopentenyl transferase	2.37	1.244832861	3.11428843378E-22	8.55833690353E-22	yes	up
PAS_chr4_0723	Hypothetical protein	2.593	1.374625423	8.06985091255E-22	2.1854216607E-21	yes	up
PAS_chr1-4_0215	Acetylglutamate synthase (glutamate N-acetyltransferase), mitochondrial enzyme	3.909	1.9666259	1.01564037612E-92	8.19848387078E-92	yes	up
PAS_chr3_0496	RNA-dependent ATPase in the DEAH-box family	2.16	1.111183706	2.10927025283E-36	8.00567554829E-36	yes	up
PAS_chr4_0632	Hypothetical protein	3.177	1.667785286	9.14595192023E-41	3.75163068571E-40	yes	up
PAS_chr2-1_0447	uncharacterized protein	2.334	1.222860297	1.65442920936E-59	9.21386987137E-59	yes	up
PAS_chr2-1_0398	DNA repair protein Nse1	2.095	1.067238259	9.22585281647E-8	1.48850862413E-7	yes	up
PAS_chr1-4_0690	uncharacterized protein	2.395	1.259883337	1.07081435173E-9	1.90219900207E-9	yes	up
PAS_chr1-3_0018	Hypothetical protein	4.745	2.246432061	8.2094919419E-174	1.10389130951E-172	yes	up
PAS_chr4_0339	Hypothetical protein	11.317	3.500465518	1.59168478185E-232	2.65932982322E-231	yes	up
PAS_chr4_0132	uncharacterized protein	2.164	1.113797029	6.01572289255E-26	1.83240886897E-25	yes	up
PAS_chr3_0490	Zinc-finger transcription factor of the Zn(2)-Cys(6) binuclear cluster domain type	2.562	1.357059006	7.0514547735E-60	3.94897172115E-59	yes	up
PAS_chr2-2_0095	Rho GTPase activating protein (RhoGAP)	2.107	1.074874372	1.21010262555E-79	8.73114218637E-79	yes	up
PAS_chr2-1_0501	Plasma membrane protein that may be involved in osmotolerance	2.295	1.198378382	1.95407576948E-44	8.50090574802E-44	yes	up
PAS_chr1-1_0178	Hypothetical protein	2.431	1.281536862	9.5147923267E-19	2.3375618276E-18	yes	up
PAS_FragB_0038	GTPase-activating protein	2.622	1.390859209	7.67679493564E-54	3.9234351353E-53	yes	up
PAS_chr3_1076	Glycerol proton symporter of the plasma membrane	9.929	3.311641476	0	0	yes	up
PAS_chr2-2_0307	Hypothetical protein	2.249	1.16908522	1.18696998669E-44	5.18616165339E-44	yes	up
PAS_chr4_0137	uncharacterized protein	2.477	1.308335513	7.4750986976E-45	3.28316780351E-44	yes	up
PAS_chr1-1_0007	SRR1 domain-containing protein	2.237	1.161488021	1.19868588924E-8	2.02152626994E-8	yes	up
PAS_chr1-1_0483	uncharacterized protein	2.74	1.454002343	3.18908432002E-128	3.50938841256E-127	yes	up
PAS_chr1-1_0228	Hypothetical protein	3.492	1.804221384	1.56039140743E-36	5.94485483937E-36	yes	up
PAS_chr2-2_0286	Hypothetical protein	2.833	1.502571954	3.9994166666E-66	2.42911430149E-65	yes	up
PAS_chr2-1_0511	Homodimeric Zn2Cys6 zinc finger transcription factor	2.289	1.194610777	6.39488026245E-40	2.59144664302E-39	yes	up
PAS_chr1-4_0118	DEAH-box RNA-dependent ATPase/ATP-dependent RNA helicase	2.087	1.061319859	2.60666483118E-39	1.04370361752E-38	yes	up
PAS_chr2-1_0240	Hypothetical protein	2.833	1.502470701	1.48295494756E-57	8.01050529674E-57	yes	up
PAS_chr2-2_0276	Plasma membrane sulfite pump involved in sulfite metabolism	8.992	3.168683522	6.60815876878E-246	1.16197309259E-244	yes	up
PAS_chr4_0207	Hypothetical protein	2.525	1.33633988	9.0949094478E-10	1.62192551819E-9	yes	up
PAS_chr4_0256	Essential protein required for the accumulation of box C/D snoRNA	3.006	1.587625476	6.21100193332E-30	2.06718257595E-29	yes	up
PAS_chr2-1_0815	uncharacterized protein	3.286	1.71635879	6.09370893811E-27	1.89168285492E-26	yes	up
PAS_chr3_1222	uncharacterized protein	3.057	1.61214943	1.1499962973E-49	5.46112500387E-49	yes	up
PAS_chr3_0925	uncharacterized protein	3.325	1.733502875	1.41202529358E-169	1.87363461779E-168	yes	up
PAS_chr4_0480	Hypothetical protein	2.826	1.498604408	2.533739165E-14	5.39237167194E-14	yes	up
PAS_chr2-2_0143	Component of the Trk1p-Trk2p potassium transport system	2.436	1.28443073	2.93841686672E-57	1.58045972436E-56	yes	up
PAS_chr1-4_0627	Putative serine/threonine kinase	2.682	1.423382069	6.46865336647E-41	2.65558022694E-40	yes	up

PAS_chr4_0633	uncharacterized protein	2.422	1.276012877	3.45685971306E-53	1.74368580712E-52	yes	up
PAS_chr1-4_0538	Fatty-acyl coenzyme A oxidase	14.266	3.834495244	0	0	yes	up
PAS_chr2-1_0349	Mitochondrial integral inner membrane protein required for membrane insertion of C-terminus of Cox2p	2.528	1.338143813	7.26100615718E-19	1.7934970513E-18	yes	up
PAS_chr2-1_0078	Plasma membrane G protein coupled receptor (GPCR) that interacts with the heterotrimeric G protein a	2.793	1.481762686	4.21122431497E-59	2.33240606607E-58	yes	up
PAS_chr1-3_0181	uncharacterized protein	2.478	1.309133012	1.80921609484E-97	1.51138666793E-96	yes	up
PAS_chr2-1_0503	Hypothetical protein	3.17	1.664641017	4.08040730807E-15	8.929664209E-15	yes	up
PAS_chr1-1_0229	Hypothetical protein	2.551	1.351060265	4.83272814324E-16	1.09083437309E-15	yes	up
PAS_chr3_0803	Vacuolar aminopeptidase	3.232	1.692427281	7.30093184107E-64	4.3144989693E-63	yes	up
PAS_chr3_0275	uncharacterized protein	2.387	1.255098274	4.41443143607E-20	1.12920527426E-19	yes	up
PAS_chr4_0511	Mitochondrial beta-keto-acyl synthase with possible role in fatty acid synthesis	3.285	1.715686362	1.91665011242E-50	9.26810905325E-50	yes	up
PAS_chr1-3_0109	Putative oxidoreductase	2.152	1.105380941	3.12258966766E-18	7.56066607542E-18	yes	up
PAS_FragB_0020	Vacuolar protein sorting-associated protein 70	3.083	1.624521553	1.28891588527E-83	9.68902539168E-83	yes	up
PAS_chr1-1_0387	Hypothetical protein	2.57	1.361579047	1.22962154821E-51	6.02119451406E-51	yes	up
PAS_chr3_0993	Subunit of the Nup84p subcomplex of the nuclear pore complex (NPC)	2.037	1.026592729	2.75746598242E-44	1.19545658841E-43	yes	up
PAS_chr4_0555	Single-stranded DNA endonuclease, cleaves single-stranded DNA during nucleotide excision repair	2.178	1.122677245	4.51102841114E-49	2.12216668659E-48	yes	up
PAS_chr2-1_0391	Lanosterol synthase, an essential enzyme that catalyzes the cyclization of squalene 2,3-epoxide	2.558	1.355189463	3.88761296126E-50	1.87089048633E-49	yes	up
PAS_chr1-4_0470	Membrane protein involved in the synthesis of N-acetylglucosaminyl phosphatidylinositol (GlcNAc-PI)	2.334	1.222654533	1.4278385616E-34	5.24131396081E-34	yes	up
PAS_chr4_0268	Hypothetical protein	19.441	4.281037539	0	0	yes	up
PAS_chr2-1_0807	uncharacterized protein	2.86	1.515773338	7.12348153864E-164	9.20925158299E-163	yes	up
PAS_chr2-1_0189	Protein required for respiratory growth and stability of the mitochondrial genome	6.971	2.801367731	3.21458044436E-96	2.65453613378E-95	yes	up
PAS_chr4_0186	Plasma membrane ATP-binding cassette (ABC) transporter	3.18	1.669112513	2.27342291938E-172	3.04070315467E-171	yes	up
PAS_chr1-1_0360	2-methylbutyraldehyde reductase, may be involved in isoleucine catabolism	2.714	1.440674404	5.58980006858E-22	1.5236371027E-21	yes	up
PAS_chr4_0531	uncharacterized protein	2.11	1.07727573	8.0557226681E-28	2.55759023345E-27	yes	up
PAS_chr3_0512	Mg<sup&gt2+</sup&gt-dependent phosphatidate (PA) phosphatase	2.04	1.02867034	4.00883682871E-30	1.34223970783E-29	yes	up
PAS_chr1-4_0080	Fatty acid transporter and very long-chain fatty acyl-CoA synthetase	2.183	1.125993709	1.90864054415E-29	6.2735642461E-29	yes	up
PAS_chr2-2_0188	Protein involved in pre-mRNA splicing, component of a complex containing Cef1p	2.196	1.135130963	2.95572213579E-13	6.07202884841E-13	yes	up
PAS_chr2-1_0854	uncharacterized protein	2.179	1.123771101	5.62922004903E-13	1.14612743427E-12	yes	up
PAS_chr2-1_0119	Nuclear protein that acts as a heterodimer with Aos1p to activate Smt3p (SUMO) before its conjugatio	2.679	1.421860295	1.83013532106E-40	7.46451786667E-40	yes	up
PAS_chr1-1_0069	uncharacterized protein	3.116	1.639646436	1.02496563174E-64	6.10006173019E-64	yes	up
PAS_chr3_0156	Diaminohydroxyphoshoribosylaminopyrimidine deaminase	2.323	1.215989237	4.62513382895E-11	8.70501423122E-11	yes	up
PAS_chr1-4_0323	tRNAHis guanylyltransferase	2.019	1.013865413	7.69711838161E-9	1.31171834433E-8	yes	up
PAS_chr3_0894	Zinc-finger protein of unknown function	5.152	2.365169052	2.00986293088E-133	2.28162543553E-132	yes	up
PAS_chr2-2_0460	uncharacterized protein	2.188	1.129689391	1.63142066447E-11	3.12311173264E-11	yes	up
PAS_chr2-2_0040	Cell-cycle checkpoint serine-threonine kinase	2.075	1.053449163	3.77827926834E-20	9.69931926518E-20	yes	up
PAS_chr3_0924	Ubiquitin conjugating enzyme, involved in the ER-associated protein degradation pathway	2.348	1.231419278	2.28733268149E-6	3.44297996266E-6	yes	up
PAS_chr2-1_0304	Kex2 proprotein convertase	2.126	1.088301006	5.35899182386E-35	1.97728319018E-34	yes	up
PAS_chr4_0214	Glucose-repressible ADP-ribosylation factor	3.418	1.773198821	3.1883495025E-13	6.54190520117E-13	yes	up
PAS_chr1-4_0398	Hypothetical protein	2.757	1.463264697	8.73322104325E-26	2.64893658785E-25	yes	up
PAS_chr1-1_0175	Hypothetical protein	2.715	1.441116626	6.20752566229E-17	1.43926447928E-16	yes	up
PAS_chr4_0612	Peripherally bound inner membrane protein of the mitochondrial matrix	2.11	1.077291924	2.70474860702E-14	5.74901975684E-14	yes	up
PAS_chr2-1_0623	Hypothetical protein	2.613	1.385647782	2.33162167084E-53	1.18322153205E-52	yes	up
PAS_chr1-1_0145	Cytoplasmic protein with a role in regulation of Ty1 transposition	2.693	1.42943797	1.92666248323E-92	1.55026970051E-91	yes	up
PAS_chr2-1_0662	Large subunit of the nuclear mRNA cap-binding protein complex	2.306	1.205424069	1.3217506154E-53	6.73463408801E-53	yes	up
PAS_chr2-1_0178	uncharacterized protein	2.619	1.389053946	8.73543998428E-91	6.95103286091E-90	yes	up
PAS_chr4_0571	Glycerol proton symporter of the plasma membrane, subject to glucose-induced inactivation	7.134	2.834665965	1.4984373885E-274	2.83294797999E-273	yes	up
PAS_chr1-4_0626	GTP-binding protein YPT10	2.534	1.341445176	5.49424309384E-12	1.07805495587E-11	yes	up
PAS_chr4_0341	Hypothetical protein	4.798	2.262289637	7.17630339227E-73	4.79277951656E-72	yes	up
PAS_chr2-1_0343	Hypothetical protein	2.669	1.416166925	6.13820491156E-41	2.52197977943E-40	yes	up
PAS_chr1-4_0349	Peroxisomal biogenesis factor 8	3.074	1.620349944	3.27443926755E-86	2.51406947733E-85	yes	up
PAS_chr3_0886	Hypothetical protein	2.362	1.23976662	1.50360180746E-5	2.17162937672E-5	yes	up
PAS_chr1-4_0078	Multidrug transporter of the major facilitator superfamily	2.083	1.058523666	8.13190685367E-30	2.70114660285E-29	yes	up
PAS_chr4_0647	Low affinity vacuolar membrane localized monovalent cation/H+ antiporter	2.328	1.219319414	9.96013573608E-69	6.29265359507E-68	yes	up
PAS_chr1-1_0393	Chitin synthase I	3.042	1.605109076	2.4374241474E-117	2.46140683479E-116	yes	up
PAS_chr1-4_0443	Hypothetical protein	2.06	1.042725365	7.31491658469E-27	2.26658752338E-26	yes	up
PAS_chr4_0528	mRNA transport regulator, essential nuclear protein	2.1	1.070549204	9.7525527646E-6	1.42284850169E-5	yes	up
PAS_chr2-2_0315	uncharacterized protein	2.094	1.06598057	5.93498210754E-28	1.89384676515E-27	yes	up
PAS_chr3_0865	Protein with a potential role in actin cytoskeletal organization	2.174	1.120658574	1.26450580122E-43	5.43057188244E-43	yes	up
PAS_chr3_0148	Glucose-repressible protein kinase involved in signal transduction during cell proliferation in resp	2.157	1.108905109	1.10912624707E-84	8.40029502484E-84	yes	up
PAS_chr2-1_0214	Mitochondrial protein required for assembly of ubiquinol cytochrome-c reductase complex (cytochrome	2.039	1.027709895	1.94738731898E-54	1.00859019847E-53	yes	up
PAS_chr3_1212	uncharacterized protein	2.53	1.339182959	7.04686682238E-11	1.31060256101E-10	yes	up
PAS_chr3_0833	Amphiphysin-like lipid raft protein	3.324	1.733024966	1.76475614374E-32	6.19759682044E-32	yes	up

PAS_chr3_0653	uncharacterized protein	2.805	1.488215929	1.94377548465E-160	2.49368543681E-159	yes	up
PAS_chr1-4_0566	Low-affinity cyclic AMP phosphodiesterase	3.063	1.615054229	9.55356538524E-42	3.98712699771E-41	yes	up
PAS_chr2-1_0027	uncharacterized protein	2.906	1.53889124	1.37559533107E-45	6.07897093144E-45	yes	up
PAS_chr3_0220	Mitogen-activated protein (MAP) kinase kinase kinase	2.001	1.000693726	4.17801541112E-57	2.24000421136E-56	yes	up
PAS_chr3_0590	Homeodomain-containing transcriptional repressor of PTR2	2.135	1.093915754	1.19629439547E-9	2.12285268694E-9	yes	up
PAS_chr4_0487	Carbon source-responsive zinc-finger transcription factor	10.261	3.359032165	0	0	yes	up
PAS_chr4_0013	Hypothetical protein	2.641	1.400946188	7.91625128409E-75	5.3944210986E-74	yes	up
PAS_chr4_0083	Splicing factor that is found in the Cef1p subcomplex of the spliceosome	2.42	1.274838813	1.01408849495E-29	3.35958566609E-29	yes	up
PAS_chr2-2_0472	uncharacterized protein	2.439	1.286106384	2.62978294575E-22	7.24667311462E-22	yes	up
PAS_chr3_1139	Sorting nexin	2.172	1.118814273	3.85102516993E-25	1.14056569962E-24	yes	up
PAS_chr4_0058	Hypothetical protein	2.704	1.434857636	6.23766277663E-32	2.15892677933E-31	yes	up
PAS_chr2-1_0750	uncharacterized protein	2.852	1.511897841	2.54680807593E-32	8.91914889545E-32	yes	up
PAS_chr2-1_0581	uncharacterized protein	2.365	1.242069406	1.03662735754E-40	4.2487359259E-40	yes	up
PAS_chr1-4_0515	Protein involved in nitrosoguanidine (MNNG) resistance	2.169	1.116990288	8.79208539406E-33	3.10719588522E-32	yes	up
PAS_chr1-1_0418	Acetate transporter required for normal sporulation	34.574	5.111623778	0	0	yes	up
PAS_chr3_0325	Essential splicesome assembly factor	3.32	1.731296815	1.71338446164E-104	1.54143299778E-103	yes	up
PAS_chr1-1_0116	Component of the conserved oligomeric Golgi complex (Cog1p through Cog8p)	2.077	1.054467954	2.67990945464E-12	5.31019095642E-12	yes	up
PAS_chr1-3_0088	Hypothetical protein	2.525	1.336179782	1.24393605816E-59	6.95083826276E-59	yes	up
PAS_chr1-4_0555	Adapter protein for pexophagy and the cytoplasm-to-vacuole targeting (Cvt) pathway	2.199	1.136969855	3.13211959871E-76	2.17561180413E-75	yes	up
PAS_chr3_0282	uncharacterized protein	2.419	1.274304365	1.18967833367E-48	5.56030886622E-48	yes	up
PAS_chr4_0824	Protein required for resistance to the antifungal drug ciclopirox olamine	2.498	1.320686616	2.37848222423E-39	9.54619880738E-39	yes	up
PAS_chr2-2_0160	Vacuolar amino acid transporter, exports aspartate and glutamate from the vacuole	2.689	1.427064069	4.80207399306E-42	2.01414763229E-41	yes	up
PAS_chr1-4_0283	Poly(A) binding protein, part of the 3'-end RNA-processing complex	2.488	1.314942633	2.55751854446E-23	7.24198240995E-23	yes	up
PAS_chr2-2_0302	Multifunctional enzyme of the folic acid biosynthesis pathway	2.727	1.44713979	2.35630366757E-77	1.65964301739E-76	yes	up
PAS_chr3_0429	Protein required for transport of flavin adenine dinucleotide (FAD)	2.075	1.053206305	1.59840009189E-12	3.19616463703E-12	yes	up
PAS_chr2-1_0090	tRNA methyltransferase, localizes to both the nucleus and mitochondrion to produce the modified base	2.599	1.378136888	7.11435320172E-43	3.02435183867E-42	yes	up
PAS_chr2-1_0204	RNAse	4.062	2.022365134	7.11259159214E-87	5.51994183902E-86	yes	up
PAS_chr1-4_0699	uncharacterized protein	2.399	1.262639331	4.30155423956E-20	1.10089141327E-19	yes	up
PAS_chr3_0729	Phosphotyrosine-specific protein phosphatase	2.088	1.061868565	3.92810946906E-12	7.74988721847E-12	yes	up
PAS_chr1-3_0183	RNA-binding subunit of the mRNA cleavage and polyadenylation factor	2.014	1.010346495	5.37583375678E-63	3.12544138299E-62	yes	up
PAS_chr4_0155	Hypothetical protein	2.167	1.11594206	8.71619622005E-8	1.40808707969E-7	yes	up
PAS_chr4_0351	Hypothetical protein	3.167	1.662891235	1.82827049467E-18	4.44817238399E-18	yes	up
PAS_chr2-2_0232	Haploid specific endoprotease	2.185	1.127848608	5.76962068804E-65	3.45010968373E-64	yes	up
PAS_chr1-4_0459	Malate synthase	3.189	1.67294234	1.54642727617E-56	8.22961139882E-56	yes	up
PAS_chr2-2_0176	Hypothetical protein	3.191	1.673838316	2.8696103351E-49	1.35504886152E-48	yes	up
PAS_chr3_0042	Myristoylated serine/threonine protein kinase involved in vacuolar protein sorting	2.778	1.474161097	1.86141133078E-150	2.25024941887E-149	yes	up
PAS_chr3_0730	Hypothetical protein	2.217	1.148334877	6.71960141156E-14	1.41392784513E-13	yes	up
PAS_chr3_1194	uncharacterized protein	2.274	1.185171645	1.64751196733E-27	5.18481707365E-27	yes	up
PAS_chr1-1_0092	Hypothetical protein	2.207	1.142302647	8.45649311097E-35	3.11102442246E-34	yes	up
PAS_chr2-1_0469	uncharacterized protein	2.144	1.100408597	2.92872919051E-48	1.35872500914E-47	yes	up
PAS_chr2-1_0301	Voltage-gated high-affinity calcium channel	2.292	1.196814958	2.62116703811E-145	3.11627636753E-144	yes	up
PAS_chr1-1_0091	Protein involved in programmed cell death	2.386	1.254506573	2.24508758501E-52	1.11236901133E-51	yes	up
PAS_chr2-2_0186	Peroxisomal membrane signal receptor for the C-terminal tripeptide signal sequence (PTS1)	16.113	4.010131012	0	0	yes	up
PAS_chr2-1_0562	uncharacterized protein	5.09	2.347680695	0	0	yes	up
PAS_chr1-4_0310	Autophagy-related protein	3.162	1.660665607	5.16631885355E-94	4.20411286643E-93	yes	up
PAS_chr3_0095	Phenylpyruvate decarboxylase, catalyzes decarboxylation of phenylpyruvate to phenylacetaldehyde, whi	4.88	2.286864949	4.55295920335E-200	6.75422767955E-199	yes	up
PAS_chr1-3_0276	Mucin family member	2.268	1.181411827	1.14943283291E-55	6.05287719025E-55	yes	up
PAS_chr1-4_0363	Cyclin-dependent kinase-activating kinase required for passage through the cell cycle	2.05	1.035416992	1.41113061005E-12	2.83183393374E-12	yes	up
PAS_chr2-1_0225	Zinc cluster transcriptional activator involved in conferring resistance to ketoconazole	2.272	1.184037769	1.12983128173E-31	3.9024186235E-31	yes	up
PAS_chr3_1079	Hypothetical protein	2.362	1.23976662	8.76858542409E-21	2.28957508296E-20	yes	up
PAS_chr3_0931	Phosphoinositide binding protein required for vesicle formation in autophagy	2.632	1.396278259	7.18821088377E-50	3.42974502225E-49	yes	up
PAS_chr2-2_0310	Dipeptidyl aminopeptidase, Golgi integral membrane protein	2.675	1.419672587	2.01267767043E-86	1.55003920438E-85	yes	up
PAS_chr1-4_0562	Bile pigment transporter	2.11	1.077246136	9.98312037274E-85	7.57241513642E-84	yes	up
PAS_chr2-1_0816	uncharacterized protein	2.126	1.088466534	2.97511586291E-46	1.33826991723E-45	yes	up
PAS_chr3_0259	uncharacterized protein	2.888	1.530315873	1.09291132132E-112	1.05697135287E-111	yes	up
PAS_chr4_0749	Golgi membrane protein involved in manganese homeostasis	2.829	1.500545539	2.28559198831E-25	6.82150867014E-25	yes	up
PAS_FragB_0063	Polyadenylation factor subunit 2	2.235	1.160070392	2.30616693562E-25	6.8788336413E-25	yes	up
PAS_chr4_0457	uncharacterized protein	2.149	1.103940758	2.28613899909E-46	1.03204605264E-45	yes	up
PAS_chr2-2_0209	uncharacterized protein	2.178	1.123303619	1.53725556988E-31	5.29148409372E-31	yes	up
PAS_chr2-1_0491	uncharacterized protein	2.104	1.073376562	2.47821911734E-27	7.77477476051E-27	yes	up
PAS_chr2-1_0499	uncharacterized protein	2.412	1.270060543	3.43870600639E-9	5.96730590274E-9	yes	up

PAS_chr1-3_0129	Hypothetical protein	2.712	1.439331112	8.74767719163E-27	2.70553927409E-26	yes	up
PAS_chr3_0036	S-adenosylmethionine transporter of the mitochondrial inner membrane, member of the mitochondrial ca	2.334	1.222839292	2.73793413504E-16	6.22753087521E-16	yes	up
PAS_chr1-4_0656	uncharacterized protein	2.758	1.463792846	1.30899531651E-97	1.09715624112E-96	yes	up
PAS_chr4_0333	Hypothetical protein	4.084	2.030009455	1.37368373236E-61	7.86817253989E-61	yes	up
PAS_chr2-2_0030	Sorting nexin family member	2.367	1.243261644	2.68470024728E-43	1.14905170584E-42	yes	up
PAS_chr2-1_0672	Scaffold protein responsible for pre-autophagosomal structure organization	2.065	1.046455263	2.35653219807E-21	6.2870028775E-21	yes	up
PAS_chr4_0779	uncharacterized protein	2.063	1.045002706	5.41931612802E-27	1.68545088484E-26	yes	up
PAS_FragB_0012	Myosin-2	2.129	1.090148965	5.24247763733E-90	4.1453490626E-89	yes	up
PAS_chr2-2_0284	uncharacterized protein	2.261	1.176695336	1.64399031097E-47	7.54345554184E-47	yes	up
PAS_chr4_0167	UDP-glucose:sterol glucosyltransferase (Sterol 3-beta-glucosyltransferase)	2.432	1.281930572	4.59733695027E-102	4.00693371281E-101	yes	up
PAS_chr4_0044	Hypothetical protein	4.14	2.049670474	1.668651984E-157	2.10317063346E-156	yes	up
PAS_chr2-1_0382	Hypothetical protein	2.62	1.389399366	4.26169710188E-15	9.32234655301E-15	yes	up
PAS_chr2-1_0203	Phosphotyrosine-specific protein phosphatase involved in the inactivation of mitogen-activated prote	13.795	3.786051669	0	0	yes	up
PAS_chr3_0842	uncharacterized protein	7.784	2.96056644	0	0	yes	up
PAS_chr3_1250	uncharacterized protein	7.79	2.961622755	7.20867749531E-235	1.22062084592E-233	yes	up
PAS_chr4_0933	uncharacterized protein	2.385	1.254015576	1.77944244248E-17	4.19935055993E-17	yes	up
PAS_chr1-1_0026	Hypothetical protein	2.966	1.56830276	3.41463839252E-99	2.9056203851E-98	yes	up
PAS_chr1-1_0128	Hypothetical protein	2.534	1.341278832	1.0281991809E-120	1.05959296736E-119	yes	up
PAS_chr3_0184	1,3-beta-glucanosyltransferase	2.612	1.385318673	1.6892824304E-46	7.64662587082E-46	yes	up
PAS_chr1-3_0120	Hypothetical protein	2.12	1.083840022	6.6721801507E-21	1.749447027E-20	yes	up
PAS_chr1-4_0473	Meiosis-specific APC/C activator protein AMA1	2.128	1.089802734	1.51501307685E-27	4.77082076612E-27	yes	up
PAS_chr1-4_0479	General amino acid permease	2.022	1.015933665	1.90031086173E-27	5.97664998352E-27	yes	up
PAS_chr1-4_0193	Protein involved in bud-site selection	2.498	1.320686616	1.32212834555E-48	6.17361508801E-48	yes	up
PAS_chr3_0418	Zinc-finger inhibitor of HO transcription	2.114	1.080288406	1.57166042162E-28	5.08942708327E-28	yes	up
PAS_chr1-1_0406	Hypothetical protein	2.453	1.294489099	2.6605711987E-55	1.39666101861E-54	yes	up
PAS_chr3_0739	Protein involved in DNA mismatch repair and crossing-over during meiotic recombination	2.566	1.359505865	2.05118545922E-56	1.08812359435E-55	yes	up
PAS_chr3_0785	uncharacterized protein	3.305	1.724583558	3.9138131667E-20	1.00370048013E-19	yes	up
PAS_chr2-1_0263	Catalytic subunit of 1,3-beta-D-glucan synthase	4.617	2.206997099	0	0	yes	up
PAS_chr3_1180	uncharacterized protein	2.962	1.566579889	5.06927382363E-58	2.77403460925E-57	yes	up
PAS_chr2-1_0335	Hypothetical protein	2.024	1.017326281	1.47397621426E-31	5.07714135721E-31	yes	up
PAS_chr2-1_0080	Peripheral protein of the cytosolic face of the mitochondrial outer membrane, required for mitochond	2.133	1.092839764	1.07525504168E-35	4.0234059558E-35	yes	up
PAS_chr2-1_0418	U2-snRNP associated splicing factor	2.412	1.27021457	1.28232823685E-89	1.01078819798E-88	yes	up
PAS_chr1-3_0043	uncharacterized protein	2.002	1.001102461	1.69750714308E-47	7.78191743165E-47	yes	up
PAS_chr2-2_0187	RNA helicase in the DEAD-box family, involved in RNA isomerization at the 5' splice site	3.635	1.862123829	9.66196625608E-117	9.69860844348E-116	yes	up
PAS_chr1-1_0123	Putative sensor/transporter protein involved in cell wall biogenesis	2.727	1.447516638	8.82270488435E-116	8.75135756675E-115	yes	up
PAS_chr4_0510	uncharacterized protein	2.229	1.156561148	1.46042405253E-61	8.35548641656E-61	yes	up
PAS_chr3_0159	Hypothetical protein	2.09	1.063411479	6.58491916459E-12	1.28404647068E-11	yes	up
PAS_chr1-3_0171	Hypothetical protein	2.419	1.274700411	3.51036481834E-15	7.70227952505E-15	yes	up
PAS_chr4_0558	Subunit of Elongator complex, which is required for modification of wobble nucleosides in tRNA	2.237	1.161425968	4.35797560318E-18	1.04712180164E-17	yes	up
PAS_chr1-1_0496	uncharacterized protein	2.827	1.499525894	6.75052218133E-31	2.29226036799E-30	yes	up
PAS_chr4_0753	Hypothetical protein	2.929	1.550334853	9.9955699244E-24	2.86262648917E-23	yes	up
PAS_chr4_0478	Phosphatidylinositol 3-kinase responsible for the synthesis of phosphatidylinositol 3-phosphate	2.237	1.161367225	3.92950900377E-69	2.50145579493E-68	yes	up
PAS_chr1-1_0148	Hypothetical protein	2.648	1.405078803	1.02656386183E-29	3.39867653796E-29	yes	up
PAS_chr1-4_0279	MAP kinase kinase kinase of the HOG1 mitogen-activated signaling pathway	2.228	1.156058984	9.73205419712E-110	9.18245789068E-109	yes	up
PAS_chr1-4_0281	Telobox-containing general regulatory factor	2.38	1.251039871	1.06802430105E-65	6.44016092326E-65	yes	up
PAS_chr1-4_0573	zinc finger protein	2.126	1.088347315	2.82878006995E-34	1.02862870367E-33	yes	up
PAS_chr2-1_0213	uncharacterized protein	2.359	1.238367078	7.240707657E-46	3.21674194408E-45	yes	up
PAS_chr1-4_0457	tRNA methyltransferase required for the synthesis of wybutosine	2.012	1.008930326	7.26088839545E-34	2.62320457907E-33	yes	up
PAS_chr2-1_0684	Histidinol-phosphate aminotransferase, catalyzes the seventh step in histidine biosynthesis	2.057	1.040766048	1.26268001353E-19	3.17977856186E-19	yes	up
PAS_chr1-1_0336	Hypothetical protein	2.319	1.213202189	1.32693096859E-47	6.09975853843E-47	yes	up
PAS_chr3_0656	uncharacterized protein	2.267	1.180638656	1.10467666089E-76	7.72659099811E-76	yes	up
PAS_chr4_0166	Putative transcription factor containing a C2H2 zinc finger	2.437	1.28528128	1.81818424254E-37	7.05528437943E-37	yes	up
PAS_chr1-4_0391	Oligopeptide transporter	2.625	1.392276023	1.56375984264E-86	1.20986896133E-85	yes	up
PAS_chr3_0463	Hypothetical protein	2.105	1.074046648	6.71506631397E-19	1.66027868697E-18	yes	up
PAS_chr4_0678	uncharacterized protein	4.509	2.172763341	0	0	yes	up
PAS_chr1-1_0317	Hypothetical protein	2.304	1.204077429	2.49322763316E-21	6.6340961731E-21	yes	up
PAS_chr3_0272	Hypothetical protein	2.401	1.263461747	2.20333857067E-20	5.69110923055E-20	yes	up
PAS_chr1-1_0187	Huge dynein-related AAA-type ATPase (midasin), forms extended pre-60S particle with the Rix1 complex	2.292	1.19651521	0	0	yes	up
PAS_chr3_1172	uncharacterized protein	2.903	1.537328116	4.03583658301E-35	1.49236927764E-34	yes	up
PAS_chr4_0426	Hypothetical protein	2.133	1.093085904	8.90645526867E-12	1.72803100101E-11	yes	up
PAS_chr4_0096	Hypothetical protein	2.627	1.393618138	6.98092662512E-42	2.92315403811E-41	yes	up

PAS_chr2-2_0114	Protein of the endoplasmic reticulum, required for GPI-phospholipase A2 activity	2.059	1.04192638	3.5269595743E-19	8.77204732896E-19	yes	up
PAS_FragB_0022	Delta(3,5)-Delta(2,4)-dienoyl-CoA isomerase, mitochondrial	13.405	3.744647489	0	0	yes	up
PAS_chr2-2_0273	Protein required for ethanol metabolism	3.36	1.748292789	3.1039864872E-25	9.22028827177E-25	yes	up
PAS_chr3_0508	uncharacterized protein	3.316	1.729515151	8.13754256341E-95	6.6978234945E-94	yes	up
PAS_chr1-4_0500	Hypothetical protein	2.108	1.075882947	6.31524536254E-17	1.4635653884E-16	yes	up
PAS_chr4_0194	Catalytic subunit of the Dcp1p-Dcp2p decapping enzyme complex	2.184	1.126984231	3.53173746975E-63	2.06284642687E-62	yes	up
PAS_chr1-3_0269	Proline oxidase, nuclear-encoded mitochondrial protein	2.169	1.116884622	5.6460356302E-30	1.88538600161E-29	yes	up
PAS_chr1-3_0299	uncharacterized protein	2.111	1.077807982	2.88587585384E-40	1.17324734592E-39	yes	up
PAS_chr4_1000	uncharacterized protein	3.372	1.753646023	1.13751218531E-39	4.59851188096E-39	yes	up
PAS_chr1-1_0177	Hypothetical protein	2.174	1.120589234	2.00161021968E-25	5.99172487783E-25	yes	up
PAS_chr3_0302	Mitochondrial cell death effector that translocates to the nucleus in response to apoptotic stimuli	2.502	1.322806668	3.05305737255E-41	1.26577292057E-40	yes	up
PAS_chr1-3_0125	Member of the protein disulfide isomerase (PDI) family	2.76	1.464732985	1.16880612018E-31	4.03426628577E-31	yes	up
PAS_chr3_0027	Hypothetical protein	2.959	1.565020176	6.75783361976E-99	5.7310531659E-98	yes	up
PAS_chr1-4_0666	uncharacterized protein	2.742	1.455228739	6.61669552957E-124	6.99062223072E-123	yes	up
PAS_chr2-1_0856	3-oxoacyl-[acyl-carrier-protein] reductase	2.368	1.24349211	1.5962497369E-16	3.65552819986E-16	yes	up
PAS_chr2-1_0156	Endosomal Na+/H+ exchanger, required for intracellular sequestration of Na+	2.008	1.005728076	8.48835991166E-33	3.00196638507E-32	yes	up
PAS_chr4_0941	uncharacterized protein	2.6	1.378692219	2.2370197082E-39	8.98560072886E-39	yes	up
PAS_chr3_1073	Peroxisomal membrane protein (PMP)	3.149	1.654899786	1.20718136348E-75	8.33916906173E-75	yes	up
PAS_chr2-1_0757	Zinc cluster transcriptional activator	2.214	1.146394698	9.20064939151E-80	6.65756342301E-79	yes	up
PAS_chr3_0773	Hypothetical protein	2.369	1.24421579	4.23175372876E-67	2.60483347637E-66	yes	up
PAS_chr3_0138	Hypothetical protein	2.048	1.034382431	2.29861356859E-19	5.74253732561E-19	yes	up
PAS_chr4_0340	Hypothetical protein	3.707	1.890217562	1.43720246792E-247	2.53603200391E-246	yes	up
PAS_chr2-1_0406	uncharacterized protein	2.168	1.116216103	2.11086350712E-12	4.20583699577E-12	yes	up
PAS_chr1-3_0095	Hypothetical protein	2.623	1.391075944	4.13456105039E-20	1.05923115244E-19	yes	up
PAS_chr1-1_0174	ATP-dependent Lon protease involved in degradation of misfolded proteins in mitochondria	3.758	1.910152408	1.11337480672E-293	2.21310747155E-292	yes	up
PAS_chr1-4_0331	Hypothetical protein	2.08	1.056244016	5.33892669893E-12	1.04839759348E-11	yes	up
PAS_chr2-1_0711	Hypothetical protein	2.028	1.019820137	3.46531964934E-12	6.84220357932E-12	yes	up
PAS_chr4_0108	Hypothetical protein	2.718	1.44267714	3.71119680101E-75	2.54967332135E-74	yes	up
PAS_chr4_0439	uncharacterized protein	3.131	1.646773192	1.14692214462E-123	1.20919737218E-122	yes	up
PAS_chr4_0546	Putative metalloprotease	2.105	1.073489396	5.27042046413E-71	3.40680520747E-70	yes	up
PAS_chr2-2_0385	Essential nucleoporin	2.126	1.088285368	2.67151232897E-87	2.08618563702E-86	yes	up
PAS_chr2-1_0547	Bisphosphate-3'-nucleotidase, involved in salt tolerance and methionine biogenesis	2.624	1.391717855	5.69467673761E-37	2.18115227064E-36	yes	up
PAS_chr1-4_0084	Chromatin modification-related protein EAF6	2.876	1.523849566	4.69346608285E-10	8.4569834936E-10	yes	up
PAS_chr3_0847	Hypothetical protein	2.595	1.375587701	1.63616996261E-54	8.49153636943E-54	yes	up
PAS_chr4_0681	GTPase-activating protein (RhoGAP) for Rho3p and Rho4p	4.143	2.050597453	9.23258272233E-204	1.38598980629E-202	yes	up
PAS_chr1-3_0197	uncharacterized protein	2.272	1.183901268	1.91929532721E-28	6.2031723654E-28	yes	up
PAS_chr1-3_0157	Subunit of the Dcp1p-Dcp2p decapping enzyme complex	2.03	1.021126334	1.7535035473E-6	2.65613534318E-6	yes	up
PAS_chr2-2_0340	Conserved zinc-finger domain protein involved in pre-mRNA splicing	2.053	1.037621343	1.95751188387E-25	5.86320861464E-25	yes	up
PAS_chr2-1_0268	Essential protein of unknown function	2.183	1.126020707	7.59006017783E-36	2.84641406669E-35	yes	up
PAS_chr2-1_0582	Hypothetical protein	2.661	1.412028504	6.15664193832E-193	8.74625771406E-192	yes	up
PAS_chr4_0241	Putative ATPase of the AAA family	2.199	1.136758755	8.02839111554E-55	4.1882550747E-54	yes	up
PAS_chr4_0496	Peroxisomal ubiquitin conjugating enzyme	6.643	2.731882049	1.64836317595E-96	1.36567024907E-95	yes	up
PAS_FragB_0027	AP-3 complex subunit delta	2.03	1.021808605	4.70981713496E-55	2.46725733039E-54	yes	up
PAS_chr4_0495	Hypothetical protein	4.455	2.155304493	1.44745425308E-168	1.90556215673E-167	yes	up
PAS_chr4_0948	uncharacterized protein	3.072	1.619235308	4.34393941398E-79	3.1163582472E-78	yes	up
PAS_chr3_1241	uncharacterized protein	2.094	1.066081546	3.34492438175E-15	7.34568764883E-15	yes	up
PAS_chr1-4_0670	uncharacterized protein	2.225	1.153644123	2.20749492711E-9	3.86676836935E-9	yes	up
PAS_chr1-1_0042	Multistep regulator of cAMP-PKA signaling	2.759	1.464375072	1.68441498821E-100	1.44802102149E-99	yes	up
PAS_chr3_1047	Small subunit of the clathrin-associated adaptor complex AP-2	2.976	1.573228648	6.40854332756E-12	1.2506233758E-11	yes	up
PAS_chr3_0524	Essential component of the nuclear pore complex	2.061	1.04315264	2.32763453702E-63	1.3642976791E-62	yes	up
PAS_chr1-3_0160	uncharacterized protein	2.512	1.328835686	4.10646626164E-69	2.61079884068E-68	yes	up
PAS_chr3_0189	Peripheral peroxisomal membrane peroxin	3.352	1.744886644	3.49052227543E-107	3.23275074091E-106	yes	up
PAS_chr2-1_0521	Hypothetical protein	2.04	1.028505864	2.30163112653E-28	7.41980957392E-28	yes	up
PAS_chr1-4_0132	Protein with similarity to mammalian monocarboxylate permease	3.544	1.825495028	7.56994867827E-113	7.34928029016E-112	yes	up
PAS_chr1-1_0150	Protein of the Sec1p/Munc-18 family, essential for vacuolar protein sorting	2.2	1.137537919	3.90626706051E-43	1.66621009731E-42	yes	up
PAS_chr4_0572	Hypothetical protein	7.778	2.959420373	0	0	yes	up
PAS_chr2-1_0334	Hypothetical protein	2.261	1.176904029	8.03421429765E-21	2.10109535636E-20	yes	up
PAS_chr2-1_0759	Mannosyltransferase, involved in asparagine-linked glycosylation in the endoplasmic reticulum (ER)	2.642	1.401606611	5.21557000662E-54	2.68191222528E-53	yes	up
PAS_chr1-1_0238	Protein whose overexpression affects chromosome stability, potential Cdc28p substrate	2.062	1.043971098	1.72465575022E-67	1.06682580171E-66	yes	up
PAS_chr1-3_0130	Pyrimidine nucleotidase	2.164	1.113578923	7.46697946413E-18	1.78222305292E-17	yes	up
PAS_FragB_0065	Putative ABC transporter	3.768	1.913686436	0	0	yes	up

PAS_chr2-2_0070	Nit protein	2.953	1.562066656	4.00760124795E-41	1.65878408856E-40	yes	up
PAS_chr2-1_0607	Hypothetical protein	2.331	1.221150942	2.25484161861E-12	4.48737574198E-12	yes	up
PAS_chr3_1173	uncharacterized protein	3.009	1.589400931	1.33047211873E-40	5.4353730992E-40	yes	up
PAS_chr1-4_0447	Hexokinase isoenzyme 2 that catalyzes phosphorylation of glucose in the cytosol	2.474	1.306880816	4.20562794007E-49	1.98034671448E-48	yes	up
PAS_chr3_0252	Steryl ester hydrolase	3.556	1.830382458	1.10374392681E-107	1.02981970463E-106	yes	up
PAS_chr2-2_0042	Specificity factor required for Rsp5p-dependent ubiquitination	2.059	1.041827243	2.77156262923E-23	7.8436626125E-23	yes	up
PAS_chr1-1_0284	Putative plasma membrane permease proposed to be involved in carboxylic acid uptake	3.889	1.959513144	3.94842519616E-142	4.65026470995E-141	yes	up
PAS_chr2-1_0701	Hypothetical protein	2.055	1.038915647	6.18851989669E-32	2.1433930138E-31	yes	up
PAS_chr4_0492	DNA N-glycosylase and apurinic/apyrimidinic (AP) lyase involved in base excision repair	2.019	1.013960928	8.77051440714E-20	2.21865779444E-19	yes	up
PAS_chr4_0343	Phosphoribosyl-5-amino-1-phosphoribosyl-4-imidazolecarboxiamide isomerase	6.584	2.718906497	1.01170192445E-151	1.22894902852E-150	yes	up
PAS_chr1-4_0309	Subunit of the SWI/SNF chromatin remodeling complex	2.112	1.078436776	5.91592276179E-58	3.23031222248E-57	yes	up
PAS_chr1-4_0492	Transcriptional modulator	2.946	1.558880167	7.1123277677E-83	5.30680954655E-82	yes	up
PAS_chr1-1_0149	Hypothetical protein	3.347	1.74299735	1.83850311707E-82	1.36369206132E-81	yes	up
PAS_chr4_0662	Aminophospholipid translocase (flippase) that maintains membrane lipid asymmetry in post-Golgi secre	2.84	1.505769301	4.14195771389E-212	6.40920164405E-211	yes	up
PAS_chr4_0604	B-type cyclin involved in cell cycle progression	2.484	1.312723238	2.42435019363E-48	1.12680749758E-47	yes	up
PAS_chr1-4_0145	pirin	4.703	2.233734072	6.38800216481E-122	6.67884883302E-121	yes	up
PAS_chr4_0577	Hypothetical protein	2.205	1.14089137	8.75735441427E-22	2.36523820351E-21	yes	up
PAS_chr1-3_0149	Transmembrane osmosensor	3.107	1.635559953	2.55615632764E-42	1.0793375459E-41	yes	up
PAS_chr4_0560	Meiosis-specific protein of unknown function, required for spore wall formation during sporulation	2.146	1.101348694	3.14290972252E-66	1.91120834275E-65	yes	up
PAS_chr4_0233	Glutamine-dependent NAD(+) synthetase	2.422	1.276039813	1.31223339047E-77	9.28160579562E-77	yes	up
PAS_chr1-4_0179	Meiosis-specific protein that initiates meiotic recombination	2.006	1.004449633	1.14608617942E-20	2.98172136383E-20	yes	up
PAS_chr1-4_0663	uncharacterized protein	5.126	2.357767268	3.48401105863E-113	3.39556039028E-112	yes	up
PAS_chr1-4_0020	Largest subunit of the Anaphase-Promoting Complex/Cyclosome (APC/C)	2.031	1.021893787	1.83787256744E-105	1.66835038658E-104	yes	up
PAS_chr3_1020	Hypothetical protein	2.188	1.129312075	3.51041322606E-24	1.01517355456E-23	yes	up
PAS_chr4_0759	C3HC4-type RING-finger peroxisomal membrane peroxin	2.269	1.182024611	9.30249528406E-35	3.41975502804E-34	yes	up
PAS_chr4_0188	Plasma membrane ATP-binding cassette (ABC) transporter	3.326	1.733986377	0	0	yes	up
PAS_chr4_0163	Abundant subunit of the nuclear pore complex (NPC)	2.107	1.075379803	5.10854159061E-113	4.96921772905E-112	yes	up
PAS_chr3_0139	Hypothetical protein	2.326	1.217682924	5.70500399302E-30	1.90381321041E-29	yes	up
PAS_chr1-4_0255	Putative zinc-cluster protein of unknown function	2.478	1.309098642	1.78082778138E-72	1.17994504777E-71	yes	up
PAS_chr1-1_0237	Nucleotide exchange factor for the endoplasmic reticulum (ER) lumenal Hsp70 chaperone Kar2p	2.606	1.381817976	4.3560287337E-45	1.91825468492E-44	yes	up
PAS_chr3_0875	uncharacterized protein	2.584	1.369596216	1.89809633387E-38	7.51024898743E-38	yes	up
PAS_chr1-3_0069	Membrane protein of unknown function	3.164	1.661723534	5.07193658908E-193	7.22571362789E-192	yes	up
PAS_chr2-1_0580	Cytosolic NADP-specific isocitrate dehydrogenase	2.684	1.424204725	6.47154070165E-59	3.58034963571E-58	yes	up
PAS_chr3_0127	Protein kinase with similarityto serine/threonine protein kinase Ypk1p	2.221	1.151234552	6.13475559603E-65	3.66409571169E-64	yes	up
PAS_chr4_0314	Phenylpyruvate decarboxylase, catalyzes decarboxylation of phenylpyruvate to phenylacetaldehyde	4.001	2.000209195	6.97027394905E-210	1.07197271222E-208	yes	up
PAS_chr2-1_0636	Signal recognition particle (SRP) receptor beta subunit	2.42	1.274819607	3.37239070217E-21	8.90275739696E-21	yes	up
PAS_chr1-3_0017	uncharacterized protein	2.201	1.138356594	6.27214916618E-46	2.79385634692E-45	yes	up
PAS_chr3_0533	Hypothetical protein	2.352	1.233734072	1.08483026185E-27	3.43120213008E-27	yes	up
PAS_chr2-1_0505	Subunit of the GINS complex (Sld5p, Psf1p, Psf2p, Psf3p)	2.009	1.006663719	2.08343044038E-8	3.47169373249E-8	yes	up
PAS_chr1-4_0548	Vacuolar proteinase B (yscB), a serine protease of the subtilisin family	3.331	1.736103722	2.8176081202E-111	2.69386905636E-110	yes	up
PAS_chr1-4_0202	Mitochondrial protein involved in protein import into the mitochondrial matrix	2.503	1.323627891	9.69316392161E-59	5.34505716686E-58	yes	up
PAS_chr4_0921	uncharacterized protein	2.291	1.195960136	9.26388353253E-76	6.40826276274E-75	yes	up
PAS_chr2-2_0481	uncharacterized protein	2.051	1.036655129	5.98322725339E-23	1.67910992507E-22	yes	up
PAS_chr4_0679	putative component of the Rpd3 histone deacetylase complex	3.027	1.597744899	2.46780870075E-65	1.48097970836E-64	yes	up
PAS_chr4_0162	Hypothetical protein	2.099	1.069476518	7.30549219026E-20	1.85271408093E-19	yes	up
PAS_chr2-2_0145	Isozyme of methylenetetrahydrofolate reductase	3.035	1.601491882	2.38648172734E-121	2.47967285264E-120	yes	up
PAS_chr3_0454	Hypothetical protein	2.039	1.028052828	4.09098627908E-17	9.55576869367E-17	yes	up
PAS_chr2-2_0296	uncharacterized protein	2.093	1.065689529	1.88158613744E-63	1.10543185574E-62	yes	up
PAS_chr1-4_0530	uncharacterized protein	2.24	1.163455008	1.88610266471E-47	8.6386250463E-47	yes	up
PAS_chr3_0990	uncharacterized protein	2.249	1.16896423	4.1081148489E-63	2.39671804816E-62	yes	up
PAS_chr1-4_0153	Alpha aminoadipate reductase	3.556	1.830303651	0	0	yes	up
PAS_chr2-1_0599	uncharacterized protein	2.083	1.058333254	2.45791176831E-152	3.0222098491E-151	yes	up
PAS_chr1-1_0248	Regulatory subunit of Nem1p-Spo7p phosphatase holoenzyme	2.571	1.362431157	8.70533047643E-36	3.26222853696E-35	yes	up
PAS_chr3_0523	uncharacterized protein	2.708	1.437149617	6.73859948224E-262	1.21900779842E-260	yes	up
PAS_chr4_0419	Putative ATPase of the AAA family	2.13	1.090826202	8.91729449345E-69	5.64088981227E-68	yes	up
PAS_chr4_0866	Mitochondrial membrane localized inositol phosphosphingolipid phospholipase C	3.053	1.610112521	6.28180467166E-79	4.48738575195E-78	yes	up
PAS_chr1-3_0238	Phosphatidylglycerolphosphate synthase, catalyzes the synthesis of phosphatidylglycerolphosphate	2.02	1.014622157	5.49346015144E-33	1.95517417563E-32	yes	up
PAS_chr3_1080	Hypothetical protein	2.816	1.493835518	2.77408743162E-60	1.56224923781E-59	yes	up
PAS_chr2-1_0385	Hypothetical protein	2.805	1.488143362	4.26279824329E-32	1.48357179E-31	yes	up
PAS_chr3_0068	Component of the RSC chromatin remodeling complex	2.023	1.016316892	1.634148099E-39	6.57976844664E-39	yes	up
PAS_chr4_0347	Peroxisomal adenine nucleotide transporter	2.51	1.327664598	4.28662021056E-50	2.05504414098E-49	yes	up

PAS_c121_0012	Hypothetical protein	2.023	1.016792444	6.55369419501E-14	1.37959514888E-13	yes	up
PAS_chr1-1_0064	uncharacterized protein	2.35	1.232430867	9.62689530432E-13	1.9419838141E-12	yes	up
PAS_chr3_0895	Serine/threonine MAP kinase	2.117	1.082128608	2.68731380557E-33	9.65321509158E-33	yes	up
PAS_chr1-1_0168	Vacuolar membrane protein that transits through the biosynthetic vacuolar protein sorting pathway	2.29	1.19546723	8.33433273024E-145	9.88522625008E-144	yes	up
PAS_chr1-3_0186	Putative protein, predicted to be an alpha-isopropylmalate carrier	2.207	1.142044397	4.86892405462E-27	1.5180297006E-26	yes	up
PAS_chr2-2_0138	One of two (see also PTH2) mitochondrially-localized peptidyl-tRNA hydrolases	2.037	1.026450255	8.61633488382E-12	1.67303274636E-11	yes	up
PAS_chr3_0052	Thiamine pyrophosphokinase, phosphorylates thiamine to produce the coenzyme thiamine pyrophosphate (	2.1	1.070549204	4.63236573388E-23	1.30218933905E-22	yes	up
PAS_chr1-1_0101	Hypothetical protein	4.088	2.031405755	8.58971210733E-188	1.20327749827E-186	yes	up
PAS_chr2-1_0775	NAD-dependent arabinose dehydrogenase, involved in biosynthesis of erythroascorbic acid	3.589	1.843423567	3.03676887467E-99	2.58845943572E-98	yes	up
PAS_chr1-3_0170	Zinc-finger DNA-binding protein	2.207	1.141832303	1.33707398848E-25	4.03126204321E-25	yes	up
PAS_chr3_0064	Hypothetical protein	2.117	1.082350333	5.59634610177E-43	2.38306727738E-42	yes	up
PAS_chr1-1_0067	Essential N-acetylglucosamine-phosphate mutase	2.031	1.022507124	1.9735992657E-39	7.94018456575E-39	yes	up
PAS_chr4_0588	Hypothetical protein	6.989	2.805016753	2.15696869699E-206	3.26728782445E-205	yes	up
PAS_chr1-1_0025	DNA repair protein	2.187	1.128879259	9.13413483622E-104	8.15906999846E-103	yes	up
PAS_chr1-3_0298	uncharacterized protein	2.147	1.102639665	2.29212035643E-22	6.32313399479E-22	yes	up
PAS_chr4_0663	Hypothetical protein	2.697	1.431433681	4.2238254521E-68	2.65852543162E-67	yes	up
PAS_FragB_0028	Hypothetical protein	2.205	1.14057934	7.38852470207E-48	3.41201935048E-47	yes	up
PAS_chr1-4_0655	tRNA wybutosine-synthesizing protein	2.369	1.24433573	5.57488328404E-26	1.69915685063E-25	yes	up
PAS_chr2-1_0443	Hypothetical protein	2.126	1.088193605	2.31687750004E-9	4.05413254965E-9	yes	up
PAS_chr2-2_0115	Boron efflux transporter of the plasma membrane	2.187	1.128942685	1.74764574705E-51	8.53292277857E-51	yes	up
PAS_chr2-1_0765	Putative FAD transporter	2.467	1.302814047	7.1422788658E-104	6.39119580357E-103	yes	up
PAS_chr2-2_0123	Hypothetical protein	2.239	1.163145339	0.000172368427427	0.000235426621817	yes	up
PAS_chr2-2_0071	Hypothetical protein	2.84	1.50571851	9.66943558832E-21	2.51956433024E-20	yes	up
PAS_chr1-1_0028	Glycerol proton symporter of the plasma membrane, subject to glucose-induced inactivation	6.088	2.605971394	0	0	yes	up
PAS_chr4_0590	Hypothetical protein	3.072	1.619019904	1.12894458995E-192	1.59928516701E-191	yes	up
PAS_chr2-1_0248	Catalytic subunit of Nem1p-Spo7p phosphatase holoenzyme	2.512	1.328822831	7.50335410742E-59	4.14663382486E-58	yes	up
PAS_chr4_0203	uncharacterized protein	2.869	1.520326844	1.29785400103E-214	2.04605259285E-213	yes	up
PAS_chr1-1_0197	uncharacterized protein	2.146	1.101537118	5.97771194052E-46	2.66506324015E-45	yes	up
PAS_chr1-1_0258	Essential protein that associates with the contractile actomyosin ring	2.599	1.378011445	8.00751925474E-74	5.40534420565E-73	yes	up
PAS_chr1-1_0129	uncharacterized protein	3.472	1.795771499	4.44086430526E-115	4.38764373107E-114	yes	up
PAS_chr1-4_0232	Plasma membrane protein of unknown function	3.105	1.634461454	1.00669766375E-251	1.78893376361E-250	yes	up
PAS_chr2-2_0022	Ubiquitin-binding component of the Rsp5p E3-ubiquitin ligase complex, functional homolog of Bul2p	2.099	1.069511294	1.89740110494E-67	1.172239577E-66	yes	up
PAS_chr3_0029	Hypothetical protein	2.704	1.434977337	9.76020893386E-95	8.02027626281E-94	yes	up
PAS_chr4_0671	Putative transporter, member of the mitochondrial carrier family	3.382	1.757750421	3.38506026884E-79	2.43192401314E-78	yes	up
PAS_chr4_0014	Hypothetical protein	2.311	1.208804622	2.73718247606E-101	2.37332597795E-100	yes	up
PAS_chr3_0245	Component of the Sin3p-Rpd3p histone deacetylase complex	2.417	1.273380901	1.93493900239E-214	3.02199013758E-213	yes	up
PAS_chr2-1_0488	Hypothetical protein	9.565	3.25771175	0	0	yes	up
PAS_chr4_0399	Ferric reductase and cupric reductase	2.445	1.289844443	1.39346408983E-103	1.24030635536E-102	yes	up
PAS_chr4_0269	Essential nuclear protein with a possible role in the osmoregulatory glycerol response	2.083	1.058721519	1.13397581309E-88	8.89666827464E-88	yes	up
PAS_chr2-1_0071	Putative protein kinase, possible substrate of cAMP-dependent protein kinase (PKA)	2.492	1.317102279	1.24632458946E-59	6.95645544996E-59	yes	up
PAS_chr4_0865	L-homoserine-O-acetyltransferase, catalyzes the conversion of homoserine to O-acetyl homoserine	2.384	1.253513449	4.75066469906E-61	2.69955850526E-60	yes	up
PAS_chr4_0947	uncharacterized protein	12.135	3.601108088	0	0	yes	up
PAS_chr3_0264	Ferrochelatase	2.36	1.238809609	8.59763323393E-45	3.77290554393E-44	yes	up
PAS_chr4_0792	Putative mitochondrial transport protein	2.519	1.332983975	8.97183847778E-40	3.63279997623E-39	yes	up
PAS_chr4_0483	Protein required for normal intracellular sterol distribution and for sphingolipid metabolism	2.006	1.004405084	8.13553946043E-21	2.12648793901E-20	yes	up
PAS_chr4_0615	Putative S-adenosylmethionine-dependent methyltransferase of the seven beta-strand family	3.074	1.620246898	1.0670282323E-45	4.7278281764E-45	yes	up
PAS_chr1-1_0403	Hypothetical protein	3.272	1.710106581	2.22190603261E-209	3.39634207841E-208	yes	up
PAS_chr3_0073	Glycerol 3-phosphate/dihydroxyacetone phosphate dual substrate-specific sn-1 acyltransferase of the	2.489	1.315392316	7.24525327971E-119	7.37578517078E-118	yes	up
PAS_chr3_0438	uncharacterized protein	2.055	1.039400505	6.14989542372E-53	3.08353181315E-52	yes	up
PAS_chr1-4_0149	3-ketosphinganine reductase, catalyzes the second step in phytosphingosine synthesis	2.094	1.06638019	1.27429071464E-29	4.19948099862E-29	yes	up
PAS_chr2-1_0221	Nicotinate phosphoribosyltransferase, acts in the salvage pathway of NAD+ biosynthesis	2.395	1.260307909	5.60857131724E-54	2.87811277086E-53	yes	up
PAS_chr1-1_0371	multi-metabolic pathway regulator RTG1	2.788	1.4794457	2.48794228548E-39	9.96961095909E-39	yes	up
PAS_chr1-1_0140	Hypothetical protein	2.524	1.335852325	1.04212508769E-77	7.38147474085E-77	yes	up
PAS_chr2-1_0299	Protein involved in proteasome-dependent catabolite degradation of fructose-1,6-bisphosphatase	2.31	1.2076393	5.44588834019E-73	3.65164966171E-72	yes	up
PAS_chr1-3_0165	uncharacterized protein	6.345	2.665726771	3.24952861117E-303	6.56300376932E-302	yes	up
PAS_chr4_0254	uncharacterized protein	2.327	1.218506221	4.23158575826E-85	3.21946214498E-84	yes	up
PAS_chr1-3_0249	Hypothetical protein	7.824	2.967981683	0	0	yes	up
PAS_chr4_0631	Hypothetical protein	2.53	1.339018439	3.11161772251E-60	1.75037198283E-59	yes	up
PAS_chr3_0594	F-box protein containing five copies of the WD40 motif, controls cell cycle function	2.142	1.099000187	6.95264560649E-65	4.14766960321E-64	yes	up
PAS_chr3_0688	E2-like enzyme involved in autophagy and the cytoplasm-to-vacuole targeting (Cvt) pathway	3.551	1.828053305	4.04415184839E-97	3.36165944555E-96	yes	up
PAS_chr1-4_0304	Acetyl-CoA C-acetyltransferase (acetoacetyl-CoA thiolase), cytosolic enzyme	2.128	1.089392354	3.62000699314E-38	1.42004798506E-37	yes	up

PAS_chr4_0664	S-adenosylmethionine-homocysteine methyltransferase	2.829	1.500111589	1.65589771179E-60	9.34625094565E-60	yes	up
PAS_chr2-1_0466	Triacylglycerol lipase involved in triacylglycerol mobilization and degradation	2.076	1.053970426	2.6503335486E-66	1.61950515382E-65	yes	up
PAS_chr1-1_0265	Hypothetical protein	2.142	1.099063427	1.57658626607E-36	5.9974677247E-36	yes	up
PAS_chr3_0271	Lysophospholipid acyltransferase	2.164	1.113694288	1.32382915409E-60	7.48037844486E-60	yes	up
PAS_chr2-1_0528	Hypothetical protein	2.115	1.080859601	1.66709812097E-11	3.18898305454E-11	yes	up
PAS_chr4_0829	uncharacterized protein	3.615	1.853871593	3.38046667315E-67	2.08337829648E-66	yes	up
PAS_chr3_0570	Cardiolipin synthase	2.378	1.249890838	1.21164229593E-37	4.71256698083E-37	yes	up
PAS_chr3_0743	uncharacterized protein	2.017	1.012254363	5.1415862082E-22	1.40374793925E-21	yes	up
PAS_chr4_0802	uncharacterized protein	2.112	1.078508034	2.29936816926E-63	1.34930251146E-62	yes	up
PAS_FragB_0030	Hypothetical protein	5.002	2.322434278	4.87048540807E-106	4.45339474858E-105	yes	up
PAS_chr3_1157	uncharacterized protein	2.864	1.51813268	1.84528906385E-130	2.05308820843E-129	yes	up
PAS_chr3_0111	uncharacterized protein	3.984	1.994312616	1.07428506527E-271	2.02343804991E-270	yes	up
PAS_chr2-2_0372	Mitochondrial intermembrane space protein that functions in mitochondrial copper homeostasis	3.366	1.750831008	4.03201275223E-7	6.32272907108E-7	yes	up
PAS_chr2-1_0453	Cytoplasmic aldehyde dehydrogenase, involved in ethanol oxidation and beta-alanine biosynthesis	2.78	1.475238078	5.29833023682E-105	4.79232064046E-104	yes	up
PAS_chr1-3_0049	Hypothetical protein	2.355	1.235797718	6.0471461088E-12	1.18239104903E-11	yes	up
PAS_chr2-1_0708	Mitochondrial inner membrane protein	2.322	1.215633937	1.07170112737E-32	3.78216489092E-32	yes	up
PAS_FragB_0044	Transcriptional coactivator HFI1/ADA1	2.129	1.089906409	1.14982693225E-45	5.08573407414E-45	yes	up
PAS_chr4_0321	uncharacterized protein	2.158	1.109453384	4.59615658578E-92	3.69234368529E-91	yes	up
PAS_c131_0004	Hypothetical protein	2.931	1.551478217	3.65647361358E-239	6.29739924749E-238	yes	up
PAS_chr1-3_0036	uncharacterized protein	2.341	1.22688329	2.09806588654E-75	1.44734888113E-74	yes	up
PAS_chr1-4_0565	Hypothetical protein	2.387	1.255337905	1.63104770371E-37	6.33400687409E-37	yes	up
PAS_chr1-4_0354	Catalytic subunit of an adoMet-dependent tRNA methyltransferase complex (Trm11p-Trm112p)	2.069	1.048663427	7.9080709271E-42	3.30313029006E-41	yes	up
PAS_chr3_0543	Zinc transporter	2.411	1.269504566	5.26861398527E-65	3.15426901571E-64	yes	up
PAS_chr1-3_0201	Hypothetical protein	2.304	1.204280468	2.03148171857E-85	1.55263245634E-84	yes	up
PAS_chr2-1_0591	Coronin, cortical actin cytoskeletal component	2.207	1.142250115	1.70235289991E-79	1.22652331428E-78	yes	up
PAS_chr2-2_0128	ATPase component of the RSC chromatin remodeling complex	2.347	1.230811315	4.65221837613E-198	6.72299029125E-197	yes	up
PAS_chr3_0039	Beta-isopropylmalate dehydrogenase (IMDH), catalyzes the third step in the leucine biosynthesis path	2.058	1.041193787	1.52469067606E-34	5.57243416419E-34	yes	up
PAS_chr4_0978	uncharacterized protein	5.889	2.557926802	9.34656234387E-291	1.85054574911E-289	yes	up
PAS_chr1-4_0433	uncharacterized protein	2.468	1.303443044	2.60650967207E-58	1.43101933852E-57	yes	up
PAS_chr4_0676	Hypothetical protein	2.021	1.015232161	2.9150298015E-45	1.28706627496E-44	yes	up
PAS_chr2-2_0205	Essential component of the Sorting and Assembly Machinery of the mitochondrial outer membrane	2.228	1.155579935	1.46368769041E-59	8.16062682382E-59	yes	up
PAS_chr4_0213	Ribose-5-phosphate ketol-isomerase	4.402	2.138265192	7.74967784013E-117	7.7946259716E-116	yes	up
PAS_chr1-1_0027	uncharacterized protein	2.493	1.318153349	1.45778198395E-34	5.33953794414E-34	yes	up
PAS_chr2-1_0381	Hypothetical protein	2.179	1.123454268	1.36485942659E-8	2.29561139007E-8	yes	up
PAS_chr3_0110	Nucleolar protein involved in pre-rRNA processing	2.376	1.248242291	4.54182781319E-106	4.16044664345E-105	yes	up
PAS_chr1-3_0127	Hypothetical protein	2.052	1.036786422	1.49313754981E-39	6.02164293345E-39	yes	up
PAS_chr3_0768	Protein involved in the regulation of cell wall synthesis	2.157	1.108921156	2.67704765905E-51	1.3032790588E-50	yes	up
PAS_chr2-1_0110	Fructose-2,6-bisphosphatase, required for glucose metabolism	2.865	1.518639979	5.95186484227E-110	5.63689798338E-109	yes	up
PAS_chr1-1_0159	uncharacterized protein	3.792	1.92311143	1.3147055629E-123	1.38319127109E-122	yes	up
PAS_chr2-2_0230	One of two isozymes of HMG-CoA reductase that catalyzes the conversion of HMG-CoA to mevalonate	2.096	1.067829995	4.90926263527E-119	5.00784620543E-118	yes	up
PAS_chr4_0418	uncharacterized protein	2.339	1.226049742	2.42571233277E-47	1.10899157468E-46	yes	up
PAS_chr4_0006	High affinity nicotinic acid plasma membrane permease	2.505	1.32482239	1.19297765523E-62	6.90389485403E-62	yes	up
PAS_chr1-4_0195	Putative sulfate permease	2.313	1.210075891	3.19750976405E-104	2.86635946585E-103	yes	up
PAS_chr1-1_0220	Transporter of the ATP-binding cassette (ABC) family involved in bile acid transport	2.32	1.213984723	9.03485594186E-283	1.76109653223E-281	yes	up
PAS_chr2-2_0045	Nucleolar protein, component of the small subunit (SSU) processome containing the U3 snoRNA	2.024	1.017454615	1.12871323962E-205	1.70459425888E-204	yes	up
PAS_chr2-1_0776	Multifunctional enzyme of the peroxisomal fatty acid beta-oxidation pathway	5.472	2.452020969	0	0	yes	up
PAS_chr4_0962	uncharacterized protein	4.666	2.222174251	0	0	yes	up
PAS_chr2-1_0279	Triacylglycerol lipase of the lipid particle, responsible for all the TAG lipase activity of the lip	2.464	1.300831772	1.50873981972E-101	1.31044085551E-100	yes	up
PAS_chr2-2_0267	3-ketoacyl-CoA thiolase with broad chain length specificity	8.417	3.073285281	0	0	yes	up
PAS_chr3_0504	Hypothetical protein	2.082	1.057796113	1.19829556889E-69	7.65721526802E-69	yes	up
PAS_chr4_0991	uncharacterized protein	2.609	1.383323668	3.57937455918E-52	1.76477202531E-51	yes	up
PAS_chr2-2_0152	Hypothetical protein	2.158	1.109449954	1.36815455451E-98	1.15832479034E-97	yes	up
PAS_chr1-1_0051	uncharacterized protein	2.502	1.323174941	2.0504469262E-119	2.09587349428E-118	yes	up
PAS_chr4_0206	Hypothetical protein	2.079	1.055736462	1.75979411822E-19	4.41616997032E-19	yes	up
PAS_chr2-1_0049	Aminophospholipid translocase (flippase) that maintains membrane lipid asymmetry in post-Golgi secre	2.387	1.255225839	2.12276953638E-102	1.85982717743E-101	yes	up
PAS_chr2-1_0875	uncharacterized protein	2.472	1.305419859	7.55975733513E-7	1.17050553074E-6	yes	up
PAS_chr4_0463	Hypothetical protein	4.17	2.059950689	2.64223298435E-263	4.79703598495E-262	yes	up
PAS_chr2-2_0322	Hypothetical protein	2.009	1.006138051	2.40821932428E-16	5.48502490117E-16	yes	up
PAS_chr3_0687	Hypothetical protein	2.003	1.002063666	2.99800789985E-34	1.08937729251E-33	yes	up
PAS_chr1-1_0070	Steryl ester hydrolase, one of three gene products (Yeh1p, Yeh2p, Tgl1p)	2.473	1.306290395	4.2847736693E-81	3.1411263532E-80	yes	up
PAS_chr2-2_0330	Phosphatidylinositol:ceramide phosphoinositol transferase (IPC synthase)	2.063	1.044562211	5.59560094179E-40	2.27304338742E-39	yes	up

PAS_chr2-2_0226	Hypothetical protein	2.048	1.033998514	5.63473588348E-112	5.41818102448E-111	yes	up
PAS_chr4_0266	uncharacterized protein	3.891	1.960080974	9.17109784743E-268	1.6894304423E-266	yes	up
PAS_chr3_0845	Lyso-phosphatidylcholine acyltransferase	2.054	1.038286885	1.33687639214E-42	5.65921833001E-42	yes	up
PAS_chr2-1_0309	uncharacterized protein	4.005	2.001975234	1.03581652677E-283	2.02689545257E-282	yes	up
PAS_chr1-1_0016	transcription factor	2.023	1.016148658	2.82788228386E-84	2.13214692736E-83	yes	up
PAS_chr2-1_0157	Hypothetical protein	2.054	1.0385047	3.50592191499E-16	7.94918003177E-16	yes	up
PAS_chr1-3_0250	Mitochondrial carrier protein involved in the accumulation of CoA in the mitochondrial matrix	10.972	3.455764932	0	0	yes	up
PAS_chr3_0666	Hypothetical protein	2.218	1.149121256	1.32536795019E-42	5.6152278193E-42	yes	up
PAS_chr1-4_0579	uncharacterized protein	2.679	1.421629111	2.99558153272E-101	2.59290525439E-100	yes	up
PAS_chr1-4_0516	Putative transcription factor	4.608	2.204049406	9.60634710002E-217	1.5288075812E-215	yes	up
PAS_chr3_0831	Alpha subunit of succinyl-CoA ligase	6.494	2.699198239	0	0	yes	up
PAS_chr3_1016	Phosphoinositide binding protein	2.378	1.249581269	6.51568573369E-68	4.07048242916E-67	yes	up
PAS_chr4_0542	Member of the ChAPs family of proteins (Chs5p-Arf1p-binding proteins: Bch1p, Bch2p, Bud7p, Chs6p)	2.218	1.148939587	7.6292633812E-108	7.14479805289E-107	yes	up
PAS_chr3_0836	Hypothetical protein	9.24	3.207921308	0	0	yes	up
PAS_chr2-1_0183	Acyl-protein thioesterase responsible for depalmitoylation of Gpa1p	2.189	1.130288144	1.57001943845E-23	4.47851829607E-23	yes	up
PAS_chr3_1002	Putative nicotinamide N-methyltransferase	2.33	1.220372999	2.38838976402E-33	8.58556978073E-33	yes	up
PAS_FragB_0005	Ino eighty subunit 1	2.134	1.093435221	5.39321504209E-107	4.97660154984E-106	yes	up
PAS_chr3_1010	uncharacterized protein	3.582	1.840634629	1.70035338364E-217	2.71462767185E-216	yes	up
PAS_chr3_0390	Subunit of RAVE (Rav1p, Rav2p, Skp1p)	2.614	1.386330232	2.41024789018E-77	1.69526386569E-76	yes	up
PAS_chr1-1_0147	Putative mannosidase, essential glycosylphosphatidylinositol (GPI)-anchored membrane protein	2.24	1.163657166	2.87764304345E-66	1.75414143824E-65	yes	up
PAS_chr4_0583	Hypothetical protein	2.25	1.169669643	1.83888799926E-141	2.15064366239E-140	yes	up
PAS_chr3_1074	Hypothetical protein	3.06	1.613468365	4.23301742863E-13	8.6535953856E-13	yes	up
PAS_chr2-1_0707	putative serine protease of the SPS plasma membrane amino acid sensor system (Ssy1p-Ptr3p-Ssy5p), wh	2.328	1.219099673	4.08691977346E-133	4.62908097765E-132	yes	up
PAS_chr1-1_0288	Subunit of the RNA polymerase II mediator complex	2.742	1.454987708	9.46292963132E-17	2.18398683414E-16	yes	up
PAS_chr3_1235	uncharacterized protein	2.014	1.010393221	4.54781190892E-58	2.49138846296E-57	yes	up
PAS_chr1-3_0111	Hypothetical protein	2.16	1.110968025	5.19068853341E-8	8.49462174894E-8	yes	up
PAS_chr2-2_0271	GPI-anchored cell wall protein of unknown function	2.018	1.013165945	1.12086488277E-43	4.81780298757E-43	yes	up
PAS_chr3_0121	Hypothetical protein	3.357	1.747219754	6.74568166298E-9	1.15466416212E-8	yes	up
PAS_chr2-2_0495	uncharacterized protein	2.587	1.371337378	1.88378254361E-129	2.09128971564E-128	yes	up
PAS_chr4_0756	Mitochondrial protein kinase	3.451	1.786953865	5.14716386011E-237	8.77460578051E-236	yes	up
PAS_chr3_0069	uncharacterized protein	10.443	3.384403032	0	0	yes	up
PAS_chr4_0285	uncharacterized protein	3.937	1.97711769	2.14009241242E-165	2.79546097197E-164	yes	up
PAS_chr2-2_0407	Beta subunit of succinyl-CoA ligase, which is a mitochondrial enzyme of the TCA cycle	4.88	2.286751027	0	0	yes	up
PAS_chr4_0827	Hypothetical protein	2.827	1.499376983	1.12601526646E-144	1.33240724119E-143	yes	up
PAS_chr3_0854	Hypothetical protein	3.595	1.846115793	5.09228336927E-129	5.64076939737E-128	yes	up
PAS_chr2-1_0715	PEX19; peroxisomal biogenesis protein	2.803	1.486859482	1.84926866289E-76	1.29166279245E-75	yes	up
PAS_chr4_0147	Aromatic aminotransferase II	12.473	3.640784196	0	0	yes	up
PAS_chr2-2_0049	NAD(+)-dependent histone deacetylase	2.042	1.029785417	1.73755495342E-36	6.60481017441E-36	yes	up
PAS_chr3_1063	Hypothetical protein	2.672	1.418035943	4.56259665996E-126	4.93447281784E-125	yes	up
PAS_chr2-1_0329	uncharacterized protein	2.07	1.049519042	1.28873043488E-124	1.37894156532E-123	yes	up
PAS_chr3_1000	Hypothetical protein	2.495	1.318774495	1.7870066341E-23	5.08306355366E-23	yes	up
PAS_chr3_0761	Carnitine acetyltransferase	7.956	2.992007608	0	0	yes	up
PAS_chr3_0366	Hypothetical protein	2.064	1.045484458	4.75534189043E-48	2.20208235423E-47	yes	up
PAS_chr3_0464	uncharacterized protein	2.53	1.339371179	4.38277446457E-82	3.24609319327E-81	yes	up
PAS_chr2-2_0201	Putative mannosyltransferase involved in protein glycosylation	2.247	1.167941204	2.4158269443E-94	1.97547865087E-93	yes	up
PAS_chr2-1_0480	Mitochondrial inner membrane protein	2.791	1.481020848	3.89404026269E-158	4.92038404046E-157	yes	up
PAS_chr3_0260	Hypothetical protein	2.153	1.106229641	4.05422677038E-54	2.08900680617E-53	yes	up
PAS_chr3_0665	17-kDa component of the U4/U6aU5 tri-snRNP, plays an essential role in pre-mRNA splicing	2.146	1.101852014	1.08611683334E-11	2.09435642441E-11	yes	up
PAS_chr4_0459	Conserved protein involved in autophagy and the Cvt pathway	2.015	1.010674654	7.62961675443E-34	2.75443953037E-33	yes	up
PAS_chr2-1_0127	Hypothetical protein	2.578	1.366094528	6.87190782351E-52	3.37488519965E-51	yes	up
PAS_chr1-1_0305	Rho GDP dissociation inhibitor involved in the localization and regulation of Cdc42p	2.319	1.213461677	5.08771815522E-27	1.58428078035E-26	yes	up
PAS_chr3_0746	uncharacterized protein	2.342	1.227959332	2.59741366015E-38	1.02209650211E-37	yes	up
PAS_chr1-3_0081	Glycerol-3-phosphate/dihydroxyacetone phosphate dual substrate-specific sn-1 acyltransferase	3.233	1.692892464	0	0	yes	up
PAS_chr2-1_0177	Hypothetical protein	2.715	1.441083346	1.043361021E-102	9.18925144418E-102	yes	up
PAS_chr4_0666	putative protein kinase, overexpression causes sensitivity to staurosporine	3.982	1.993513411	5.62246403353E-259	1.00983470088E-257	yes	up
PAS_chr1-1_0352	Hypothetical protein	2.343	1.228626063	4.39218150472E-35	1.6229449513E-34	yes	up
PAS_chr4_0986	uncharacterized protein	2.395	1.260286982	4.25968824081E-84	3.20688206033E-83	yes	up
PAS_chr4_0011	Maltose permease, high-affinity maltose transporter (alpha-glucoside transporter)	2.307	1.206153977	1.04288702543E-113	1.02235455183E-112	yes	up
PAS_chr3_0466	Protein involved in bud-site selection	2.019	1.013984995	1.23065908476E-30	4.16486173437E-30	yes	up
PAS_chr4_0182	uncharacterized protein	2.19	1.13091448	5.55823786711E-53	2.78965850636E-52	yes	up
PAS_chr1-1_0193	Hypothetical protein	2.009	1.006175993	2.85989760241E-16	6.5020004713E-16	yes	up

PAS_chr4_0375	Hypothetical protein	4.616	2.206778825	5.23749441038E-136	6.04113747473E-135	yes	up
PAS_chr3_0714	Putative divalent metal ion transporter involved in iron homeostasis	2.397	1.261368042	2.48012387715E-111	2.37572247204E-110	yes	up
PAS_chr1-1_0201	G protein beta subunit, forms a dimer with Ste18p to activate the mating signaling pathway	3.011	1.590108171	7.73312966605E-140	9.02318076347E-139	yes	up
PAS_chr2-2_0251	Hypothetical protein	2.067	1.047338701	1.44165447557E-7	2.30453921095E-7	yes	up
PAS_chr3_0028	Putative protein with similarity to the allantoate permease (Dal5p) subfamily of the major facilitat	2.359	1.237895016	1.91061996656E-122	2.00177246079E-121	yes	up
PAS_chr2-1_0641	Protein serine/threonine kinase required for vesicle formation in autophagy	2.397	1.261250225	1.09244985111E-201	1.63024638018E-200	yes	up
PAS_chr4_0622	Hypothetical protein	6.134	2.616803377	0	0	yes	up
PAS_chr1-4_0637	Protein kti12	2.05	1.035814702	2.26279159262E-37	8.74679394259E-37	yes	up
PAS_chr4_0043	Mitochondrial aldehyde dehydrogenase	16.144	4.012961764	0	0	yes	up
PAS_chr3_0915	Quinolinate phosphoribosyl transferase	2.293	1.197016076	1.14037913203E-54	5.9368184834E-54	yes	up
PAS_chr1-4_0478	Regulatory, non-ATPase subunit of the 26S proteasome	6.583	2.718650602	3.09493799017E-305	6.27598514217E-304	yes	up
PAS_chr4_0116	Protein involved in N-glycosylation	3.352	1.745066299	1.27809690995E-127	1.39729333916E-126	yes	up
PAS_chr1-1_0300	Plasma membrane glucose sensor that regulates glucose transport	3.03	1.599548505	0	0	yes	up
PAS_chr4_0554	Hypothetical protein	2.254	1.172652425	9.29600452554E-15	2.01333362442E-14	yes	up
PAS_chr3_1252	uncharacterized protein	2.051	1.036494632	1.45569412392E-24	4.24141700417E-24	yes	up
PAS_chr3_0681	RNA exonuclease, required for U4 snRNA maturation	2.369	1.244514935	9.09432895233E-37	3.47533284964E-36	yes	up
PAS_chr1-4_0253	ATP sulfurylase, catalyzes the primary step of intracellular sulfate activation	2.758	1.463505761	6.68526638337E-199	9.71682215086E-198	yes	up
PAS_chr3_0394	Putative GPI-anchored aspartic protease	2.272	1.183737868	2.61526786827E-128	2.88425046262E-127	yes	up
PAS_chr1-4_0420	Mitochondrial protein, putative inner membrane transporter	2.028	1.020042079	2.31466777581E-38	9.13694210718E-38	yes	up
PAS_chr3_0065	RNA binding protein with preference for single stranded tracts of U's involved in synthesis of both	2.061	1.04359511	0	0	yes	up
PAS_chr1-3_0020	Hypothetical protein	2.551	1.351304178	3.32554107054E-107	3.08563580143E-106	yes	up
PAS_chr3_0167	3,4-dihydroxy-2-butanone-4-phosphate synthase (DHBP synthase), required for riboflavin biosynthesis	2.537	1.343037927	2.71056680977E-42	1.14261864932E-41	yes	up
PAS_chr2-2_0207	uncharacterized protein	4.062	2.022259955	4.53887016478E-297	9.13039122346E-296	yes	up
PAS_chr1-1_0131	General transcriptional co-repressor, acts together with Tup1p	2.386	1.254333064	5.32777126331E-197	7.67718099804E-196	yes	up
PAS_chr2-2_0319	Hypothetical protein	2.738	1.452963639	2.19844413143E-199	3.23274138508E-198	yes	up
PAS_chr3_1001	General repressor of transcription, forms complex with Cyc8p	2.139	1.096634971	5.54619459989E-114	5.45828036064E-113	yes	up
PAS_chr4_0794	Peroxisomal membrane peroxin that is a central component of the peroxisomal protein import machinery	3.281	1.714110302	1.72058630237E-226	2.79123500472E-225	yes	up
PAS_chr3_0392	Putative nucleotide sugar transporter	3.508	1.810805138	4.03913693973E-199	5.90488943892E-198	yes	up
PAS_chr2-2_0202	uncharacterized protein	2.38	1.251128573	1.98244497649E-133	2.25559180696E-132	yes	up
PAS_chr1-4_0222	Bifunctional dehydrogenase and ferrochelatase, involved in the biosynthesis of siroheme	2.28	1.188802165	3.11941281028E-52	1.54101444233E-51	yes	up
PAS_chr2-1_0131	Hypothetical protein	2.249	1.169488822	3.96938135315E-23	1.11769422312E-22	yes	up
PAS_chr3_0122	Small subunit of the clathrin-associated adaptor complex AP-3, which is involved in vacuolar protein	2.415	1.272061262	8.30970454588E-25	2.43955073913E-24	yes	up
PAS_chr3_0026	Hypothetical protein	4.191	2.067145496	1.8614556957E-237	3.18410227677E-236	yes	up
PAS_chr2-1_0468	Catalytic (alpha) subunit of C-terminal domain kinase I (CTDK-I), which phosphorylates the C-termina	2.049	1.03475278	3.57831422984E-116	3.57760283536E-115	yes	up
PAS_chr3_1099	Glycerol proton symporter of the plasma membrane, subject to glucose-induced inactivation	4.191	2.06745056	0	0	yes	up
PAS_chr4_0193	Hypothetical protein	2.969	1.570199882	4.79724110927E-121	4.95386561365E-120	yes	up
PAS_chr4_0783	Glycerol kinase, converts glycerol to glycerol-3-phosphate	6.712	2.746639234	0	0	yes	up
PAS_chr4_0472	Conserved protein involved in exocytic transport from the Golgi	3.181	1.66954039	1.16535340939E-243	2.02787622694E-242	yes	up
PAS_chr1-1_0326	Hypothetical protein	2.033	1.023937084	1.97785483655E-57	1.06609131544E-56	yes	up
PAS_chr1-4_0635	Hypothetical protein	2.059	1.04211489	7.64852651112E-26	2.32273187346E-25	yes	up
PAS_chr1-4_0361	Hypothetical protein	2.734	1.451038539	1.70690216238E-132	1.9246661378E-131	yes	up
PAS_chr4_0149	Microsomal cytochrome b reductase	2.217	1.148711707	1.45572496992E-52	7.23403248391E-52	yes	up
PAS_chr1-4_0249	Acetyl-CoA carboxylase, biotin containing enzyme	2.688	1.426391095	0	0	yes	up
PAS_chr2-2_0063	NADPH-dependent 1-acyl dihydroxyacetone phosphate reductase	3.007	1.588216803	4.2393851913E-124	4.50737169705E-123	yes	up
PAS_chr2-2_0084	Repressible alkaline phosphatase, a glycoprotein localized to the vacuole	2.202	1.138521552	1.23617921487E-118	1.25590813567E-117	yes	up
PAS_chr1-1_0122	Hypothetical protein	2.516	1.331247261	5.62571370216E-74	3.80776772653E-73	yes	up
PAS_chr2-1_0494	uncharacterized protein	2.03	1.021737085	1.06428644643E-18	2.60579188856E-18	yes	up
PAS_chr4_0703	Imidazole glycerol phosphate synthase (glutamine amidotransferase:cyclase)	2.531	1.339647705	8.69912308942E-194	1.24283778457E-192	yes	up
PAS_chr3_0336	Hypothetical protein	2.467	1.302883793	4.80664406957E-61	2.72521003674E-60	yes	up
PAS_chr3_0569	Biotin:apoprotein ligase, covalently modifies proteins with the addition of biotin	3.44	1.782573777	0	0	yes	up
PAS_chr4_0812	Protein serine/threonine/tyrosine (dual-specificity) kinase	2.061	1.043482965	7.62466072613E-67	4.68758175938E-66	yes	up
PAS_chr3_0910	uncharacterized protein	2.138	1.096088392	5.52520776561E-46	2.46769714505E-45	yes	up
PAS_chr1-4_0213	Hypothetical protein	2.74	1.454367705	0	0	yes	up
PAS_chr1-3_0013	Hypothetical protein	3.314	1.72863469	5.31304464522E-89	4.17489086263E-88	yes	up
PAS_chr2-1_0289	Hypothetical protein	2.987	1.578788726	6.08111526277E-68	3.80845935946E-67	yes	up
PAS_chr2-1_0786	uncharacterized protein	2.458	1.297321252	1.74042968974E-120	1.78990202653E-119	yes	up
PAS_chr4_0369	Subunit alpha of assimilatory sulfite reductase	3.532	1.820484466	0	0	yes	up
PAS_chr3_0307	Constituent of the mitochondrial inner membrane presequence translocase (TIM23 complex)	2.239	1.162998017	9.63747949839E-50	4.58099096384E-49	yes	up
PAS_chr4_0240	uncharacterized protein	4.645	2.215720009	0	0	yes	up
PAS_chr1-1_0205	Hypothetical protein	2.131	1.09132464	3.88936995537E-9	6.73077821939E-9	yes	up
PAS_chr3_0094	Constituent of 66S pre-ribosomal particles, required for large (60S) ribosomal subunit biogenesis	2.517	1.331549705	0	0	yes	up

PAS_chr3_1203	uncharacterized protein	2.865	1.51871103	2.18452113493E-37	8.45073599044E-37	yes	up
PAS_chr2-1_0762	General transcriptional co-repressor, acts together with Tup1p	2.022	1.015863177	2.95708785937E-169	3.91347232757E-168	yes	up
PAS_chr3_0092	Transcription factor required for full Ty1 epxression, Ty1-mediated gene activation, and haploid inv	4.495	2.168388281	0	0	yes	up
PAS_chr1-4_0075	L-rhamnonate dehydratase	2.613	1.385739464	3.01412498587E-165	3.92695195698E-164	yes	up
PAS_chr3_1232	uncharacterized protein	3.197	1.6765495	2.60683208468E-86	2.0045502376E-85	yes	up
PAS_chr3_0413	Protein with a potential role in pre-rRNA processing	2.187	1.129155158	2.37322588886E-38	9.36074744712E-38	yes	up
PAS_chr4_0396	Heme-binding protein involved in regulation of cytochrome P450 protein Erg11p	2.086	1.060745408	3.62015733208E-19	8.99494625643E-19	yes	up
PAS_chr1-4_0368	Protein kinase required for signal transduction during entry into meiosis	4.582	2.195858657	0	0	yes	up
PAS_chr1-1_0071	Hypothetical protein	2.079	1.055991925	6.60438779713E-81	4.81354583069E-80	yes	up
PAS_chr1-4_0282	uncharacterized protein	2.377	1.249363477	1.11570755985E-44	4.88328400217E-44	yes	up
PAS_chr4_0462	Hypothetical protein	2.191	1.131575588	7.29789412861E-91	5.82557294806E-90	yes	up
PAS_chr2-2_0142	Bifunctional carbamoylphosphate synthetase (CPSase)-aspartate transcarbamylase (ATCase)	2.349	1.231943666	0	0	yes	up
PAS_chr2-1_0121	Phosphoribosyl-glycinamide transformylase, catalyzes a step in the 'de novo' purine nucleotide biosy	2.061	1.043039083	2.75707065615E-33	9.89672257659E-33	yes	up
PAS_chr4_0448	Lsm (Like Sm) protein	2.289	1.194678731	9.16632114438E-8	1.47937833874E-7	yes	up
PAS_chr1-4_0407	Helix-loop-helix protein that binds the motif CACRTG	3.097	1.630642069	5.53851859579E-150	6.66344737278E-149	yes	up
PAS_chr3_0140	ER localized, heme-binding peroxidase involved in the degradation of heme	2.273	1.184526592	3.51048685068E-73	2.35704117117E-72	yes	up
PAS_chr1-3_0280	Hypothetical protein	2.037	1.026401276	3.09470169877E-28	9.94457178473E-28	yes	up
PAS_chr1-3_0309	uncharacterized protein	3.275	1.711305563	1.16342340012E-239	2.01060353237E-238	yes	up
PAS_chr2-1_0504	Peroxisomal membrane protein	2.291	1.196080086	5.77422571681E-63	3.35318488797E-62	yes	up
PAS_chr4_0152	Hypothetical protein	2.033	1.0234454	8.2038596187E-156	1.02121806986E-154	yes	up
PAS_chr3_0422	Hypothetical protein	2.041	1.02913217	7.09990871069E-69	4.50257766785E-68	yes	up
PAS_chr4_0154	Hypothetical protein	2.063	1.0445455	1.64017107157E-112	1.58015714922E-111	yes	up
PAS_chr3_0040	Mitochondrial inner membrane transporter, exports 2-oxoadipate and 2-oxoglutarate from the mitochond	2.458	1.297301029	8.78801236444E-100	7.51614186748E-99	yes	up
PAS_chr3_0697	Hypothetical protein	2.334	1.222788973	5.17532465988E-62	2.97448088166E-61	yes	up
PAS_chr3_0251	uncharacterized protein	3.724	1.896979824	0	0	yes	up
PAS_chr3_0306	1,3-beta-glucanosyltransferase	2.391	1.257682493	3.46009306672E-234	5.83919732635E-233	yes	up
PAS_chr4_0286	Protein involved in the transcription of 35S rRNA genes by RNA polymerase I	2.994	1.581837608	0	0	yes	up
PAS_chr3_0500	Hypothetical protein	3.153	1.65671338	1.89744881736E-44	8.26170571647E-44	yes	up
PAS_chr1-4_0234	ATP phosphoribosyltransferase (hexameric enzyme) catalyzes the first step in histidine biosynthesis	2.042	1.029745027	3.8141218825E-62	2.19716139142E-61	yes	up
PAS_chr2-2_0343	Vacuolar transporter, exports large neutral amino acids from the vacuole	4.677	2.225633576	0	0	yes	up
PAS_chr1-1_0454	SaGa associated Factor 29kDa	2.131	1.091783337	2.08039616226E-50	1.00502519693E-49	yes	up
PAS_chr2-2_0052	uncharacterized protein	2.269	1.182254162	8.45974356613E-50	4.03261141176E-49	yes	up
PAS_chr4_0587	Mitochondrial serine hydroxymethyltransferase	6.003	2.585724658	0	0	yes	up
PAS_chr1-1_0198	Hypothetical protein	2.581	1.368098822	9.49883299115E-277	1.80945572396E-275	yes	up
PAS_chr1-4_0247	uncharacterized protein	2.29	1.195228394	2.16032143092E-124	2.3066361945E-123	yes	up
PAS_chr4_0857	Hypothetical protein	2.311	1.208807498	1.25666717694E-237	2.15692123987E-236	yes	up
PAS_chr3_0937	uncharacterized protein	2.655	1.408671763	5.00233018048E-282	9.67566095293E-281	yes	up
PAS_chr1-1_0293	Cell wall protein that functions in the transfer of chitin to beta(1-6)glucan	3.45	1.786451919	0	0	yes	up
PAS_chr3_0218	Diacylglycerol acyltransferase	2.147	1.102453935	3.49375582997E-148	4.18335668308E-147	yes	up
PAS_chr2-1_0609	protein of OPT family transporter	2.165	1.114038047	6.38181104109E-270	1.19309025002E-268	yes	up
PAS_chr3_0667	Adenylylsulfate kinase, required for sulfate assimilation and involved in methionine metabolism	2.528	1.338068694	1.0230088754E-70	6.59578414666E-70	yes	up
PAS_chr1-1_0008	uncharacterized protein	2.955	1.563289209	0	0	yes	up
PAS_chr3_0766	Subunit of the heme-activated, glucose-repressed Hap2p/3p/4p/5p CCAAT-binding complex	2.686	1.425310008	1.9829788947E-83	1.48178318892E-82	yes	up
PAS_chr1-1_0074	Essential protein of the inner mitochondrial membrane, peripherally localized	2.091	1.064482703	9.29299925222E-12	1.80025012478E-11	yes	up
PAS_chr1-4_0601	Plasma membrane arginine permease, requires phosphatidyl ethanolamine (PE) for localization	2.228	1.156020287	9.31531486269E-225	1.49670026979E-223	yes	up
PAS_chr2-2_0204	Putative phosphopantothenoylcysteine synthetase (PPCS)	4.016	2.005837359	0	0	yes	up
PAS_chr4_0850	Hypothetical protein	2.322	1.215277794	1.05755921543E-204	1.59235487856E-203	yes	up
PAS_chr2-1_0862	uncharacterized protein	4.04	2.014307348	0	0	yes	up
PAS_chr3_0250	Subunit of a histone deacetylase complex	2.154	1.107351082	9.99150148305E-31	3.38365393658E-30	yes	up
PAS_chr1-4_0295	Alpha subunit of fatty acid synthetase	2.306	1.205508647	0	0	yes	up
PAS_chr2-1_0311	NAD(+)-dependent glutamate dehydrogenase, degrades glutamate to ammonia and alpha-ketoglutarate	2.755	1.461930936	0	0	yes	up
PAS_chr2-1_0383	L-homoserine-O-acetyltransferase, catalyzes the conversion of homoserine to O-acetyl homoserine	3.204	1.679746123	0	0	yes	up
PAS_FragB_0025	Phytanoyl-CoA dioxygenase domain-containing protein	8.857	3.146772494	0	0	yes	up
PAS_chr4_0858	Hypothetical protein	2.234	1.159535751	0	0	yes	up
PAS_chr2-2_0344	Mitochondrial inner membrane protein	2.723	1.445441647	8.17565390292E-58	4.43053485752E-57	yes	up
PAS_chr1-4_0059	RNA-binding protein that interacts with the C-terminal domain of the RNA polymerase II large subunit	2.245	1.166434028	9.49831573314E-291	1.87321685576E-289	yes	up
PAS_chr1-1_0033	Hypothetical protein	2.691	1.427963352	5.14715740513E-281	9.91764543693E-280	yes	up
PAS_chr1-4_0198	Pantothenate synthase, also known as pantoate-beta-alanine ligase	2.807	1.489186627	1.04394213424E-233	1.74999499769E-232	yes	up
PAS_chr2-1_0233	Cytoplasmic RNA-binding protein, contains an RNA recognition motif (RRM)	3.645	1.865789195	0	0	yes	up
PAS_chr4_0416	Alanine:glyoxylate aminotransferase (AGT), catalyzes the synthesis of glycine from glyoxylate	2.139	1.097264878	1.62421597312E-165	2.12713076272E-164	yes	up
PAS_chr3_1084	Sulfite reductase beta subunit, involved in amino acid biosynthesis, transcription repressed by meth	3.166	1.662475133	0	0	yes	up

PAS_chr4_0153	Phospholipase B (lysophospholipase) involved in phospholipid metabolism	3.133	1.64746334	0	0	yes	up
PAS_chr2-1_0070	Phosphatase that is highly specific for ADP-ribose 1''-phosphate, a tRNA splicing metabolite	2.196	1.134936647	2.56685198569E-51	1.25205612377E-50	yes	up
PAS_chr2-1_0088	Hypothetical protein	2.523	1.335295451	0	0	yes	up
PAS_chr2-2_0072	uncharacterized protein	2.164	1.113860769	1.80579217981E-171	2.40884054967E-170	yes	up
PAS_chr1-4_0676	uncharacterized protein	2.053	1.03757777	2.95067203612E-46	1.3296531962E-45	yes	up
PAS_chr2-2_0355	Mitochondrial inner membrane carnitine transporter	11.424	3.514044802	0	0	yes	up
PAS_chr4_0249	putative transcription factor	2.165	1.114235738	2.8621383691E-7	4.51496043232E-7	yes	up
PAS_chr1-4_0675	uncharacterized protein	2.083	1.058604501	1.20824093292E-196	1.73606961476E-195	yes	up
PAS_c121_0017	Hypothetical protein	2.518	1.332389479	2.08890732216E-34	7.61240211823E-34	yes	up
PAS_chr4_0974	uncharacterized protein	2.419	1.274675635	3.74230738907E-251	6.62678304917E-250	yes	up
PAS_chr4_0828	Myo-inositol transporter with strong similarity to the minor myo-inositol transporter Itr2p	20.137	4.331751403	0	0	yes	up
PAS_chr3_0675	Asparagine synthetase, isozyme of Asn1p	11.607	3.536968427	0	0	yes	up
PAS_chr3_0067	Hypothetical protein	2.127	1.088739888	5.14077476125E-20	1.31100183947E-19	yes	up
PAS_chr1-1_0097	Cyclin, interacts with Pho85p cyclin-dependent kinase (Cdk), induced by Gcn4p at level of transcript	2.122	1.085430974	2.36698770306E-98	1.99724516086E-97	yes	up
PAS_chr3_0023	Putative transporter, member of the sugar porter family	7.069	2.821535795	0	0	yes	up
PAS_chr1-1_0158	Putative transmembrane protein involved in export of ammonia, a starvation signal	46.343	5.53428735	0	0	yes	up
PAS_chr4_0488	Mitochondrial phosphate carrier, imports inorganic phosphate into mitochondria	4.276	2.096160556	0	0	yes	up
PAS_FragB_0024	uncharacterized protein	5.774	2.529448823	0	0	yes	up
PAS_chr2-1_0874	uncharacterized protein	10.695	3.418910152	0	0	yes	up
PAS_chr3_0848	High affinity polyamine permease	2.649	1.405345293	0	0	yes	up
PAS_chr2-1_0160	Cobalamin-independent methionine synthase, involved in amino acid biosynthesis	2.774	1.471788258	0	0	yes	up
PAS_chr3_0770	Bifunctional enzyme of the 'de novo' purine nucleotide biosynthetic pathway	2.103	1.072546574	0	0	yes	up
PAS_chr3_0105	Hypothetical protein	2.132	1.092417628	1.15520449816E-16	2.6576044928E-16	yes	up
PAS_chr1-1_0218	Hypothetical protein	2.184	1.127127958	2.35754313488E-201	3.5077172856E-200	yes	up
PAS_chr4_0330	Methionine and cysteine synthase (O-acetyl homoserine-O-acetyl serine sulfhydrylase)	2.338	1.225575612	0	0	yes	up
PAS_chr1-4_0602	N(6)-adenine-specific DNA methyltransferase	2.246	1.167645612	4.80926434135E-134	5.52186994809E-133	yes	up
PAS_chr1-4_0074	Outer mitochondrial carnitine acetyltransferase, minor ethanol-inducible enzyme	10.471	3.388384029	0	0	yes	up
PAS_chr1-1_0157	uncharacterized protein	2.548	1.349471326	1.79553282524E-181	2.48753018681E-180	yes	up
PAS_chr3_1015	Protein required for growth of cells lacking the mitochondrial genome	2.664	1.41379602	2.41840621379E-25	7.20507396276E-25	yes	up
PAS_FragB_0029	uncharacterized protein	4.096	2.034382431	3.99862123387E-142	4.69837994979E-141	yes	up
PAS_chr1-3_0052	Biotin synthase, catalyzes the conversion of dethiobiotin to biotin	6.586	2.719369053	0	0	yes	up
PAS_chr1-4_0338	Isocitrate lyase, catalyzes the formation of succinate and glyoxylate from isocitrate	5.088	2.347090694	0	0	yes	up
PAS_chr3_0407	Putative transcription factor containing a zinc finger	2.084	1.05948556	0	0	yes	up
PAS_chr3_0778	Hypothetical protein	3.489	1.802660803	0	0	yes	up
PAS_chr2-1_0782	uncharacterized protein	2.42	1.274827632	0	0	yes	up
PAS_chr3_1023	Hypothetical protein	3.383	1.758392047	4.4411892649E-169	5.86213669637E-168	yes	up
PAS_chr4_0540	Zinc cluster transcriptional activator	2.898	1.535256708	0	0	yes	up
PAS_chr4_0688	Mitochondrial succinate-fumarate transporter	3.648	1.867271161	0	0	yes	up
PAS_FragB_0023	Vitamin H transporter 1	12.277	3.617864673	0	0	yes	up
PAS_chr1-1_0449	uncharacterized protein	3.61	1.852038648	0	0	yes	up
PAS_chr4_0665	Gamma-glutamyl phosphate reductase, catalyzes the second step in proline biosynthesis	2.198	1.135988718	0	0	yes	up
PAS_chr1-3_0024	Succinate semialdehyde dehydrogenase	2.068	1.048518727	0	0	yes	up
PAS_chr4_0579	Intracellular sporulation-specific glucoamylase involved in glycogen degradation	4.735	2.24344095	0	0	yes	up
PAS_chr1-1_0109	Hypothetical protein	2.105	1.073790668	1.237354032E-252	2.20661469039E-251	yes	up
PAS_chr3_0037	Cysteine desulfurase involved in iron-sulfur cluster (Fe/S) biogenesis	2.093	1.065394212	0	0	yes	up
PAS_chr3_0253	Acetyl-coA hydrolase	6.555	2.712550238	0	0	yes	up
PAS_chr1-3_0075	Hypothetical protein	2.997	1.583721022	1.46309399943E-5	2.11494674421E-5	yes	up
PAS_chr3_0528	Saccharopine dehydrogenase (NADP+, L-glutamate-forming)	2.026	1.018444591	0	0	yes	up
PAS_chr2-1_0767	acetate--CoA ligase	15.389	3.943841223	0	0	yes	up
PAS_chr2-2_0394	Hypothetical protein	2.054	1.038348874	7.76678471424E-307	1.58777074504E-305	yes	up
PAS_chr2-1_0785	uncharacterized protein	3.157	1.6584624	0	0	yes	up
PAS_chr4_0815	Mitochondrial malate dehydrogenase, catalyzes interconversion of malate and oxaloacetate	7.79	2.961585382	0	0	yes	up
PAS_chr3_0482	Putative alanine transaminase (glutamic pyruvic transaminase)	3.427	1.776801057	0	0	yes	up
PAS_chr3_0771	Hypothetical protein	2.291	1.195858912	0	0	yes	up
PAS_chr1-1_0085	Hypothetical protein	2.366	1.242696751	0	0	yes	up
PAS_chr3_0303	Aspartic protease, attached to the plasma membrane via a glycosylphosphatidylinositol (GPI) anchor	6.359	2.668730886	0	0	yes	up
PAS_chr3_0876	S-adenosylmethionine synthetase	3.226	1.689592333	0	0	yes	up
PAS_chr4_0191	Malate synthase	3.002	1.586088854	0	0	yes	up
PAS_chr4_0972	uncharacterized protein	3.18	1.669232035	0	0	yes	up
PAS_chr1-4_0481	Hypothetical protein	11.369	3.50705372	0	0	yes	up
PAS_chr2-1_0351	High affinity methionine permease	4.225	2.078895324	0	0	yes	up

PAS_chr2-2_0403	Hypothetical protein	21.816	4.447318834	0	0	yes	up
PAS_FragB_0074	uncharacterized protein	3.95	1.981838484	0	0	yes	up
PAS_chr3_0066	uncharacterized protein	3.683	1.880756699	0	0	yes	up
PAS_chr1-1_0166	RNA polymerase subunit, found in RNA polymerase complexes I, II, and III	2.093	1.06535627	1.45070140388E-12	2.90776299726E-12	yes	up
PAS_chr2-1_0853	uncharacterized protein	4.157	2.055402523	0	0	yes	up
PAS_chr3_0440	Lactate transporter	2.231	1.157410332	0	0	yes	up
PAS_chr3_0227	uncharacterized protein	2.782	1.475920472	0	0	yes	up
PAS_chr1-4_0421	Homocitrate synthase isozyme, catalyzes the condensation of acetyl-CoA and alpha-ketoglutarate	3.569	1.835356399	0	0	yes	up
PAS_chr4_0164	Hypothetical protein	2.12	1.084150012	6.33963637385E-9	1.08664046776E-8	yes	up
PAS_chr1-1_0475	uncharacterized protein	7.747	2.953674995	0	0	yes	up
PAS_chr1-3_0264	Cytoplasmic peptidyl-prolyl cis-trans isomerase (cyclophilin)	2.73	1.448833524	0	0	yes	up
PAS_chr4_0287	Dicarboxylic amino acid permease	2.756	1.462376718	0	0	yes	up
PAS_chr1-1_0433	Mitochondrial peroxiredoxin (1-Cys Prx) with thioredoxin peroxidase activity	2.383	1.252874032	0	0	yes	up
PAS_chr1-1_0108	Hypothetical protein	2.71	1.438034237	0	0	yes	up
PAS_chr1-4_0393	Member of a stationary phase-induced gene family	2.684	1.424431345	0	0	yes	up
PAS_chr2-1_0429	One of two nearly identical (see also HTA1) histone H2A subtypes	3.156	1.658303218	0	0	yes	up
PAS_chr4_0589	Hypothetical protein	3.043	1.605522424	0	0	yes	up
PAS_chr1-3_0104	Aconitase, required for the tricarboxylic acid (TCA) cycle and also independently required for mitoc	2.197	1.135793571	0	0	yes	up
PAS_chr4_0627	Plasma membrane localized protein that protects membranes from desiccation	2.434	1.283380136	0	0	yes	up
PAS_FragB_0061	Phosphoenolpyruvate carboxykinase	0.331	-1.59623722	0	0	yes	down
PAS_chr2-1_0313	Bifunctional enzyme with alcohol dehydrogenase and glutathione-dependent formaldehyde dehydrogenase	0.444	-1.1718305	0	0	yes	down
PAS_chr1-1_0407	uncharacterized protein	0.282	-1.827222601	0	0	yes	down
PAS_chr4_0407	uncharacterized protein	0.49	-1.029094831	1.65199115637E-267	3.03206698007E-266	yes	down
PAS_chr3_0230	ATPase involved in protein folding and the response to stress	0.194	-2.36403772	0	0	yes	down
PAS_chr2-1_0437	Glyceraldehyde-3-phosphate dehydrogenase, isozyme 3, involved in glycolysis and gluconeogenesis	0.19	-2.393700114	0	0	yes	down
PAS_chr4_0422	Subunit VIb of cytochrome c oxidase	0.462	-1.112693464	2.46851155811E-62	1.42527492833E-61	yes	down
PAS_chr2-2_0265	Hypothetical protein	0.375	-1.413888431	1.47515818579E-27	4.64822714057E-27	yes	down
PAS_chr2-1_0361	Subunit IV of cytochrome c oxidase	0.379	-1.400875266	0	0	yes	down
PAS_chr2-1_0363	Subunit VIa of cytochrome c oxidase, which is the terminal member of the mitochondrial inner membrane	0.343	-1.544771261	0	0	yes	down
PAS_chr3_0615	Subunit Va of cytochrome c oxidase	0.427	-1.228983165	0	0	yes	down
PAS_chr2-1_0746	Hypothetical protein	0.477	-1.067330669	7.01973704339E-10	1.25720290567E-9	yes	down
PAS_chr2-2_0337	Transaldolase, enzyme in the non-oxidative pentose phosphate pathway	0.482	-1.053998832	0	0	yes	down
PAS_chr3_0648	Thiazole synthase, catalyzes formation of the thiazole moiety of thiamine pyrophosphate	0.365	-1.455338	0	0	yes	down
PAS_chr3_0824	Subunit VI of cytochrome c oxidase	0.469	-1.090905766	9.70622531186E-177	1.32283488058E-175	yes	down
PAS_chr1-1_0289	Hypothetical protein	0.445	-1.1696337	0	0	yes	down
PAS_chr2-2_0200	One of two identical histone H4 proteins (see also HHF2)	0.47	-1.088002791	1.78440517973E-56	9.47600174113E-56	yes	down
PAS_chr1-4_0292	3-phosphoglycerate kinase	0.092	-3.447967372	0	0	yes	down
PAS_chr1-4_0367	C-5 sterol desaturase, catalyzes the introduction of a C-5(6) double bond into episterol	0.167	-2.578344717	0	0	yes	down
PAS_chr3_1206	uncharacterized protein	0.496	-1.011912533	0	0	yes	down
PAS_chr4_0624	Non-essential protein of unknown function required for transcriptional induction	0.175	-2.517239843	0	0	yes	down
PAS_chr1-1_0422	Hypothetical protein	0.257	-1.962551624	0	0	yes	down
PAS_chr3_0082	Enolase I, a phosphopyruvate hydratase that catalyzes the conversion of 2-phosphoglycerate to phosph	0.232	-2.109467743	0	0	yes	down
PAS_chr1-3_0102	Heat shock protein that cooperates with Ydj1p (Hsp40) and Ssa1p (Hsp70)	0.215	-2.215491936	0	0	yes	down
PAS_chr3_0826	Tetrameric phosphoglycerate mutase	0.164	-2.605749209	0	0	yes	down
PAS_chr3_0951	Triose phosphate isomerase, abundant glycolytic enzyme	0.291	-1.781984974	0	0	yes	down
PAS_chr1-1_0072	Fructose 1,6-bisphosphate aldolase, required for glycolysis and gluconeogenesis	0.094	-3.416315531	0	0	yes	down
PAS_chr3_0456	Glycolytic enzyme phosphoglucose isomerase	0.344	-1.537860326	0	0	yes	down
PAS_chr3_1209	uncharacterized protein	0.323	-1.630961937	0	0	yes	down
PAS_chr3_0411	Hypothetical protein	0.348	-1.521878653	7.85676366161E-209	1.19732316528E-207	yes	down
PAS_chr3_0188	uncharacterized protein	0.051	-4.287961311	0	0	yes	down
PAS_chr3_0362	Hypothetical protein	0.391	-1.354273429	0	0	yes	down
PAS_chr4_0953	uncharacterized protein	0.35	-1.515232019	1.21467837066E-152	1.49721017796E-151	yes	down
PAS_chr2-2_0009	Low-affinity Fe(II) transporter of the plasma membrane	0.145	-2.782066834	0	0	yes	down
PAS_chr3_0053	C-4 methyl sterol oxidase, catalyzes the first of three steps required to remove two C-4 methyl grou	0.295	-1.758942845	0	0	yes	down
PAS_chr1-4_0484	mitochondrial 54S ribosomal protein YmL31	0.416	-1.26457492	5.64712564044E-54	2.89494340936E-53	yes	down
PAS_chr1-1_0434	Zinc finger protein involved in control of meiosis	0.465	-1.106157552	0	0	yes	down
PAS_chr2-1_0210	Hypothetical protein	0.107	-3.21899136	0	0	yes	down
PAS_chr3_0535	Sphingolipid alpha-hydroxylase	0.301	-1.731054501	0	0	yes	down
PAS_chr2-2_0092	Chaperone that specifically facilitates the assembly of cytochrome c oxidase	0.447	-1.161888667	9.87382065786E-24	2.82937003353E-23	yes	down
PAS_chr1-1_0381	bZIP transcription factor (ATF/CREB1 homolog) that regulates the unfolded protein response	0.419	-1.254771221	0	0	yes	down
PAS_chr1-4_0055	Clavaminate synthase	0.475	-1.072503229	0	0	yes	down

PAS_chr2-1_0140	ATPase involved in protein import into the ER, also acts as a chaperone to mediate protein folding i	0.293	-1.769068037	0	0	yes	down
PAS_c131_0006	Hypothetical protein	0.266	-1.911363854	0	0	yes	down
PAS_chr3_0868	Fructose-1,6-bisphosphatase, key regulatory enzyme in the gluconeogenesis pathway	0.242	-2.046180114	0	0	yes	down
PAS_chr1-4_0394	Ammonium permease involved in regulation of pseudohyphal growth	0.151	-2.728665651	0	0	yes	down
PAS_chr1-3_0122	UDP-glucose pyrophosphorylase (UGPase), catalyses the reversible formation of UDP-Glc	0.196	-2.353049095	0	0	yes	down
PAS_chr2-1_0893	uncharacterized protein	0.376	-1.409794945	3.33580889476E-115	3.30231947476E-114	yes	down
PAS_chr1-1_0378	Putative transmembrane protein involved in export of ammonia	0.036	-4.782097846	0	0	yes	down
PAS_chr3_0425	Sphinganine C4-hydroxylase	0.412	-1.278523095	0	0	yes	down
PAS_chr1-4_0264	Phosphoglucomutase	0.303	-1.720964994	0	0	yes	down
PAS_chr2-2_0470	uncharacterized protein	0.096	-3.377991065	0	0	yes	down
PAS_chr2-1_0402	Alpha subunit of heterooctameric phosphofructokinase involved in glycolysis	0.137	-2.872403364	0	0	yes	down
PAS_chr1-4_0518	Hypothetical protein	0.141	-2.826701702	0	0	yes	down
PAS_chr1-4_0570	High-affinity glucose transporter of the major facilitator superfamily	0.092	-3.449584866	0	0	yes	down
PAS_chr4_0821	Alcohol oxidase	0.465	-1.104152703	0	0	yes	down
PAS_chr2-2_0236	Hypothetical protein	0.11	-3.190351538	0	0	yes	down
PAS_chr2-1_0769	Pyruvate kinase	0.137	-2.865888936	0	0	yes	down
PAS_chr2-2_0311	3-hydroxyanthranilic acid dioxygenase, required for the de novo biosynthesis of NAD from tryptophan	0.2	-2.321691024	0	0	yes	down
PAS_chr4_0847	Self-glucosylating initiator of glycogen synthesis, also glucosylates n-dodecyl-beta-D-maltoside	0.218	-2.195898245	0	0	yes	down
PAS_chr1-1_0448	uncharacterized protein	0.234	-2.093913106	0	0	yes	down
PAS_chr2-1_0324	Oligomeric mitochondrial matrix chaperone	0.347	-1.528250623	0	0	yes	down
PAS_chr1-4_0188	Nitric oxide oxidoreductase, flavohemoglobin involved in nitric oxide detoxification	0.249	-2.004510427	0	0	yes	down
PAS_chr2-2_0137	Cystathionine beta-synthase, catalyzes the synthesis of cystathionine from serine and homocysteine	0.439	-1.18659353	0	0	yes	down
PAS_chr3_0943	Coproporphyrinogen III oxidase, an oxygen requiring enzyme	0.113	-3.142184289	0	0	yes	down
PAS_chr3_0507	C-22 sterol desaturase	0.341	-1.553305193	0	0	yes	down
PAS_chr1-1_0050	Dihydrolipoamide acetyltransferase component (E2) of pyruvate dehydrogenase complex	0.294	-1.765917308	0	0	yes	down
PAS_chr4_0943	uncharacterized protein	0.145	-2.787773964	0	0	yes	down
PAS_chr4_0969	uncharacterized protein	0.26	-1.94122358	0	0	yes	down
PAS_chr4_0433	NADPH-dependent alpha-keto amide reductase	0.398	-1.328064538	2.5495141792E-225	4.1226709991E-224	yes	down
PAS_chr4_0406	Hypothetical protein	0.413	-1.274889559	4.34494237917E-12	8.55548755868E-12	yes	down
PAS_chr2-2_0294	E1 alpha subunit of the pyruvate dehydrogenase (PDH) complex	0.252	-1.989580656	0	0	yes	down
PAS_FragB_0075	Lactose regulatory protein	0.25	-1.999906156	0	0	yes	down
PAS_chr1-1_0482	uncharacterized protein	0.384	-1.382251036	9.71226967893E-131	1.08540009367E-129	yes	down
PAS_chr2-2_0220	Thioredoxin peroxidase, acts as both a ribosome-associated and free cytoplasmic antioxidant	0.335	-1.576665465	4.06776357832E-138	4.72443026221E-137	yes	down
PAS_chr1-4_0168	uncharacterized protein	0.2	-2.319770997	7.62635234443E-269	1.41523711956E-267	yes	down
PAS_chr4_0967	uncharacterized protein	0.479	-1.063236823	0	0	yes	down
PAS_chr1-4_0160	Multifunctional enzyme HIS4	0.342	-1.548289586	0	0	yes	down
PAS_chr1-3_0229	uncharacterized protein	0.427	-1.228515636	6.29946248429E-175	8.51612818104E-174	yes	down
PAS_chr2-1_0033	Nitrogen catabolite repression transcriptional regulator that acts by inhibition of GLN3 transcripti	0.459	-1.123431777	6.06863674696E-116	6.04340083177E-115	yes	down
PAS_chr3_0547	Endoplasmic reticulum membrane protein	0.318	-1.653078823	2.56637257065E-71	1.66747902556E-70	yes	down
PAS_chr2-2_0199	One of two identical histone H3 proteins (see also HHT2)	0.254	-1.975356019	1.40377849559E-98	1.18648774022E-97	yes	down
PAS_chr1-1_0011	Thiol oxidase required for oxidative protein folding in the endoplasmic reticulum	0.467	-1.097456794	1.73415955026E-199	2.55750392324E-198	yes	down
PAS_chr3_0030	Hypothetical protein	0.433	-1.208314939	1.73618122558E-279	3.33254022269E-278	yes	down
PAS_chr1-1_0423	Hypothetical protein	0.499	-1.002519897	8.30314520356E-44	3.57504428328E-43	yes	down
PAS_chr1-4_0047	Beta subunit of heterooctameric phosphofructokinase involved in glycolysis	0.235	-2.086962571	0	0	yes	down
PAS_chr4_0449	Protein required for the hydroxylation of heme O to form heme A	0.474	-1.076454826	1.86332173181E-174	2.51223726254E-173	yes	down
PAS_chr1-1_0127	Hypothetical protein	0.42	-1.25324083	1.54035394492E-176	2.09363242945E-175	yes	down
PAS_chr1-4_0410	Putative positive regulator of mannosylphosphate transferase (Mnn6p)	0.406	-1.300816202	0	0	yes	down
PAS_chr3_0957	Lanosterol 14-alpha-demethylase, catalyzes the C-14 demethylation of lanosterol	0.407	-1.296029641	4.583559378E-266	8.35171018549E-265	yes	down
PAS_chr3_1091	Protein kinase implicated in activation of the plasma membrane H(+)-ATPase Pma1p	0.392	-1.352321086	0	0	yes	down
PAS_chr2-1_0322	Calnexin	0.31	-1.691957593	0	0	yes	down
PAS_chr1-4_0054	G1 cyclin involved in cell cycle progression	0.275	-1.860449778	0	0	yes	down
PAS_chr2-1_0642	Hypothetical protein	0.241	-2.05246124	0	0	yes	down
PAS_chr1-4_0547	peroxiredoxin	0.149	-2.745049535	0	0	yes	down
PAS_chr2-1_0404	Transcriptional repressor and activator	0.406	-1.299699851	1.27048313019E-245	2.22622287865E-244	yes	down
PAS_chr2-1_0092	Pho85 cyclin of the Pcl1,2-like subfamily, involved in entry into the mitotic cell cycle and regulat	0.173	-2.531342045	0	0	yes	down
PAS_chr1-4_0593	E1 beta subunit of the pyruvate dehydrogenase (PDH) complex	0.258	-1.952071394	0	0	yes	down
PAS_chr1-4_0561	Hexokinase-2	0.382	-1.389475532	3.13955100251E-219	5.02828088905E-218	yes	down
PAS_chr3_0834	Transketolase, similar to Tkl2p	0.253	-1.980998239	0	0	yes	down
PAS_chr4_0927	uncharacterized protein	0.441	-1.18101094	8.40452558782E-182	1.1675789829E-180	yes	down
PAS_chr2-1_0745	uncharacterized protein	0.474	-1.076203537	8.94917025357E-134	1.02284948194E-132	yes	down
PAS_chr3_0832	Transketolase, similar to Tkl2p	0.217	-2.206810476	0	0	yes	down

PAS_chr4_0197	Small subunit of the ribonucleotide-diphosphate reductase (RNR) complex	0.378	-1.402775972	2.99343181567E-152	3.67169965878E-151	yes	down
PAS_chr2-1_0360	uncharacterized protein	0.411	-1.283190745	4.73437794359E-107	4.37668872763E-106	yes	down
PAS_chr3_0693	Tetrameric phosphoglycerate mutase	0.242	-2.047706524	2.94170635969E-199	4.31307326032E-198	yes	down
PAS_chr2-1_0770	Uridine/cytidine kinase, component of the pyrimidine ribonucleotide salvage pathway	0.275	-1.860319442	0	0	yes	down
PAS_chr2-2_0064	Protein of the SUN family (Sim1p, Uth1p, Nca3p, Sun4p) that may participate in DNA replication	0.497	-1.008175674	2.73400278052E-72	1.80437007654E-71	yes	down
PAS_chr3_0604	Putative ribokinase	0.495	-1.015930865	2.28731421253E-54	1.18342625255E-53	yes	down
PAS_chr3_0976	uncharacterized protein	0.247	-2.018225659	1.04464353322E-164	1.35749672573E-163	yes	down
PAS_chr1-4_0364	Rab GTPase-activating protein	0.494	-1.018748197	1.73088464991E-71	1.12754130886E-70	yes	down
PAS_chr3_0725	Protein with a role in ubiquinone (Coenzyme Q) biosynthesis	0.447	-1.162640609	1.02709892546E-57	5.56004359112E-57	yes	down
PAS_chr4_0807	Hypothetical protein	0.324	-1.624825274	2.78338698292E-85	2.12407483112E-84	yes	down
PAS_chr4_0243	uncharacterized protein	0.426	-1.230256595	3.76864202382E-156	4.70285378109E-155	yes	down
PAS_chr4_0806	NADP-cytochrome P450 reductase	0.445	-1.168848506	1.03401485513E-142	1.22067152734E-141	yes	down
PAS_chr3_0124	Squalene epoxidase, catalyzes the epoxidation of squalene to 2,3-oxidosqualene	0.213	-2.229641457	0	0	yes	down
PAS_chr2-1_0563	Protein that activates Urm1p before its conjugation to proteins (urmylation)	0.383	-1.385785886	5.13084655857E-127	5.57300806545E-126	yes	down
PAS_chr2-2_0299	Plasma membrane ATP binding cassette (ABC) transporter	0.49	-1.028956857	2.95131150813E-240	5.11798123254E-239	yes	down
PAS_chr1-3_0070	Mitochondrial inorganic pyrophosphatase	0.491	-1.026478771	3.01416125336E-35	1.11868759728E-34	yes	down
PAS_chr3_0233	Proliferating cell nuclear antigen (PCNA)	0.317	-1.656725671	4.62760148537E-90	3.66491462518E-89	yes	down
PAS_chr3_1208	uncharacterized protein	0.179	-2.484593978	0	0	yes	down
PAS_chr1-1_0130	uncharacterized protein	0.476	-1.070472481	2.09146888806E-156	2.61641717365E-155	yes	down
PAS_chr1-3_0003	Hypothetical protein	0.436	-1.196389064	1.52277138728E-87	1.19098247382E-86	yes	down
PAS_chr3_0154	uncharacterized protein	0.3	-1.736365726	2.48799845654E-227	4.08893602547E-226	yes	down
PAS_chr1-1_0479	uncharacterized protein	0.263	-1.925988739	1.06654855492E-70	6.86769869744E-70	yes	down
PAS_chr4_0498	Suppressor of sphingoid long chain base (LCB) sensitivity of an LCB-lyase mutation	0.353	-1.50035918	1.99990787972E-86	1.54256698268E-85	yes	down
PAS_chr2-1_0149	Protein involved in DNA replication	0.417	-1.26177944	3.56534709156E-157	4.48253263087E-156	yes	down
PAS_chr2-2_0489	uncharacterized protein	0.433	-1.207423673	1.69949555437E-22	4.70896040931E-22	yes	down
PAS_chr2-2_0021	Hypothetical protein	0.239	-2.063960802	1.90848227962E-214	2.98995557141E-213	yes	down
PAS_chr4_0055	Protoporphyrinogen oxidase	0.475	-1.074959816	6.44947603746E-58	3.51402112594E-57	yes	down
PAS_chr1-1_0013	Hypothetical protein	0.485	-1.043507206	3.13784603603E-47	1.43066434408E-46	yes	down
PAS_chr3_0076	uncharacterized protein	0.323	-1.62817625	6.26405738239E-68	3.91815231045E-67	yes	down
PAS_chr2-2_0333	Putative kinase	0.275	-1.860603705	3.15315503715E-101	2.72460767729E-100	yes	down
PAS_chr3_0420	Hypothetical protein	0.406	-1.301650023	9.76426990795E-113	9.46137059096E-112	yes	down
PAS_chr3_0670	Cytoplasmic thioredoxin isoenzyme of the thioredoxin system	0.483	-1.048547194	0.000738082566682	0.000969395985334	yes	down
PAS_chr1-1_0282	One of two large regulatory subunits of ribonucleotide-diphosphate reductase	0.338	-1.564331799	6.63181697172E-192	9.36837290753E-191	yes	down
PAS_chr1-1_0377	RNA exonuclease	0.331	-1.593834421	2.01710264996E-103	1.79222777856E-102	yes	down
PAS_chr2-2_0065	Plasma membrane multidrug transporter of the major facilitator superfamily	0.338	-1.563185363	1.61410976671E-80	1.17302861515E-79	yes	down
PAS_chr2-1_0186	Alpha-tubulin	0.3	-1.734945691	6.66070365842E-91	5.32538612054E-90	yes	down
PAS_chr3_0225	Soluble fumarate reductase	0.074	-3.760978381	0	0	yes	down
PAS_chr4_0575	Hypothetical protein	0.452	-1.146995108	1.13432669634E-45	5.02159239073E-45	yes	down
PAS_chr3_0734	Hypothetical protein	0.497	-1.007946503	4.83317301816E-26	1.47577578071E-25	yes	down
PAS_chr2-1_0016	2-deoxyglucose-6-phosphate phosphatase, similar to Dog2p, member of a family of low molecular weight	0.046	-4.439340228	0	0	yes	down
PAS_chr3_0399	Minor sphingoid long-chain base kinase	0.274	-1.867272308	3.63025719513E-94	2.96372783025E-93	yes	down
PAS_chr4_0977	uncharacterized protein	0.459	-1.122110123	9.20179450379E-46	4.08076054317E-45	yes	down
PAS_chr4_0008	Low-affinity Fe(II) transporter of the plasma membrane	0.307	-1.701681197	4.51110587105E-75	3.08657842524E-74	yes	down
PAS_chr1-3_0014	Flavin-containing monooxygenase, localized to the cytoplasmic face of the ER membrane	0.475	-1.072964167	1.00478253974E-19	2.54049843758E-19	yes	down
PAS_chr3_0821	uncharacterized protein	0.282	-1.826030994	1.12018314796E-32	3.95049162069E-32	yes	down
PAS_chr2-1_0700	uncharacterized protein	0.296	-1.758726093	5.88361738957E-112	5.64670073514E-111	yes	down
PAS_chr4_0643	Hypothetical protein	0.153	-2.707469737	9.42786646016E-283	1.83060773854E-281	yes	down
PAS_chr2-2_0117	Hypothetical protein	0.389	-1.363811558	8.6275022054E-15	1.87015985306E-14	yes	down
PAS_FragB_0039	DNA-dependent ATPase	0.126	-2.986052496	0	0	yes	down
PAS_chr2-1_0425	Hypothetical protein	0.214	-2.225703764	2.37494151972E-113	2.31914192285E-112	yes	down
PAS_chr2-1_0163	Catalytic subunit of DNA polymerase delta	0.454	-1.139578369	1.94193862175E-49	9.19586565801E-49	yes	down
PAS_chr2-1_0403	Hypothetical protein	0.2	-2.323169574	3.67324406463E-9	6.36552184736E-9	yes	down
PAS_chr1-1_0340	Protein kinase involved in bud growth and assembly of the septin ring	0.451	-1.14998319	9.1423388158E-50	4.34974663242E-49	yes	down
PAS_chr4_0533	Beta-tubulin	0.258	-1.95318935	8.55536174998E-59	4.72282263893E-58	yes	down
PAS_chr3_1191	uncharacterized protein	0.457	-1.128620786	1.74826169327E-14	3.75085667896E-14	yes	down
PAS_chr4_0034	Subunit of heterotrimeric Replication Protein A (RPA)	0.405	-1.305022227	1.41610141852E-10	2.5962719773E-10	yes	down
PAS_chr4_0244	Hypothetical protein	0.429	-1.219664998	1.01432198874E-7	1.63180591215E-7	yes	down
PAS_chr4_0323	Subunit of DNA primase, which is required for DNA synthesis and double-strand break repair	0.423	-1.240707414	4.73284718206E-15	1.03260253703E-14	yes	down
PAS_chr2-1_0320	Protein containing a UCS (UNC-45/CRO1/SHE4) domain	0.369	-1.437681455	1.48552522777E-34	5.43719532056E-34	yes	down
PAS_chr2-1_0276	Hypothetical protein	0.445	-1.166731509	4.7149621507E-21	1.24144212858E-20	yes	down
PAS_chr2-2_0113	Inositol 1-phosphate synthase	0.329	-1.603430394	8.32738226319E-28	2.6405047542E-27	yes	down

PAS_chr2-1_0500	B subunit of DNA polymerase alpha-primase complex	0.47	-1.089372389	3.70927068519E-13	7.59833901256E-13	yes	down
PAS_chr3_0814	Hypothetical protein	0.447	-1.160068772	7.11311132973E-14	1.49610359169E-13	yes	down
PAS_chr3_0811	Hypothetical protein	0.278	-1.848041625	1.77942202711E-24	5.17565840042E-24	yes	down
