# Supplementary material for: Cross Kingdom Metabolic Engineering Paradigm Elevating Sustainable Protein Production
Source: Adv Sci (Weinh). 2026 Jun 23:e17703. Online ahead of print. doi: 10.1002/advs.202517703 (PMC13336901; doi:10.1002/advs.202517703)
Supplement: Supplementary file 2 — Supporting File 2: advs76229‐sup‐0002‐Fig. S3.docx. [file ADVS-9999-e17703-s003.docx]

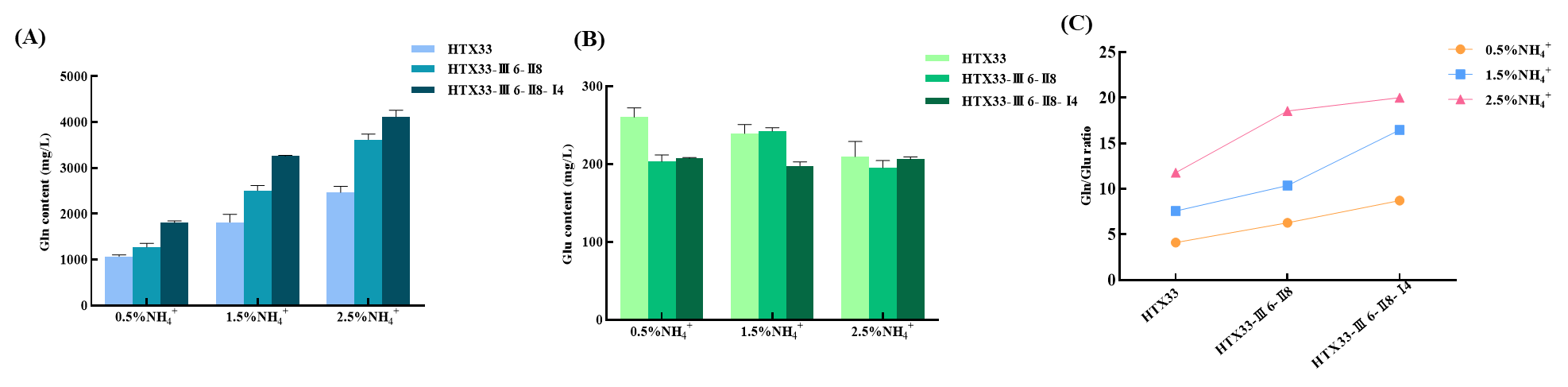
Supplemental Fig.3 Intracellular glutamine (Gln) and glutamate (Glu) contents in wild-type *P. pastoris* HTX33 and ASNS-engineered strains under different ammonium concentrations. (A) Gln content; (B) Glu content. (C) Gln/Glu ratio.
