# Supplementary material for: Cross Kingdom Metabolic Engineering Paradigm Elevating Sustainable Protein Production
Source: Adv Sci (Weinh). 2026 Jun 23:e17703. Online ahead of print. doi: 10.1002/advs.202517703 (PMC13336901; doi:10.1002/advs.202517703)
Supplement: Supplementary file 5 — Supporting File 5: advs76229‐sup‐0005‐Table S3.pdf. [file ADVS-9999-e17703-s001.pdf]

Table S3. Primers used in this study.

Primers	Purpose	Sequences (5'–3') of paired primers
PNSIII-6-up-F/PNSIII-6-up-R	Cloning the upstream region of III-6 site from HTX33	GATTGGAGCTCTTCCACTCCTTGAATC/gaggacaccaagacatttctacaaaaaTGAACTCAAAGACTATCACAAACAGTGTGC
PNSIII-6-down-F/PNSIII-6-down-R	Cloning the downstream region of III-6 site from HTX33	CTTCAGAGTACAGAAGATTAAGTGAGAAAAAAATTGTTCTGATGTGCAACACAAAC/TTGTCATTAGCAGGGTATCAAGAGTTAATCAAGC
PNSII-8-up-F/PNSII-8-up-R	Cloning the upstream region of II-8 site from HTX33	CTGGGACTTTGTTCATTGGATATTTGC/caagacatttctacaaaaaAATTCGCAAGTTTCAGATTGCTTGC
PNSII-8-down-F/PNSII-8-down-R	Cloning the downstream region of II-8 site from HTX33	GTACAGAAGATTAAGTGAGAATCACGGATGTTGATTCCCGTAAC/AAGGATAATGTTGCCTTGCAGTGAATC
PNSI-4-up-F/PNSI-4-up-R	Cloning the upstream region of I-4 site from HTX33	TgcttcattttccttcCCGATAATCGAACT/ccaagacatttctacaaaaaCTCCTAGAACACCTAACATTGTATCACAAC
PNSI-4-down-F/PNSI-4-down-R	Cloning the downstream region of I-4 site from HTX33	TCAGAGTACAGAAGATTAAGTGAGACAGGACGAGATATCAAAATAAAGGGCTC/TGCAGTGAACATAGGCAAAACATACG
ASNS-F/ASNS-R	Cloning ASNS gene from HTX33	tccctatttcaatcaattgaacaactatatgtgtggtatttttgcagcatacagagt/TGGCATTCTGACATCCTCTTGActatttcttaatttcatctacagcggaagc
PGAP-F/PGAP-R	Cloning GAP gene from HTX33	TTTTTGTAGAAATGTCTTGGTGTCCTCG/tgtatgctgcaaaaataccacacatatagttgttcaattgattgaaataggga
TAOX1-F/TAOX1-R	Cloning AOX1 terminator gene from HTX33	TCAAGAGGATGTCAGAATGCCATT/TCTCACTTAATCTTCTGTACTCTGAAGAGG
PNSI-2-up-F/PNSI-2-up-R	Cloning the upstream region of I-2 site from HTX33	TGGTATACCGTAATTTCTCAGAAGG/ttctacaaaaaTCTACGTTTTAAGATCAATCAAATCAC
PNSI-2-down-F/PNSI-2-down-R	Cloning the downstream region of I-2 site from HTX33	ACAGAAGATTAAGTGAGAAGTAACAAAAAATGAAAAAATTAAAGTTTGACACGGACAGG/ATCTGAAGTGAAGTGGAGAGCTG
PNS0158-up-F/PNS0158-up-R	Cloning the upstream region of 0158 site from HTX33	GTACGCCTGAAACAATTATCCTAATCCCCT/GGGTTCAACGTACCACCGAAGG
PNS0158-down-F/PNS0158-down-R	Cloning the downstream region of 0158 site from HTX33	CTGACCACTACAAGAAGCGTAATTAAGT/ACCGTCTAATTTTCTTGTCAGTCTTTCCTAG
0158-F/0158-R	Cloning PAS chr1-1 0158 gene from HTX33	caatcaattgaacaactatATGAGTGAACAAGACTTTGA/TCTGACATCCTCTTGATTAATTACGCTTCTTGTAGTGGTC
zeocin-F/zeocin-R	Cloning zeocin gene from pPICZ-Cas9-gGUT1	GCCTTCGGTGGTACGTTGAACCCgatcccccacacaccatagcttc/TACGCTTCTTGTAGTGGTCAGgcaaattaaagccttcgagcgtc
gPNSIII-6-1F/gPNSIII-6-1R	Cloning PNSIII-6-sgRNA cassette	ACTCCGCTGATGAGTCCGTGAGGACGAAACGAGTAAGCTCGTCCGGAGTTTAATAAAGCATGTCGGGTTTTAGAGCTAGAAATAGCA/ACGGGAAGTCTTTACAGTTT
gPNSIII-6-2F/gPNSIII-6-2R	Cloning PNSIII-6-sgRNA cassette	CTCCTAACTAAAACTGTAAAGACTTCCCGTTTAAACTTTTCTTTTCTTCT/GTTTCGTCCTCACGGACTCATCAGCGGAGTTTTGATTTGTTTAGGTAACT
gPNSII-8-1F/gPNSII-8-1R	Cloning PNSII-8-sgRNA cassette	AGTAACCTGATGAGTCCGTGAGGACGAAACGAGTAAGCTCGTCGTTACTGGATTAGAACGACGAGGGTTTTAGAGCTAGAAATAGCA/ACGGGAAGTCTTTACAGTTT
gPNSII-8-2F/gPNSII-8-2R	Cloning PNSII-8-sgRNA cassette	CTCCTAACTAAAACTGTAAAGACTTCCCGTTTAAACTTTTCTTTTCTTCT/GTTTCGTCCTCACGGACTCATCAGGTTACTTTTGATTTGTTTAGGTAACT
gPNSI-4-1F/gPNSI-4-1R	Cloning PNSI-4-sgRNA cassette	CCACAACTGATGAGTCCGTGAGGACGAAACGAGTAAGCTCGTCTTGTGGCTATGGCTTGAATGAGGGTTTTAGAGCTAGAAATAGCA/ACGGGAAGTCTTTACAGTTT
gPNSI-4-2F/gPNSI-4-2R	Cloning PNSI-4-sgRNA cassette	CTCCTAACTAAAACTGTAAAGACTTCCCGTTTAAACTTTTCTTTTCTTCT/GTTTCGTCCTCACGGACTCATCAGTTGTGGTTTGATTTGTTTAGGTAACT
gPNS0158-1F/gPNS0158-1R	Cloning PNS0158-sgRNA cassette	ACCGAACTGATGAGTCCGTGAGGACGAAACGAGTAAGCTCGTCTTCGGTGGTACGTTGAACCCGTTTTAGAGCTAGAAATAGCA/ACGGGAAGTCTTTACAGTTT
gPNS0158-2F/gPNS0158-2R	Cloning PNS0158-sgRNA cassette	CTCCTAACTAAAACTGTAAAGACTTCCCGTTTAAACTTTTCTTTTCTTCT/GTTTCGTCCTCACGGACTCATCAGTTCGGTTTTGATTTGTTTAGGTAACT
