# Supplementary material for: Cross Kingdom Metabolic Engineering Paradigm Elevating Sustainable Protein Production
Source: Adv Sci (Weinh). 2026 Jun 23:e17703. Online ahead of print. doi: 10.1002/advs.202517703 (PMC13336901; doi:10.1002/advs.202517703)
Supplement: Supplementary file 6 — Supporting File 6: advs76229‐sup‐0006‐Table S4.pdf. [file ADVS-9999-e17703-s002.pdf]

Table S4. Primers used in the RT-qPCR assay

Primers	Purpose	Sequences (5'–3') of paired primers
actin-RT-F/actin-RT-R	RT-qPCR of *actin*	CTCCAATGAACCCTAAATCAAACAG / CGGAAGAGTACAAGGACAAAACG
HTX33-ASNS-R / HTX33-ASNS-R	RT-qPCR of ASNS from *HTX33, HTX33-III6, HTX33-III6-II8, and HTX33-III6-II8-I4*	AGAAAGATCAAGGCTCAGGGTG / ACGCAAGCAATCAGCAAGGT
Cyb-ASNS-F / Cyb-ASNS-R	RT-qPCR of ASNS from *Cyberlindnera fabianii* B, *Cyberlindnera fabianii* C	ATCACCAGATACTTCCAGCCAACT / TCTGGCAGCGATAGAAGCAAT
Pic-mem-ASNS-F / Pic-mem-ASNS-R	RT-qPCR of ASNS from *Pichia membranifaciens* NRRL Y-2026 -6, *Pichia membranifaciens* 027	TGTATTCAGCGACCTTCTTGGC / GCAGAAGTTCCTTACGGTGTCCT
Yar-ASNS-F / Yar-ASNS-R	RT-qPCR of ASNS from *Yarrowia lipolytica* cbs6124-7	ATCTACAACCACCGAATCCTGC / CAAACATTCCGTCGAGCTTCTT
Han-ASNS-F / Han-ASNS-R	RT-qPCR of ASNS from *Hanseniaspora uvarum* XD-23, *Hanseniaspora uvarum strain* SC-12, *Hanseniaspora uvarum isolate* 3Y42	GGTTGCTAAGTTTATTGGTTCCG / CTTGACACCTTGGGCCTTGA
Sac-cere-ASNS-F / Sac-cere-ASNS-R	RT-qPCR of ASNS from *Saccharomyces cerevisiae* LV10, *Saccharomyces cerevisiae* saxapqham-21, *Saccharomyces cerevisiae* kDairy8, *Saccharomyces cerevisiae* J14, *Saccharomyces cerevisiae* SF5-3	CGTATCCCATCCACCCCAGTT / GTTGTCTTCGTTAGCATCAGCATT
Sac-lud-ASNS-F / Sac-lud-ASNS-R	RT-qPCR of ASNS from *Saccharomyces ludwigii* cbs1168, *Saccharomyces ludwigii* 10	AAAGCTGCTCGTAAAGTTGCTAAG / ATTGGAGTAGAGGCTCTGATGGTAG
Sac-kud-ASNS-F / Sac-kud-ASNS-R	RT-qPCR of ASNS from *Saccharomyces kudriavzevii* 1, *Saccharomyces kudriavzevii* B4	GTCTACGATTCTGAAACCGACAAG / AATCAAAGAAGAGTCCAGCCCAC
Klu-ASNS-F / Klu-ASNS-R	RT-qPCR of ASNS from *Kluyveromyces marxianus* 12, *Kluyveromyces marxianus* H5	CGGTTACTCTTGGATTGATGGTC / GGAAGCAGCGGTCTTTTGTG
Kud-ASNS-F / Kud-ASNS-R	RT-qPCR of ASNS from *Pichia kudriavzevii* C3, *Pichia kudriavzevii* 2, *Pichia kudriavzevii* 3, *Pichia kudriavzevii* J2, *Pichia kudriavzevii* H4, *Pichia kudriavzevii* 6	AGTATTTGGACGGTATGTTTGCTTG / GGCACTTGAGTTCGGAGGC
